# Decoding Lusichelins A–E: An In-Depth Look at the Metallophores of *Lusitaniella coriacea* LEGE 07167–Structure, Production, and Functionality

**DOI:** 10.1021/acs.jnatprod.5c00204

**Published:** 2025-05-21

**Authors:** Maria Lígia Sousa, Leonor Ferreira, Dora Ferreira, Abel M. Forero, Raquel Castelo-Branco, Nikoletta Szemerédi, Gabriella Spengler, Jaime Rodríguez, Carlos Jiménez, Pedro N. Leão, Vitor Vasconcelos, Mariana Alves Reis

**Affiliations:** 1 CIIMAR/CIMAR, 367866 Interdisciplinary Centre of Marine and Environmental Research, University of Porto, 4450-208 Matosinhos, Portugal; 2 Departamento de Biologia, Faculdade de Ciências, 26706 Universidade do Porto, Rua do Campo Alegre, Edifício FC4, 4169-007 Porto, Portugal.; 3 CICA−Centro Interdisciplinar de Química e Bioloxía e Departamento de Química, Rua As Carballeiras, Campus do Elviña, 16737 Universidade da Coruña, 15071 A Coruña, Spain; 4 Department of Medical Microbiology, Albert Szent-Györgyi Health Center and Albert Szent-Györgyi Medical School, 37442 University of Szeged, Semmelweis utca 6, 6725 Szeged, Hungary

## Abstract

Essential trace metals are vital for cellular processes, such as respiration, DNA replication, and photosynthesis. Cyanobacteria must tightly regulate metal homeostasis to prevent deficiency or toxicity, yet their metallophores remain overlooked. Here, we report lusichelins A–E (**1**–**5**), new metallophores isolated from the marine cyanobacterium *Lusitaniella coriacea* LEGE 07167. Their structures and configurational assignments were determined by using NMR, mass spectrometry, TD-DFT calculations, and retrobiosynthetic insights. Lusichelins feature a unique structural arrangement with thiazoline/thiazole rings connected via a vinyl group, an aliphatic carbon chain, or directly enabling the potential for metal coordination. Genomic analysis identified a hybrid PKS/NRPS biosynthetic gene cluster consistent with the lusichelin structure, bearing traits characteristic of metallophore biosynthesis. Functionally, lusichelins act as metallophores capable of chelating both iron and copper. Lusichelin C (**3**) consistently bound iron under both metal-rich and metal-limited culture conditions, while copper complexation was only observed under elevated copper levels. At physiologically relevant pH values, no significant metal-binding preference was detected. Moreover, compound production was maximized under metal-rich conditions and in response to copper limitation. Lusichelin B (**2**) exhibited cytotoxicity against colon carcinoma cells while reversing multidrug resistance via ABCB1 efflux pump modulation. These findings expand our understanding of cyanobacterial metallophores in microbial metal homeostasis and highlight their biological potential.

## Graphical abstract

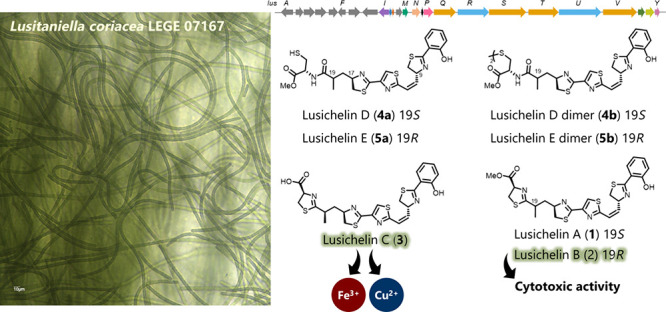

Cyanobacteria, ancient photosynthetic microorganisms, have thrived for over 3.5 billion years, inhabiting diverse ecosystems ranging from marine and freshwater habitats to extreme environments such as deserts, hot springs, and metal-contaminated areas.^1,2^ This remarkable ecophysiological diversity is mirrored in their metabolic plasticity, highlighting their value in drug discovery. Cyanobacteria have contributed to the development of four approved anticancer drugs, with numerous other secondary metabolites recognized for their unique structures and diverse bioactivities.^3^ As photoautotrophs, cyanobacteria have a particularly high demand for metals, as these micronutrients serve as cofactors in numerous proteins involved in respiration and photosynthesis. However, excess metals can disrupt these critical processes, necessitating sophisticated mechanisms to maintain metal homeostasis. In cyanobacteria, these include producing extracellular polymeric substances, synthesizing metallothioneins, activating metal transporters, and excreting metallophores.^2,4,5^

Metallophores are small molecules that bind metal ions to facilitate their uptake by cells through specific receptors or to detoxify excess metals via extracellular chelation.^6^ They are typically named according to their metal-binding preference, such as siderophores (iron), chalkophores (copper), and zincophores (zinc).^6^ Among these, siderophores make up the most extensively studied class of microbial metallophores. Their production is generally influenced by the iron requirements of the organism and the availability of iron in the local environment.^7^ Based on their chemical structures, these iron chelators can be classified as hydroxamates, catecholates/phenolates, α-hydroxy carboxylates, or mixed type.^7–9^ Knowledge of cyanobacterial metallophores remains limited. Reported examples have largely been isolated from iron-limited cultures and belong either to the hydroxamates, such as schizokinen and synechobactin A, or to the catecholates, including anachelin H and cyanochelin A.^10–13^ More recently, leptochelins were shown to bind promiscuously to zinc, copper, iron, and cobalt.^14^ Fatuamide A demonstrated a preferential affinity for copper over zinc and iron, and its producing strain exhibited high resistance to toxicity from elevated copper concentrations in the culture medium.^15^

Recent advances in genome mining tools allow for the detection of signature genes responsible for specific metallophore biosynthesis, such as those from nonribosomal peptide synthetases (NRPSs) or NRPS-independent synthetases.^16^ These genes are often found in biosynthetic gene clusters (BGCs) alongside metallophore transport and regulatory genes, such as TonB transporters and AraC transcriptional regulators.^17,18^ Given the biosynthetic richness of cyanobacteria and the growing power of bioinformatics tools, the discovery of novel cyanobacterial metallophores is likely to be brought to light in the near future.

During our bioactivity-guided discovery program, using untargeted metabolomics, we identified the filamentous marine cyanobacterium *Lusitaniella coriacea* LEGE 07167 as a potential producer of novel cytotoxic compounds.^19^ This discovery prompted a deeper investigation into this strain, leading to the characterization of lusichelins A–E (**1**–**5;** Figure 1), a new group of cyanobacterial metallophores. The putative BGC identified in this strain includes transport and regulator gene signatures consistent with metallophore biosynthesis and compatible with the proposed structures. Our experiments suggested a potential ecological role for lusichelin C (**3**) as both an iron and copper chelator. We hypothesize that lusichelin C (**3**) serves as a primary metallophore, with lusichelins A (**1**) and B (**2**), carboxylate forms of **3**, serving as cellular reservoirs. Furthermore, we confirmed the previously observed bioactivity,^19^ with lusichelin B (**2**) demonstrating stereospecific cytotoxic activity against human colon cancer cells and modulation of efflux pumps related to cancer multidrug resistance. Our study not only increases the known diversity of cyanobacterial metallophores but also provides insights into their multifunctional roles in metal homeostasis and cytotoxicity.

**Figure 1:**
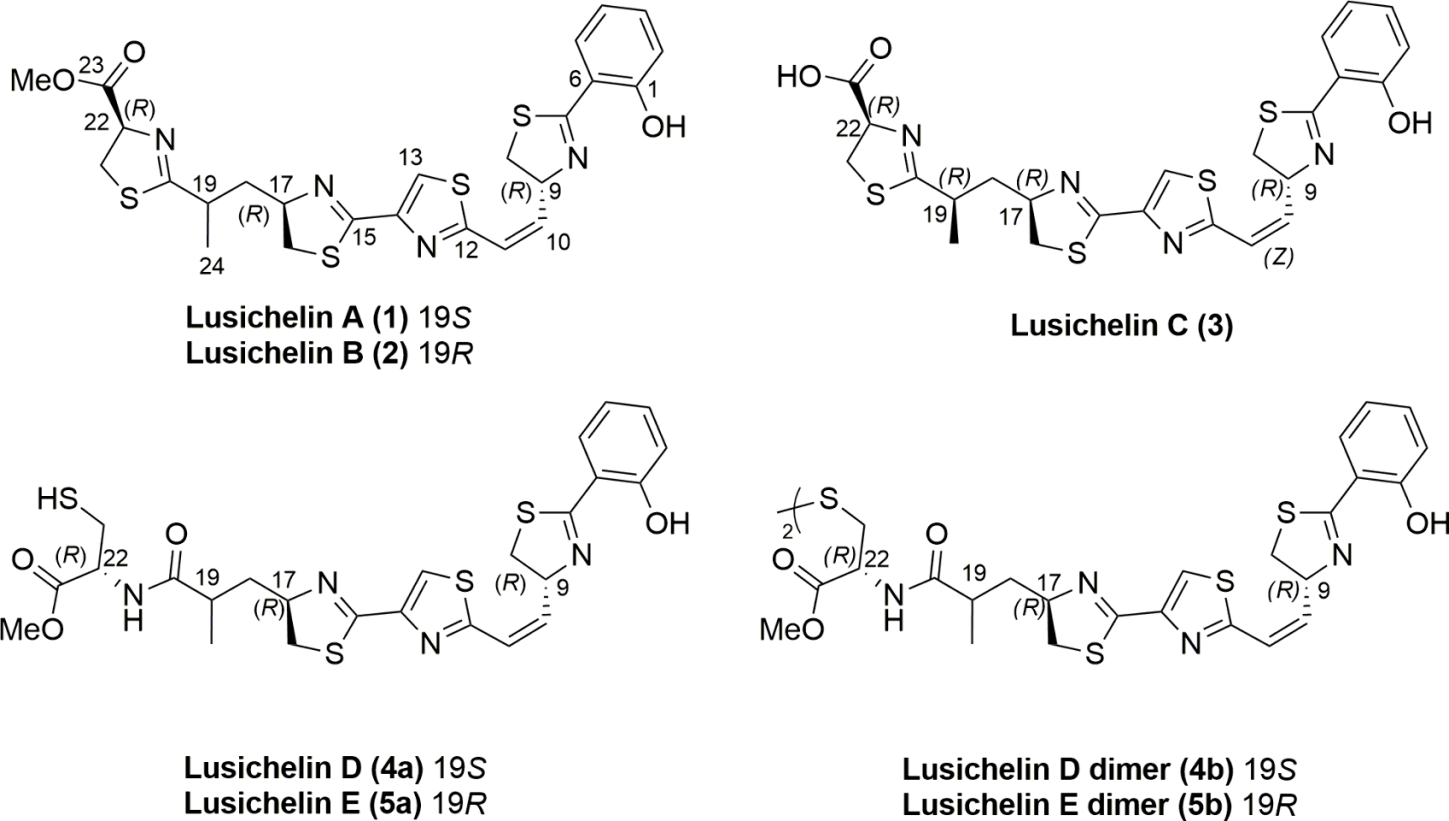
Structures of lusichelins A–E (**1**–**5**) isolated from *Lusitaniella coriacea* LEGE 0716.

## Results and Discussion

### Isolation and Structure Elucidation of the Planar Structure of Lusichelins A–D (**1**–**5**)

In our previous bioprospecting project involving several cyanobacteria from the LEGE-CC collection,^19^ we identified *Lusitaniella coriacea* LEGE 07167 as a potential producer of novel secondary metabolites. Through molecular network analysis using the GNPS (Global Natural Products Social Molecular Networking) platform,^20^ we discovered a unique cluster in this strain, composed of five mass features that could not be de-replicated. After culture scale-up of the producing cyanobacterium, mass-guided isolation (SI Figure S1), involving several normal and reverse-phase chromatographic steps, yielded lusichelins A–E (**1**–**5,** Figure 1).

The molecular formula of **1**, ([α]_D_^23^ +202.2), C_25_H_26_N_4_O_3_S_4_, was deduced from the [M + H]^+^ protonated molecule observed in its (+)-HRESIMS spectrum at *m*/*z* 559.0964, (calcd for C_25_H_27_N_4_O_3_S_4_, *m*/*z* 559.0961), indicating the presence of 15 degrees of unsaturation. Strong infrared absorption bands at 3400 and 1742 cm^–1^ suggested the presence of hydroxy and ester carbonyl functionalities, respectively.

The ^1^H and ^13^C NMR data of **1** (Table 1), combined through HSQC-edited experiments, allowed the identification of 25 carbon resonances, which were assigned to two methyl groups, four methylenes, four aliphatic and seven olefinic methines, and eight sp^2^ nonprotonated carbons. The presence of three thiazoline rings in **1** was inferred from an HMBC experiment.

**Table 1:** NMR Spectroscopic Data (δ in ppm) of Compounds **1**–**3** in CDCl_3_

	Lusichelin A (**1**)^a^		Lusichelin B (**2**)^a^		Lusichelin C (**3**)^b^	
position	δ_C_, type	δ_H_ (*J* in Hz)	δ_C_, type	δ_H_ (*J* in Hz)	δ_C_, type	δ_H_ (*J* in Hz)
**1**	159.2, C		159.4, C		169.9, C	
**2**	117.2, CH	7.00, dd (8.5, 1.2)	117.3, CH	7.00, dd (8.5, 1.2)	123.7, CH	6.67, dd (8.6, 1.1)
**3**	133.2, CH	7.36, ddd (8.5, 7.3, 1.6)	133.2, CH	7.36, ddd (8.5, 7.3, 1.6)	134.4, CH	7.20, ddd (8.6, 6.8, 1.8)
**4**	119.0, CH	6.89, ddd (7.3, 7.7, 1.2)	119.0, CH	6.89, ddd (7.3, 7.7, 1.2)	113.6, CH	6.48, td (7.4, 6.8, 1.1)
**5**	130.8, CH	7.47 dd, (7.7, 1.6)	130.8, CH	7.47, dd (7.7, 1.6)	133.4	7.44, dd (8.0, 1.8)
**6**	116.5, C		116.6, C		117.6, C	
**7**	172.8, C		172.8, C		176.3, C	
**8 a**	37.2, CH_2_	4.14, dd (10.9, 8.6)	37.1, CH_2_	4.13, dd (11.0, 8.4)	38.3, CH_2_	3.57, dd (11.5, 6.6)
**8 b**		3.19, dd^c^ (10.9, 8.6)		3.19, dd (11.0, 8.4)		3.26, d (11.5)
**9**	74.9, CH	6.32, q (8.2)	74.9, CH	6.32, qd (8.2, 1.2)	69.9, CH	5.91, ddd (6.6, 4.5, 2.1)
**10**	138.0, CH	6.25, dd (11.3, 8.0)	138.0, CH	6.25, dd (11.2, 8.2)	139.3, CH	6.30, dd (12.3, 4.5)
**11**	121.1, CH	6.62, dd (11.3, 1.4)	121.1, CH	6.63, dd (11.2, 1.2)	121.7, CH	6.53, dd (12.3, 2.1)
**12**	163.2, C		163.2, C		166.7, C	
**13**	119.0, CH	7.97, s	119.0, CH	7.96, s	124.7, CH	7.78, s
**14**	150.5, C		150.5^c^, C		147.6, C	
**15**	161.8, C		162.1, C		167.3, C	
**16 a**	38.2, CH_2_	3.48, m^c^	37.9, CH_2_	3.50, dd (10.9, 8.4)^c^	39.2, CH_2_	3.70, dd (10.9, 8.5)
**16 b**		3.03, ddd (11.0, 8.3, 3.2)		3.07, dd (10.9, 8.1)		3.23, t (10.9)
**17**	76.1, CH	4.72, qd (8.3, 6.0)	75.4, CH	4.70, p (7.8)	72.9, CH	4.32, tdd (12.3, 8.5, 4.9)
**18 a**	41.3, CH_2_	2.08, ddd (14.0, 8.6, 6.0)	40.7, CH_2_	2.32, dt (13.7, 7.5)	41.0, CH_2_	2.19, ddd (13.5, 11.7, 6.3)
**18 b**		2.00, ddd (14.0, 8.1, 5.8)		1.81, dt (13.7, 7.2)		1.95, ddd (13.5, 11.6, 4.9)
**19**	37.7, CH	3.22, m^c^	37.1, CH	3.14, q (7.0)	35.0, CH	4.58, dt (11.7, 6.6)
**20**	179.6, C		179.7, C		181.8, C	
**21 a**	35.1, CH_2_	3.55, m^c^	35.1, CH_2_	3.56, m^c^	33.6, CH_2_	3.97, dd (11.3, 6.2)
**21 b**		3.52, m^c^		3.49, m^c^		3.49, t (11.3)
**22**	77.9, CH	5.11, bt (9.1)	77.8, CH	5.09, bt (9.0)	76.2, CH	4.82, dd (11.3, 6.2)
**23**	171.5, C		171.5, C		174.5, C	
**24**	20.3, CH_3_	1.32, d (7.0)	19.6, CH_3_	1.36, d (6.9)	19.9, CH_3_	1.17, d (6.6)
**25**	52.9, CH_3_	3.80, s	52.9, CH_3_	3.80, s		
**1-OH**		12.63, bs		12.59, bs		
**20-NH**						

a ^1^H and ^13^C NMR spectra recorded at 600 and 151 MHz, respectively.

b ^1^H and ^13^C NMR spectra recorded at 400 and 101 MHz, respectively.

c Overlapped signals

Three sets of long-range correlations were observed, which were assigned to the presence of the respective thiazoline moieties (**A**, **C**, and **D** rings in Figure 2A): (1) from δ_H_ 4.14/3.19 (H-8a/H-8b) and 6.32 (H-9) to δ_C_ 172.8 (C-7); (2) from δ_H_ 3.48/3.03 (H-16a/H-16b) and 4.72 (H-17) to δ_C_ 161.8 (C-15); and (3) from δ_H_ 3.55/3.52 (H-21a/H-21b) and 5.11 (H-22) to δ_C_ 179.6 (C-20). The presence of a thiazole ring (**B** ring in Figure 2A) was also deduced from the HMBC cross-peaks between the nonprotonated sp^2^ carbons at δ_C_ 163.2 (C-12) and 150.5 (C-14) and the sp^2^ methine proton at δ_H_ 7.97 (s, H-13). Furthermore, the ^1^H-^1^H COSY experiment revealed four isolated spin systems that were connected by heteronuclear ^1^H-^13^C HMBC correlations (Figure 2A).

**Figure 2:**
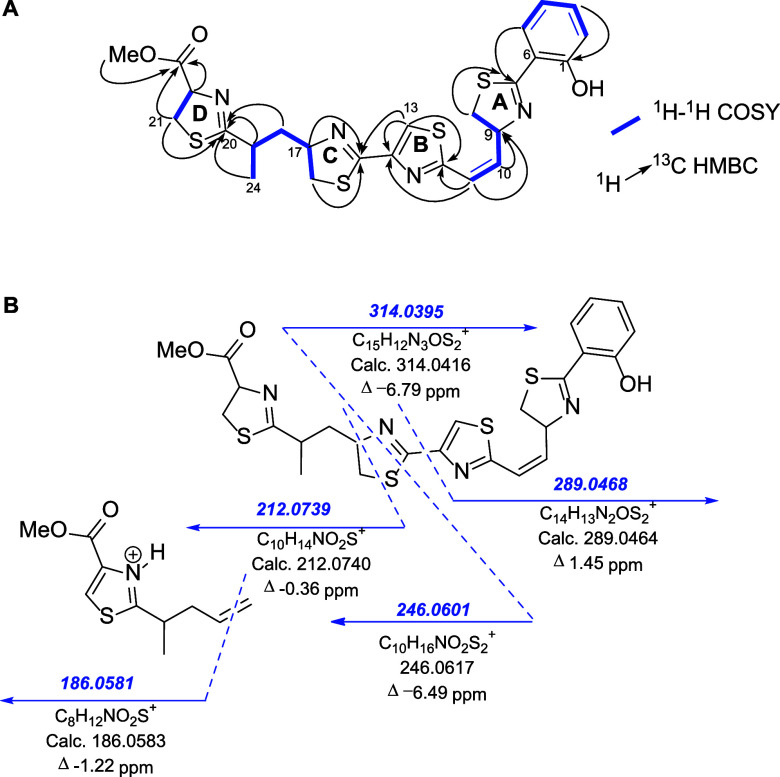
Structure determination of lusichelins A and B (**1** and **2**, respectively). (A) Four independent ^1^H-^1^H spin systems (highlighted in bold blue) deduced from their ^1^H-^1^H COSY spectra were connected by key HMBC correlations (indicated by black arrows). (B) MS^2^ fragmentation pattern with major ions annotated (*m*/*z*), deduced from (+)-HRESIMS/MS analysis.

The ^1^H NMR spectrum of **1** displayed characteristic signals of four aromatic protons at δ_H_ 7.47–6.89, whose splitting pattern and coupling constants (*ortho J* = 7.3 and 8.5 Hz, *meta J* = 1.2 and 1.6 Hz) were indicative of an *ortho*-disubstituted benzene ring. The broad singlet at δ_H_ 12.63 observed in the ^1^H NMR spectrum of **1** suggested the presence of an *ortho*-substituted phenol ring. This *ortho*-phenol moiety was connected to the first thiazoline (**A** ring) through a long-range correlation between the imine carbon at δ_C_ 172.8 (C-7) to the aromatic proton H-5 (δ_H_ 7.47 dd), suggesting the presence of a salicyl-thiazoline moiety. This assignment was further confirmed by comparison with compounds such as piscibactin^21^ and amamistatin,^22^ bearing a similar fragment. Key ^1^H-^1^H COSY correlations were observed between methylene protons at δ_H_ 4.14 and 3.19 (H-8a/H-8b) and the methine proton at δ_H_ 6.32 (H-9) of the thiazoline **A** ring, along with the olefinic protons H-10 (δ_H_ 6.25) and H-11 (δ_H_ 6.62). Additionally, an HMBC correlation was observed between the olefinic proton H-11 and the thiazole carbon at C-12 (δ 163.2). These ^1^H-^1^H COSY and HMBC correlations established the connectivity between thiazoline **A** and thiazole **B** rings through the Δ^10^ olefin linkage. Likewise, the ^1^H-^1^H spin system from methylene H-16 to the methyl H-24 and the long-range proton-carbon correlation from H-19 (δ_H_ 3.22), H-21 (δ_H_ 3.55/3.52), and H-24 (δ_H_ 1.32) to C-20 (δ_C_ 179.6) allowed us to connect the second to the third thiazoline (**C** and **D** rings, respectively) through a CH­(CH_3_)­CH_2_ group (Figure 2A). Thiazole **B** and thiazoline **C** rings were connected through C-14 and C-15 carbons based on an HMBC correlation from H-13 (δ_H_, s, 7.97) to C-15 (δ_C_ 161.8). Finally, a methoxycarbonyl group (δ_H_ 3.80 s; δ_C_ 52.9 (C-25) and 171.5 (C-23)) was linked to position C-22 of the thiazoline **D** ring by the HMBC correlation between C-23 (δ_C_ 171.5) and H-22 (δ_H_ 5.11). The 1D and 2D NMR data (SI Figures S2–S6) allowed us to establish the planar structure of **1**, named as lusichelin A, which was also supported by the mass fragmentation pattern displayed in its (+)-HRESIMS/MS spectrum (Figure 2B).

The (+)-HRESIMS spectrum of **2** ([α]_D_^23^ +152.4) displayed a [M + H]^+^ ion at *m*/*z* 559.0964, indicating that it is an isomer of **1**. Furthermore, NMR data of **2**, named lusichelin B, were very similar to those of **1** (Table 1; SI Figures S10–S14), except for the chemical shifts at positions C-17, C-18, C-19, and C-24 (Δδ_C_ > 0.5 ppm). These differences suggested a different configuration at either C-17 or C-19 between **1** and **2** (see below).

Compound **3**, named as lusichelin C, ([α]_D_^24^ +431.3; yellow amorphous powder) showed a prominent [M + H]^+^ ion in its (+)-HRESIMS spectrum at *m*/*z* 545.0803 (calcd. for C_24_H_25_N_4_O_3_S_4_ *m*/*z* 545.0804), indicating a molecular formula of C_24_H_24_N_4_O_3_S_4_. Given the similarity of the ^1^H and ^13^C NMR data for **3** to those of **1** and **2**, an analogous assignment strategy was used to elucidate its structure (Table 1; SI Figures S17–S23). The mass difference of 14.0157 Da between the [M + H]^+^ ion of **3** relative to that of **1** or **2** suggested the absence of a methyl group in **3**. This was confirmed by the lack of proton and carbon chemical shifts in the NMR spectra of **3** corresponding to a methoxy group present in **1** and **2**. All of these data indicated the presence of a carboxylic acid functionality in **3** (δ_C‑23_ 174.5) instead of the methyl ester group present in **1** and **2** (Table 1). Compound **3** was also isolated from the biomass of *L. coriacea* LEGE 07167 as a complex with iron (**3-Fe**, *m*/*z* [M + Fe – 2H]^+^ = 597.9919; Δ = 52.9116) as a red amorphous powder (SI Figures S30–31).

The molecular formula of **4a** was established as C_25_H_28_N_4_O_4_S_4_ on the basis of its (+)-HRESIMS spectrum, which displays the [M + H]^+^ ion at *m*/*z* 577.1069 (calcd for C_25_H_29_N_4_O_4_S_4_ *m*/*z* 577.1066). Comparison of the molecular formula of **4a** to that of **1** and **2** indicated that **4a** has an additional oxygen and two additional hydrogen atoms (Δ = 18.0105 Da). The ^1^H and ^13^C NMR data of **4a** from position C-1 to C-19 (Table 2) were identical to those of **1** and **2**, suggesting that **4a** has the same salicyl-thiazoline fragment connected through a disubstituted (*Z*)-olefin to a thiazole-thiazoline moiety and this is in turn to a −CH­(CH_3_)­CH_2_– group. The main carbon and proton chemical shift differences between the NMR spectra of **1**/**2** and **4a** were observed at positions C-21 and C-22. The observed differences, along with the presence of additional OH_2_ atoms, suggested that thiazoline ring **D** in **4a** might be opened. This proposal was supported by the spin system observed in the ^1^H-^1^H COSY spectrum of **4a** displaying correlations among the amide proton at δ_H_ 6.96 (d, *J* = 7.8 Hz), the methine H-22 (δ_H_ 4.95 dt/ δ_C_ 53.7), and the methylene H-21 (δ_H_ 3.07 and 2.99/ δ_C_ 27.2). The characteristic ^1^H and ^13^C NMR chemical shift values of α- and β-carbons of a methylester cysteine moiety for C-22 and C-21 (Table 2) and those corresponding to a methoxy group at δ_C_ 53.0/δ_H_ 3.80 agree with the presence of an opened thiazoline ring **D** bearing a methylester in **4a**. Moreover, the HMBC cross-coupling from H-21 to the carbonyl group at C-20 (δ_C_ 175.7) allowed us to link the methylester cysteine moiety to the C-1–C-19 fragment through an amide functionality. The 14 degrees of unsaturation present in **4a,** instead of the 15 degrees in **1** and **2,** agrees with the presence of an opened thiazoline ring in **4a**. Interestingly, the NMR spectra of **4a** displayed additional chemical shifts that suggested that it was isolated along with its sulfide dimer, compound **4b** (SI Figures S32–S36) The presence of **4b** was deduced by the sequential proton–proton cross peaks of an amide proton at δ_H_ 7.04 (d, *J* = 7.8 Hz) with a methine H-22 (δ_H_ 4.86/ δ_C_ 51.6) and methylene H-21 (δ_H_ 3.18/δ_C_ 40.3) observed in a ^1^H-^1^H COSY experiment. LC/HRESIMS analysis confirmed the presence of sulfide dimer **4b** (SI Figure S37). Indeed, the HPLC chromatogram displayed **4a** at *t*_R_ 4.33 min along with another chromatographic peak at *t*_R_ 13.88 min corresponding to **4b**, for which (+)-HRESIMS showed a [M + Na]^+^ ion at *m*/*z* 1173.1721 (calcd *m*/*z* 1173.1722 for C_50_H_54_NaN_8_O_8_S_8_) and a doubly charged ion at *m*/*z* 576.0986 [M + H]^2+^. A 1:0.8 ratio between monomer **4a** and sulfide dimer **4b** was deduced from the ^1^H NMR spectrum. Compounds **4a** and **4b** were named lusichelin D and lusichelin D dimer, respectively. The isomeric structure between **5a** and **4a** was proposed from the [M + H]^+^ ion displayed at *m*/*z* 577.1069 in the (+)-HRESIMS spectrum of **5a**. Also, the NMR spectral data of **5a** were very similar to those of **4a** (Table 2) but differed in the chemical shifts at positions C-17 and C-19, suggesting a different configuration at these stereocenters. The presence of a mixture of the thiol monomer **5a** and its sulfide dimer **5b**, which were called lusichelin E and lusichelin E dimer, respectively, was also deduced from the NMR (SI Figures S38–S42) and LC/(+)-HRESIMS data (SI Figure S43). In this case, the ^1^H NMR spectrum displayed a 0.6:1 ratio between thiol **5a** and its sulfide dimer **5b**. The origin of **4b** and **5b** as extraction or separation artifacts could not be ruled out.

**Table 2:** NMR Spectroscopic Data (δ in ppm) of Compounds **4a** and **5a** in CDCl_3_ ^a^

	Lusichelin D (**4a**)		Lusichelin E (**5a**)	
position	δ_C_, type	δ_H_ (*J* in Hz)	δ_C_, type	δ_H_ (*J* in Hz)
**1**	159.3, C		159.3, C	
**2**	117.2, CH	7.00, dd (8.3, 1.1)	117.3, CH	7.00, bd (8.0)
**3**	133.2, CH	7.36, m	133.3, CH	7.36, t (8.0)
**4**	119.0, CH	6.89, tdd (7.7, 4.2, 1.1)	119.0, CH	6.89, td (8.0, 2.8)
**5**	130.8, CH	7.47, dd (7.7, 1.7)	130.8, CH	7.46, d (8.0)
**6**	116.5, C		116.5, C	
**7**	172.8, C		172.8, C	
**8 a**	37.2, CH_2_	4.13, dd (10.9, 8.5)	37.2, CH_2_	4.14, q (9.1)
**8 b**		3.20, m		3.19, m^b^
**9**	74.8, CH	6.32, bq (8.5)	74.9, CH	6.30, q (8.4)
**10**	138.1, CH	6.25, m	138.0, CH	6.25, dt (11.3, 7.8)
**11**	121.0, CH	6.62, td (11.5, 1.4)	121.1, CH	6.62, bd (11.3)
**12**	163.4, C		163.2, C	
**13**	120.3, CH	8.02, s	120.0, CH	7.99, s
**14**	150.3, C		150.5^b^, C	
**15**	162.8, C		162.0^b^,C	
**16 a**	38.4, CH_2_	3.49, dd (8.5, 2.7)	38.1, CH_2_	3.52, bt (9.6)
**16 b**		3.00, m		3.03, m^b^
**17**	75.7, CH	4.66, ddt (13.0, 8.3, 4.7)	76.0, CH	4.69, ddt (12.2, 9.1, 3.5)
**18 a**	40.3, CH_2_	2.03, ddd (13.3, 10.6, 4.6)	39.5, CH_2_	2.22, m
**18 b**		1.81, ddd (13.3, 10.6, 4.4)		1.79, m
**19**	38.9, CH	2.84, m	38.9, CH	2.74, q (7.2)
**20**	175.7, C		176.2, C	
**21 a**	27.2, CH_2_	3.07, ddd (14.2, 8.1, 4.2)	26.2, CH_2_	3.00, m
**21 b**		2.99, m		
**22**	53.7, CH	4.95, dt (8.1, 4.2)	53.1, CH	4.87, m
**23**	170.9, C		171.1, C	
**24**	18.3, CH_3_	1.22, d (6.8)	18.0, CH_3_	1.30, d (7.1)
**25**	53.0, CH_3_	3.80, s	53.0, CH_3_	3.76, s
**1-OH**		12.62, bs		12.63, bs
**20-NH**		6.96, d (7.8)		6.64, d (7.4)

a ^1^H and ^13^C NMR spectra recorded at 600 and 151 MHz, respectively.

b Overlapped signals

### Identification of the Putative Lusichelin (*lus*) Biosynthetic Gene Cluster

To identify the putative lusichelin biosynthetic gene cluster (BGC), the whole genome of *L. coriacea* LEGE 07167 was sequenced. A combination of short-read Illumina and long-read Nanopore sequencing technologies yielded a draft genome of 5.97 Mb as two contigs (Genbank: JBBBDN000000000). The sequence was analyzed using antiSMASH 7.0,^18^ which identified seven BGCs. The only plausible candidate for lusichelin biosynthesis was annotated as a hybrid NRPS/PKS metallophore BGC, encoding four NRPS modules intercalated by type I PKS genes (Figure 3A). The four NRPS modules contain adenylation domains with predicted specificity for cysteine as well as heterocyclization domains suggesting the presence of thiazoline/thiazole moieties in the BGC products, which is consistent with the structure of lusichelins A–C (**1**–**3**). Other significant features encoded within this BGC include a salicylate synthase, metallophore-related transporters, and an AraC transcriptional regulator (SI Table S1). The ATP-binding cassette (ABC) and major facilitator (MFS) superfamilies of transporters, TonB-dependent receptor, and heavy metal translocating P-type ATPase are related to cyanobacterial siderophore export/import systems.^4^ Based on these findings, we consider this BGC as the putative lusichelin gene cluster (*lus*) and propose a biosynthetic model for the assembly of lusichelins (Figure 3B).

**Figure 3:**
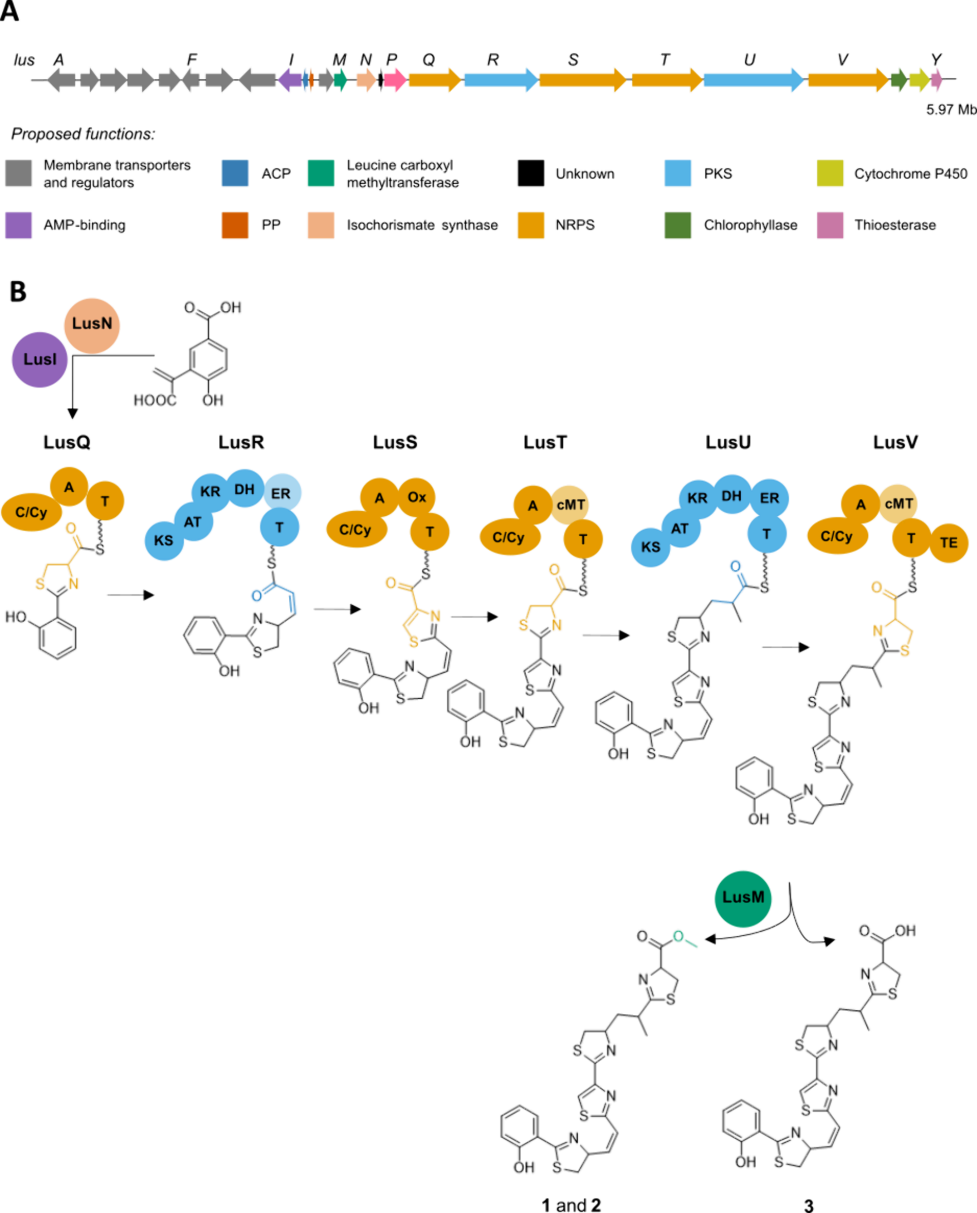
Proposed biosynthetic pathway of lusichelins in *Lusitaniella coriacea* LEGE 07167. (A) Bioinformatics-based annotation of *lus* BGC. ACP (acyl-carrier protein); PKS (polyketide synthase); PP (phospho­pantetheinyl transferase); NRPS (non-ribosomal peptide synthetase). (B) Biosynthetic model for the assembly of lusichelins A–C (**1**–**3**) along with domain annotation. C/Cy (condensation/heterocyclization); A (adenylation); T (thiolation); KS (ketosynthase); AT (acyltransferase); KR (ketoreductase); DH (dehydratase); ER (enoylreductase); Ox (oxidase); cMT (carbon methyltransferase); TE (thioesterase).

The assembly line for lusichelins mirrors other salicyl-capped siderophore biosynthetic pathways.^8^ It is proposed to initiate with the generation and activation of salicylate: LusN, a putative bifunctional salicylate synthase, converts chorismate directly to salicylate, which is then activated by LusI, a putative AMP ligase (Figure 3B). The activated salicylate then undergoes condensation with cysteine, catalyzed by the condensation/heterocyclization domain of the first NRPS module, LusQ, to form the initial thiazoline ring (**A** ring). This process is reminiscent of the biosynthesis observed in bacterial siderophores such as yersiniabactin or piscibactin.^21,23^ The ensuing PKS module—LusR—generates the olefin linkage by adding a malonate unit to the hydroxyphenyl-thiazoline, followed by reduction and dehydration through its KR and DH domains. The following NRPS modules LusS and LusT contain domains for the incorporation of cysteine and heterocycle formation, respectively. LusS also features an oxidation domain, consistent with the formation of the thiazole moiety (**B** ring), analogous to other thiazole-containing cyanobacterial natural products like aranazoles and hectochlorin.^24,25^ LusT, in addition to thiazoline formation (**C** ring), contains an SAM-dependent methyltransferase domain (cMT). LusU then carries out a PKS elongation, where malonyl is predicted as the substrate (the AT domain contains the malonyl-specific active site residue motifs GHSVG and HAFH), which is then fully reduced by its KR-DH-ER domains. This process results in the (methyl)­ethyl linkage observed in the structures of lusichelins. However, bioinformatics analysis did not identify a methyltransferase domain within LusU, leaving the origin of the methyl group at C-19 unclear. The final NRPS module, LusV, contains domains for the formation of the third thiazoline ring (**D** ring) and includes both cMT and type I thioesterase domains. LusY encodes a putative type II thioesterase as a standalone protein. Type I thioesterases typically release final products in NRPS/PKS biosynthesis, while type II thioesterases, when co-occurrent in the BGC, are often considered to have a “proofreading” role in the biosynthesis pathway.^26,27^ This leads us to propose that LusV terminates the biosynthesis, resulting in the release of lusichelin C (**3**) (Figure 3B). Moreover, compounds **1**, **2**, **4a**, and **5a** contain a terminal methyl ester. The formation of the methyl ester **1** and **2** as artifacts of **3** during methanolic extraction was ruled out, as these compounds were also the major components when the extraction was performed with dichloromethane (SI Figures S44 and S45). We propose that this terminal methylation could be catalyzed by LusM, a putative SAM-dependent methyltransferase with a leucine carboxyl methyltransferase (LCM) domain. Similar enzymatic functions have been reported in bacterial proteins such as StnF2 and MelK, which catalyze methyl esterification in the biosynthesis of streptonigrin and melithiazol, respectively.^28,29^

### Configuration Analysis of Lusichelins

Due to limited compound availability, stereochemical studies were conducted exclusively with lusichelin C (**3**), the configuration of which was determined through a combination of *J*-based configurational analysis (JBCA), electronic circular dichroism (ECD), TD-DFT calculations, Marfey’s analysis, and insights from the putative *lus* BGC (Figure 4A).

**Figure 4:**
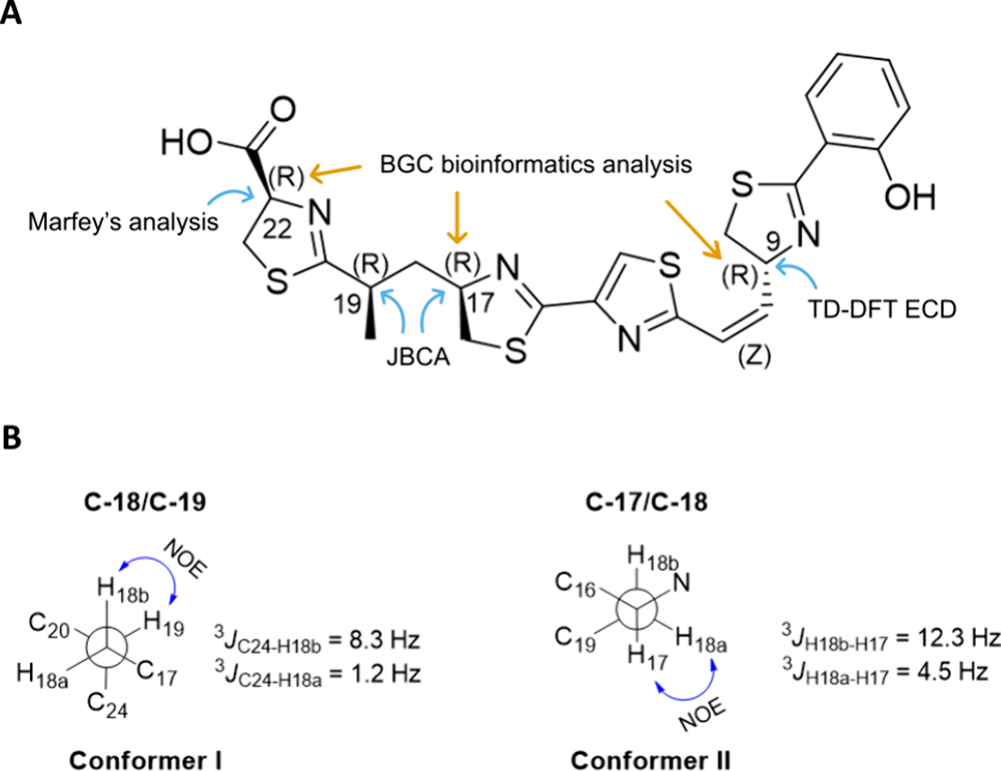
(A) Configuration determination approach for lusichelin C (**3**). (B) Newman projections of conformers **I** and **II** of **3** around the C-18–C-19 and C-17–C-18 bonds, respectively, along with key experimental ^3^ *J*_CH_ and ^3^ *J*_HH_ coupling constants and NOESY correlations around these positions.

The *Z* configuration of the Δ^10^ double bond of **3** was deduced from the characteristic *J*_H10‑H11_ value of 12.3 Hz (Table 1). The relative configuration between the C-17 and C-19 stereogenic centers in **3** was determined by using the JBCA methodology. First, ^1^H-^13^C heteronuclear coupling constant values were obtained via an HSQC-HECADE experiment (SI Figure S24). Then, two rotamers around the C-18/C-19 bond were deduced from the *antiperiplanar* and *gauche* orientations between H-18b/C-24 and H-18a/C-24, suggested by the large and small heteronuclear coupling constants values of ^3^ *J*_H‑18b‑C‑24_ (8.3 Hz) and ^3^ *J*_H‑18a‑C‑24_ (1.2 Hz), respectively. Finally, conformer **I** around the C-18/C-19 bond displayed in Figure 4B was selected based on the NOESY correlation between H-18b and H-19. In the same way, the conformer **II** around the C-17/C-18 bond showed in Figure 4B was deduced from the large and a small proton–proton coupling constant of 12.3 and 4.5 Hz between H-18b/H-17 and H-18a/H-17, indicating *antiperiplanar* and *gauche* dispositions for these protons, respectively, along with NOESY correlation between H-18a and H-17. Once conformers **I** and **II** were deduced, a 17R*,19R* configuration was proposed. This configuration between C-17 and C-19 agrees with the Δδ_H_ value of 0.24 between chemical shifts of the two methylene diasterotopic protons at C-18 following the geminal proton rule reported by Schmidt and Breit.^28^

The absolute configuration at C-22 in **3** was determined using Marfey’s analysis.^30^ A careful derivatization of **3** was crucial to avoid the epimerization at the C_α_ of the thiazoline ring under acidic or basic conditions.^31^ For this reason, **3** was hydrolyzed in 1.5 M aqueous HCl at 90 °C for 4 h, and the resulting hydrolysate was derivatized with l-FDAA and analyzed by lC-MS. The presence of l-Cys in **3** was deduced from the comparison of the retention times and *m*/*z* values of the former hydrolysate derivative, analyzed by LC/MS, to those of l- and d-cysteine standards derivatized with l-FDAA. Specifically, the hydrolysate derivative of **3** exhibited a retention time (*t*_R_) of 24.7 min and a *m*/*z* of 375, matching those obtained for the derivatized l-Cys standard (*t*_R_ of 25.03 min and *m*/*z* 375). In contrast, while the derivatized d-Cys standard displayed *m*/*z* 375, its retention time (21.6 min) was clearly different (SI Figure S26). Although the relative configuration between C-17 and C-19 and the absolute configuration at C-22 could be determined in **3**, the absolute configuration of the stereogenic center at C-9 could not be established. For this reason, four possible configurations (9*R*,17*R*,19*R*,22*R*, 9*S*,17*S*,19*S*,22*R*, 9*R*,17*S*,19*S*,22*R*, and 9*S*,17*R*,19*R*,22*R*) were considered, and their corresponding ECD spectra were calculated using time-dependent density functional theory (TD-DFT) at the CAM-B3LYP/6-311++G­(2d,p) level, considering 50 electronic states. Comparison of the experimental ECD spectrum of **3** to those of the calculated ones ruled out two of them, 9*S*,17*S*,19*S*,22*R* and 9*S*,17*R*,19*R*,22*R* (Figure 5). In contrast, the calculated DFT-ECD spectra for the remaining two (9*R*,17*S*,19*S*,22*R,* and 9*R*,17*R*,19*R*,22*R*) configurations matched very well to that of the experimental ECD spectrum of **3** (Figure 5). However, due to the high similarity of the ECD spectra, they cannot be distinguished; hence, either the 9*R*,17*S*,19*S*,22*R* or 9*R*,17*R*,19*R*,22*R* configuration is possible for **3** (Figure 5). Bioinformatics analysis of the adenylation domains in modules LusQ, LusT, and LusV predicted l-cysteine as their substrate (SI Table S2), implying *R* configurations at stereogenic centers C-9, C-17, and C-22. No epimerase domains were identified in LusQ, LusT and LusV, which would typically convert these centers to their d-configurations. Consequently, the 9*R,*17*R* configuration was suggested for **3** and so, the 9*R*,17*R*,19*R*,22*R* absolute configuration is proposed for this compound as shown in Figure 4.

**Figure 5:**
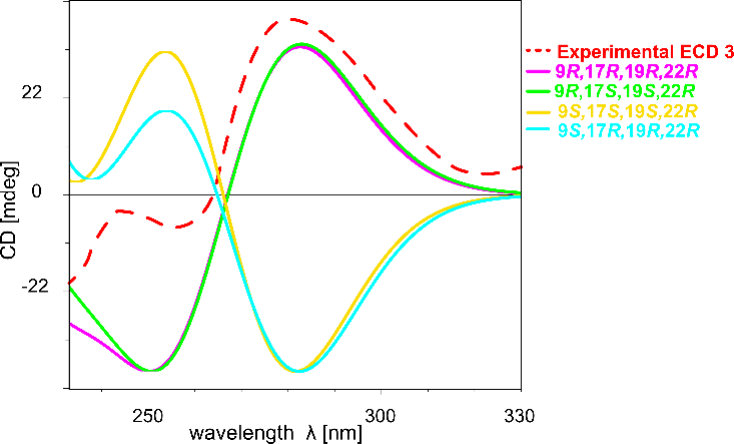
Comparison of the experimental ECD spectrum (dashed red line) of lusichelin C (**3**) to the calculated TD-DFT ECD spectra for 9*R*,17*R*,19*R*,22*R* (magenta line), 9*R*,17*S*,19*S*,22*R* (green line), 9*S*,17*S*,19*S*,22*R* (yellow line), and 9*S*,17*R*,19*R*,22*R* (cyan line).

Although the degradation of compounds **1**, **2**, **4a**/**4b**, and **5a**/**5b** prevented us from conducting a stereochemical study similar to that performed for **3**, a configuration proposal was suggested for these compounds. The stereogenic centers at C-9, C-17, and C-22 in **1**, **2**, **4a/4b**, and **5a/5b** share the same absolute configuration as that in **3** based on biogenetic considerations. The relative configuration at C-19 was deduced from the analysis of chemical shift differences (Δδ) of the diastereotopic H-18 methylene protons. The small Δδ_H_ values of 0.08 and 0.22 in **1** and **4a** are consistent with a 17R*,19S* relative configuration according to the geminal proton rule reported by Schmidt and Breit.^32^ Conversely, larger Δδ_H_ values of 0.51 and 0.43 in **2** and **5a** suggest a 17R*,19R* relative configuration. This pattern has also been observed in other natural products, including valactamides,^33^ verucopeptin,^34^ and polychlorinated leucine derivatives.^35,36^ Based on this configurational analysis, the absolute configuration of **2** and **5a/5b** must be identical to that of **3**, specifically 9*R*,17*R*,19*R*,22*R.* Therefore, the proposed absolute configuration of **1** and **4a/4b** would be 9*R*,17*R*,19*S*,22*R*.

### Influence of Iron Deprivation and Salt Type on Lusichelin Production

To investigate whether iron deficiency could regulate lusichelin production, cultures of *L. coriacea* LEGE 07167 were grown under iron-deprived conditions using two types of salt: synthetic Tropic Marin Pro-Reef (TM) sea salt (standard conditions) and ACS grade NaCl. The production of lusichelins A–C (**1–3**) was followed by LC-HRESIMS analysis of the culture media and crude biomass (Figure 6). It is worth noting that lusichelins A and B (**1** and **2**) were detected in higher concentrations in the cyanobacterial biomass compared to the culture media (Figure 6A). This is likely due to their lipophilic nature (as compounds **1** and **2** have a calculated logP of 4.66 compared to the logP of 2.59 of **3**). Moreover, the type of salt had a more significant impact on lusichelin production than iron limitation. Cultures grown in TM (with or without Fe) produced approximately twice as much **1**–**3** as those grown in NaCl (with or without Fe) (Figure 6A). These results suggest that iron limitation does not regulate the production of these compounds, which is atypical, as siderophore synthesis is usually upregulated under low-iron conditions.^37^ This was particularly intriguing, as we suspected that compound **3** functioned as a siderophore, evidenced by the isolation of its **3-Fe** complex from the biomass and its consistent detection under the tested experimental conditions shown in Figure 6B. It is worth noting that the *lus* BGC encodes a TonB-dependent receptor homologue (SI Table S1). In cyanobacteria, these protein receptors are involved in active iron transport and are associated with siderophore–iron complex uptake systems.^38^ Further investigation into the expression of transcriptional regulators and transporters encoded in the *lus* BGC may shed light on the regulatory mechanisms controlling the production of these compounds.

**Figure 6:**
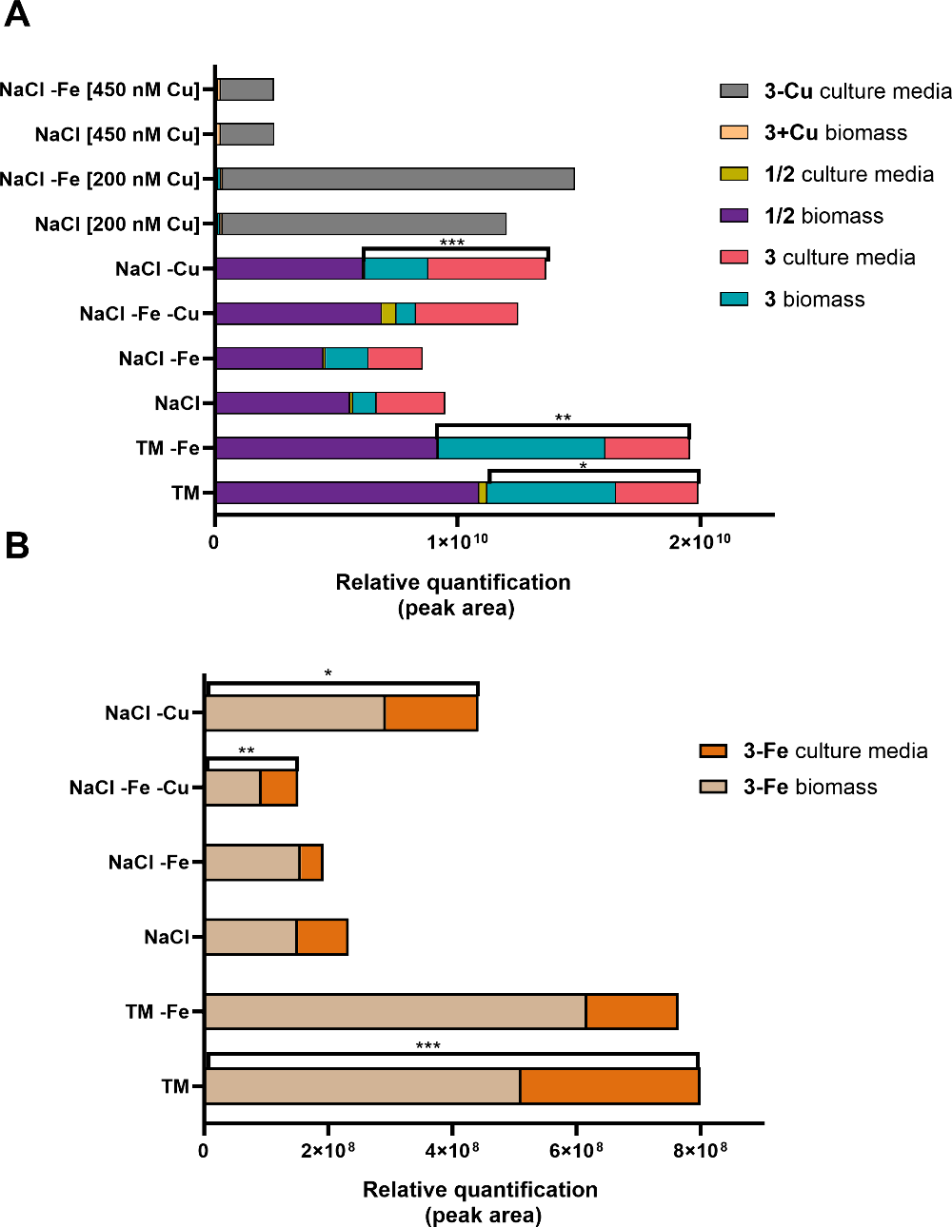
Lusichelin production in response to: different salt sources (TM; NaCl); iron limitation (TM -Fe; NaCl -Fe); copper limitation (NaCl -Cu), dual metal restriction (NaCl -Fe -Cu); high copper concentration (NaCl [200 nM Cu]; NaCl [450 nM Cu]) and high copper concentration with iron restriction (NaCl -Fe [200 nM Cu]; NaCl -Fe [450 nM Cu]). Relative quantification in biomass and culture media was determined by the peak area of the extracted ion chromatogram for (A) lusichelins A–C (**1**–**3**) and the copper–lusichelin C (**3**-**Cu**) complex and (B) the iron–lusichelin C (**3-Fe**) complex. The statistical differences (total amount of compound) of the different experimental conditions versus NaCl were tested using an unpaired *t* test (*p ≤ 0.05; **p ≤ 0.01; ***p ≤ 0.001; *n* = 3).

### Investigating Other Metallophore Roles of Lusichelins

Copper plays a crucial role in cyanobacterial homeostasis, yet it is also a significant contributor to marine environmental pollution, primarily due to anthropogenic activities such as the use of copper in antifouling paints for ship hulls and as an algicide.^5^ In a microcosm experiment, cyanobacteria were reported as the most copper-tolerant phylum among the prokaryotes found on marine periphytic biofilms exposed to environmentally relevant copper concentrations (0.01–10 μM).^39^

To explore the copper-chelating properties of lusichelins, cultures of *L. coriacea* LEGE 07167 were grown in Z8-NaCl media supplemented with elevated copper concentrations (200 and 450 nM CuSO_4_), which are 40–90× higher than those typically found in Z8 medium (5 nM CuSO_4_). Under these conditions, a new complex was detected in both the biomass and culture media, compatible with lusichelin C bound to copper (**3-Cu**), as evidenced by an [M + Cu – H]^+^ ion at *m*/*z* 605.9932 (C_24_H_23_CuN_4_O_3_S_4_; Δ = −2.82 ppm). A comparative analysis of lusichelin production in 200 nM CuSO_4_ versus NaCl (5 nM CuSO_4_) revealed an increase in the number of lusichelin copper complexes in the culture media. This led to a significant reduction in *apo*-lusichelins in the biomass and their complete absence from the culture media (Figure 6A). At 450 nM CuSO_4_, the abundance of **3-Cu** was significantly reduced (Figure 6A), likely due to copper competing with other metals for their binding sites in metalloproteins, resulting in toxicity, a phenomenon previously observed for other cyanobacteria.^2^

As copper and iron cooperate in maintaining cellular homeostasis, with some Cu-binding and Fe-binding proteins functioning interchangeably,^2,40^ we investigated how variations in the availability of both metals would affect lusichelin production. Therefore, we tested three additional conditions: copper limitation, dual restriction of iron and copper, and iron deprivation in the presence of excess copper. The absence of copper or the dual restriction of both metals had no significant effect on the production of lusichelins A and B (**1** and **2**). However, there was a significant increase in *apo*-lusichelin C (**3**) under copper deprivation, which was not observed for the dual restriction experiment (Figure 6A). The *holo*-lusichelin C **3-Cu** was not detected in any of these experimental conditions, while the *holo*-lusichelin C **3-Fe** was twice as abundant in the copper restriction experiment (Figure 6B). Cultures grown under iron restriction with excess copper (200 and 450 nM CuSO_4_) exhibited behavior similar to the experiments with copper excess alone, showing almost complete conversion of lusichelins to *holo*-lusichelin C **3-Cu**. The absence of iron did not significantly affect lusichelin production, and the iron complex was not detected in any of the copper-excess conditions. These results suggest that lusichelin C (**3**) may act as a chalkophore under high copper conditions, potentially contributing to copper detoxification.

### Iron and Copper Binding Preferences of Lusichelin C (**3**)

We investigated the metal-binding properties of *apo*-lusichelin C (**3**), focusing on its selectivity for iron versus copper across different pH conditions. First, we examined the binding preference of compound **3** for ferrous (Fe^2+^) versus ferric (Fe^3+^) iron. Equimolar amounts of **3** were incubated with FeCl_2_ or FeCl_3_ at pH 6.0, 7.5, and 8.5. The resulting UV–vis spectra (SI Figure S46) showed that ferric iron induced a greater increase in absorbance compared to ferrous iron, particularly at pH 6.0 and 8.5, suggesting preferential coordination of **3** with Fe^3+^. Next, we compared the binding of **3** to ferric (Fe^3+^) iron versus cupric (Cu^2+^) copper under the same concentrations and pH range (Figure 7). Iron and copper binding led to distinct absorbance spectra at all of the pH values tested. At the acidic pH, a stronger binding for Fe^3+^ was observed, and sequential addition experiments (Fe^3+^ → Cu^2+^ or Cu^2+^ → Fe^3+^) yielded identical spectra, which closely resembled those of the Fe^3+^ complex alone rather than the mixed-metal complex. At pH 7.5, Cu^2+^ binding was favored over that of Fe^3+^, but sequential or simultaneous addition resulted in minimal spectral differences. Under pH 8.5, which corresponds to the pH of the culture medium, stronger absorbance was recorded for Fe^3+^ and Cu^2+^ single experiments when compared to the other pH levels. The UV–vis spectra of the Fe^3+^ alone and the mixture of Fe^3+^ and Cu^2+^ (simultaneous or sequential) closely matched that of the isolated **3-Fe** complex and **3-Fe** with Cu^2+^ added, respectively. Taken together, these results suggest that iron and copper may not compete for the same binding sites on lusichelin C (**3**), and at physiologically relevant pH the compound may play an ecological role as both a siderophore and a chalkophore. However, more detailed binding studies, including interactions with other metals, are needed to fully understand the metallophore properties of this compound.

**Figure 7:**
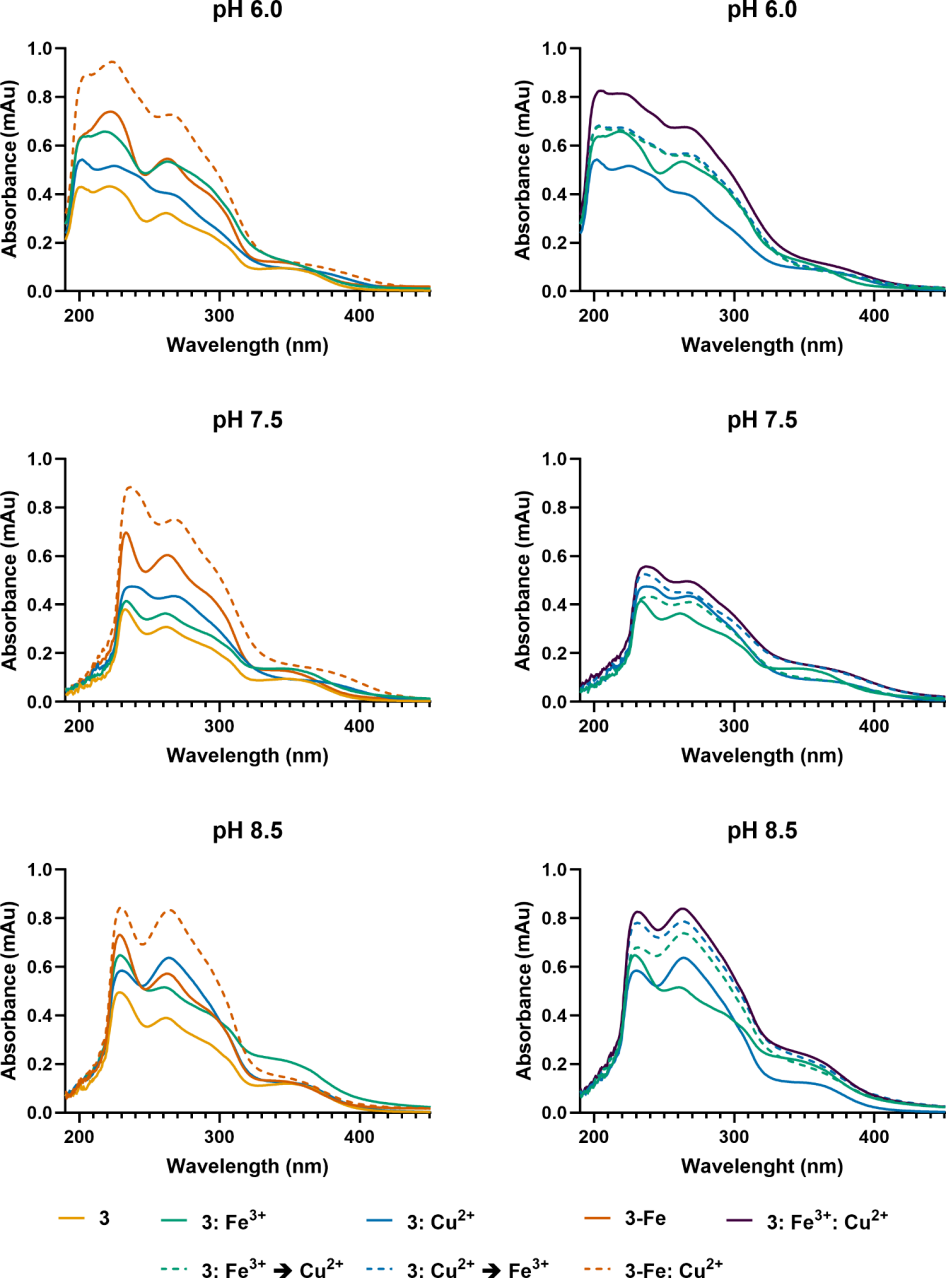
UV–vis absorbance spectra of compound **3**, along with binding experiments with FeCl_3_ (Fe^3+^) and CuSO_4_ (Cu^2+^) in equimolar amounts at pH 6.0, 7.5, and 8.5: compound **3** (yellow line), **3:Fe** ^**3+**^ (green line), **3:Cu** ^**2+**^ (cyan line), **3:Fe** ^**3+**^ **→ Cu** ^**2+**^ (FeCl_3_ added first, followed by CuSO_4_; green dashed), **3:Cu** ^**2+**^ **→ Fe** ^**3+**^ (CuSO_4_ added first, followed by FeCl_3_; cyan dashed), **3:Fe** ^**3+**^ **:Cu** ^**2+**^ (simultaneous addition of FeCl_3_ and CuSO_4_; purple line), **3-Fe** (preisolated **3-Fe** complex; orange line), and **3-Fe:Cu** ^**2+**^ (preisolated **3-Fe** complex with CuSO_4_ added; orange dashed).

### Cytotoxic Activity of Lusichelins

Our previous bioactivity-guided discovery study^19^ identified *L. coriacea* LEGE 07167 as a promising source of novel cytotoxic compounds. To correlate the activity observed in the fractions with that of the pure compounds, we assessed the cytotoxicity of lusichelins against the same panel of human colon carcinoma HCT 116 cells using both monolayer (2D) and spheroid (3D) cell cultures. In the monolayer assay, compounds **2**, **3**, and **3-Fe** showed cytotoxicity with IC_50_ values of 1.16, 1.58, and 0.45 μM, respectively (SI Table S3). However, in the 3D cell cultures, only compound **2** retained the cytotoxicity (SI Table S3). Unlike 2D cultures, 3D tumor spheroids more closely mimic the physiology and structure of tumors, making them a more effective tool for screening potential anticancer candidates.^19^ Due to their increased complexity, IC_50_ values in 3D models are typically at least four times higher than those observed in 2D cultures for other natural products.^41,42^ Notably, compound **2**, the most potent compound in this study, differs from the inactive compound **1** only in the configuration of the stereocenter at C-19, underscoring the critical role of stereochemistry in determining the bioactivity of these compounds.

The development of multidrug resistance (MDR) in cancer is a significant hurdle in treatment, often driven by the overexpression of P-glycoprotein (ABCB1).^43^ This transmembrane protein, encoded by the *ABCB1* gene and a member of the ATP-binding cassette transporter family, functions as a broad-spectrum efflux pump, removing a wide range of chemotherapeutic drugs from cancer cells. Consequently, this reduces intracellular drug concentrations and promotes MDR. Therefore, compounds that can modulate or inhibit ABCB1 function represent promising strategies for overcoming MDR in cancer therapy.^44^ Lusichelins were evaluated for their ability to reverse ABCB1-mediated MDR in mouse T-cell lymphoma L5178Y cells transfected with *ABCB1* (L5178Y-MDR) and their sensitive counterpart (L5178Y-PAR). Compounds **2**, **3**, and **3-Fe** exhibited cytotoxicity (<10 μM) against both cell lines. However, only compound **2** demonstrated selectivity toward MDR phenotypes with a relative resistance ratio of 0.58 (SI Table S3). To further assess their effects, lusichelins were evaluated using the rhodamine-123 ABCB1 transport assay. Since ABCB1 actively transports rhodamine-123 out of cells, compounds that inhibit or modulate this process led to intracellular accumulation of the dye, indicating a potential reversal of MDR. Only compounds **1** and **2** effectively reversed the MDR phenotype at 2 μM, with fluorescence activity ratios (FAR) of 28 and 14.9, respectively. In comparison, tariquidar at 0.2 μM, the positive control, exhibited a FAR value of 98.2 (Figures S47–S49).

## Conclusions

Natural products play a pivotal role in drug discovery and development, particularly in the treatment of cancer and infectious diseases.^45^ However, since the 1990s, the pharmaceutical industry’s interest in natural product research has declined owing to significant technical challenges, such as the frequent rediscovery of known molecules and the scarcity of natural products. Traditional biodiscovery approaches primarily relied on cell-based or target-based assays.^46^ Nonetheless, recent advancements in analytical tools, genome mining, bioinformatics, and microbial culturing techniques have reignited interest in harnessing natural products for drug development.^46,47^ Cyanobacteria, given their biosynthetic potential, are emerging as a promising source for discovering new secondary metabolites.^3,16^ In our pursuit of anticancer compounds from cyanobacteria within our in-house culture collection (LEGE-CC), we combined advanced mass spectrometry metabolomics with robust cell-based assays.^19^ As a result of this preliminary screening, this study successfully reports the discovery of lusichelins A–E (**1**–**5a**), a new group of bioactive metallophores isolated from the marine cyanobacterium *Lusitaniella coriacea* LEGE 07167. Despite structural similarities to bacterial salicyl-thiazol­(in)­e siderophores such as yersiniabactin,^23^ piscibactin,^21^ and pyochelin,^48^ lusichelins exhibit an unprecedented structural framework. Genome sequencing identified a putative BGC for lusichelins, encoding hybrid NRPS-PKS genes alongside signature genes indicative of metal-dependent regulation and transport, strongly suggesting their role as metallophores. Further investigation revealed that rather than iron limitation, the type of salt used in the culture medium was the key factor influencing lusichelin production. The synthetic TM salt is rich in several trace elements that might influence the production of these compounds. Additionally, lusichelins effectively chelate copper at high concentrations. These findings suggest that lusichelins potentially play a crucial role in cyanobacterial physiology and ecology by aiding in the acquisition of essential trace metals such as iron while simultaneously providing protection against copper toxicity. This multifunctionality, recently reported for other cyanobacterial metallophores like leptochelins and fatuamide A, highlights the versatility of these chelating molecules in adapting to metal fluctuations in the environment.^14,15^ These discoveries raise new questions, particularly regarding the regulatory mechanisms involved in cyanobacterial metallophore production and the potential roles of these molecules in ecological interactions.

Beyond their metallophore properties, lusichelins, particularly lusichelin B (**2**), exhibit promising anticancer potential. Lusichelin B (**2**) demonstrated potent cytotoxicity against both monolayer cultures and tumor spheroids of human colon carcinoma HCT 116 cells, and it effectively overcame cancer MDR by modulating the efflux activity of the ABCB1 transmembrane pump.

The discovery of lusichelins as dual-function metallophores and anticancer agents expands our understanding of cyanobacterial secondary metabolism and highlights their potential as drug leads. These findings reinforce the continued relevance of natural products in modern drug discovery and open new avenues for the development of innovative therapies.

## Experimental Section

### General Experimental Procedures

The optical rotations were obtained using a JASCO P-2000 polarimeter with SpectraManager 2.14.02 software. The infrared spectra were collected on a Nicolet iS5 FTIR spectrometer (ThermoScientific) with OMNIC 9.8.372 software. The UV–vis spectrum was acquired on a 1600PC spectrophotometer (VWR) with a 1 cm path length quartz cuvette. ECD spectra were recorded on a JASCO J-815 CD spectrometer. NMR spectra were acquired at the service of the Materials Center of the University of Porto (CEMUP) and the Analytical Facilities at the Universidad de Santiago. 1D and 2D NMR data of compounds **1**–**5** were recorded on a Bruker Avance III HD (^1^H 600 MHz, ^13^C 151 MHz) equipped with a 5 mm cryoprobe and controlled by TopSpin 3.6.1; data of compound **3** were acquired on a Bruker Avance III (^1^H 400 MHz,^13^C 101 MHz) controlled by TopSpin 3.2. The HECADE experiment of **3** was run at the Analytical Facilities Universidad de Santiago de Compostela in Bruker NEO (^1^H 750 MHz, ^13^C 187.5 MHz) equipped with a PA-TXI-HFCN (^1^H-^19^F/^13^C/^15^N) probe. ^1^H and ^13^C chemical shifts are expressed in δ (ppm), referenced to the solvent used, and the proton coupling constants *J* are in hertz (Hz). Spectra were assigned using the appropriate COSY, HSQC-edited, HMBC, NOESY and ROESY pulse sequences. LC-HRESIMS/MS analyses of compounds **1**–**5** (0.5 mg/ml) were performed on an UltiMate 3000 UHPLC (Thermo Fisher Scientific) system composed of an LPG-3400SD pump, WPS-3000SL autosampler, and VWD-3100 UV/vis detector coupled to a Q Exactive Focus Hybrid Quadrupole-Orbitrap mass spectrometer controlled by a Q Exactive Focus Tune 2.9 and Xcalibur 4.1 (Thermo Fisher Scientific). Full positive scan mode was used, setting a capillary temperature of 262.5 °C and spray voltage of 3.5 kV, at the resolution of 70,000 fwhm (*m*/*z* range of 150–2000), and data-dependent MS^2^ (ddMS^2^, Discovery mode) at the resolution of 17,500 fwhm (isolation window used was 3.0 amu and normalized collision energy was 35). Chromatographic separations were performed either using column chromatography with silica gel or the Pure C-850 FlashPrep chromatography system (Büchi, Switzerland) with prepacked columns and ACS grade solvents. HPLC was performed on a Waters Alliance e2695 Separations Module instrument coupled to a photodiode array detector (Waters 2998 PDA) and an automatic Waters Fraction Collector III (Waters, Mildford, MA, USA) or on a Thermo Scientific UltiMate 3000 HPLC instrument, with UV detection (254 nm), using an ACE Excel 3 C18-AR (3 μm × 7.5 mm × 4.6 mm) column and HPLC gradient grade solvents.

### Standard Cyanobacterium Culture Conditions

*Lusitaniella coriacea* LEGE 07167 was collected (May 2007) on a beach on the north Atlantic coast of Portugal.^49^ This strain was deposited in the LEGE-CC - Blue Biotechnology and Ecotoxicology Culture Collection (https://lege.ciimar.up.pt/). The cultures were grown using Z8 medium (Z8-TM) supplemented with 25‰ synthetic sea salts (Tropic Marin Pro-Reef, Tropic Marin, Berlin, Germany) and 1‰ vitamin B_12_ and maintained under standard laboratory conditions: 25 °C with light/dark cycle of 14/10 h at a light intensity of 10–30 μmol photons m^–2^ s^–1^. Production of biomass for compound isolation was achieved by large-scale cultures of up to 80 L sleeve bags. At the end of exponential phase, cells were harvested through filtration, frozen, and freeze-dried (LyoQuest, Telstar, Terrassa, Spain), yielding 100 g of biomass (dry weight).

### Isolation of Compounds **1**–**5**

The lyophilized biomass was exhaustively extracted with MeOH at room temperature. The resulting extract (48.9 g) was fractionated by normal-phase vacuum liquid chromatography (Silica gel 60, 0.015–0.040 mm, Merck KGaA) eluted with a gradient of mixtures of *n*-hexane/EtOAc (8:1 to 0:1) and EtOAc/MeOH (1:9 to 0:1) that yielded a total of eight fractions (A–H).

The fractions D–G (eluted with mixtures of 2:3:0 to 0:7:3 hexane/EtOAc/MeOH) were joined and subfractionated using the flash system of Pure C-850 FlashPrep (25 g SiO_2_ flash cartridges SiliaSep Silica, Silicycle), with a gradient of 8:2 to 0:1 *n*-hexane/EtOAc (12 mL min^–1^). The fractions eluted with 2:3 to 0:1 *n*-hexane/EtOAc were pooled as they contained the *m*/*z* of interest. Compounds **1**, **2**, **4a**, and **5a** could not be separated in normal phase or reverse phase flash chromatography or semipreparative HPLC. We verified that the particle size of the silica was determinant for the purification of the compounds. The best conditions were achieved using the HPLC analytical column ACE 3 C18-AR (75 × 4.6 mm; 100 Å, 3 μm) with an isocratic mixture of H_2_O/MeCN (3:7; 1 mL min^–1^) as eluent, which yielded 2.06, 2.27, 2.08, and 2.29 mg of compounds **1**, **2**, **4a**, and **5a**, respectively.

Fraction H (eluted with MeOH) was submitted to reverse phase flash chromatography (Pure C-850 FlashPrep) using a 220 g C18 flash cartridge (SiliaSep Silica, Silicycle) as the stationary phase and a gradient of 9:1 to 1:9 of H_2_O/*i*-PrOH over 25 min, maintaining the last mixture for 20 min, at a flow rate of 20 mL min^–1^. A subfraction containing compounds **3** and **3-Fe** was further separated by reverse phase flash chromatography on a 40 g C18 flash cartridge (SiliaSep Silica, Silicycle) with a gradient from 7:3 to 0:1 H_2_O/*i*-PrOH as the eluent at a flow rate of 20 mL min^–1^. The subsequent fraction (eluted with 3:2 H_2_O/*i*-PrOH) was subjected to normal phase column chromatography using 24 g of SiO_2_ and CH_2_Cl_2_/MeOH as eluents, providing the fractions from which the purification was carried out. The fraction eluted at 9:1 CH_2_Cl_2_/MeOH was fractionated in a second normal phase column chromatography (24 g of SiO_2_, CH_2_Cl_2_/MeOH), yielding 45 mg of compound **3-Fe.** The remaining fractions were pooled and purified by HPLC using a semianalytical column ACE 10 C18-AR (250 x10 mm) with an isocratic mixture of H_2_O/MeOH (1:3; 1 mL min^–1^), yielding 12.5 mg of compound **3**.

#### Lusichelin A (**1**)

Pale yellow amorphous solid; [α]_D_^23^ +202.2 (*c* 0.1, CHCl_3_); UV (acetone) λ_max_ (log ε) 341 (3.16) nm; IR (NaCl) *v*_max_ 3400, 2918, 2849, 1742, 1612, 1221, 755 cm^–1^; ^1^H and ^13^C NMR spectroscopic data (CDCl_3_), see Table 1; (+)-HRESIMS *m*/*z* 559.0964 [M + H]^+^ (calcd for C_25_H_27_N_4_O_3_S_4_, 559.0965).

#### Lusichelin B (**2**)

Pale yellow solid; [α]_D_^23^ +152.4 (*c* 0.1, CHCl_3_); UV (acetone) λ_max_ (log ε) 341.5 (3.18) nm; ^1^H and ^13^C NMR data (CDCl_3_), see Table 1; (+)-HRESIMS *m*/*z* 559.0964 [M + H]^+^ (calcd for C_25_H_27_N_4_O_3_S_4_, 559.0961).

#### Lusichelin C (**3**)

Yellow amorphous solid; [α]_D_^24^ +431.6 (*c* 0.1, MeOH); [α]_D_^21^ +466.0 (*c* 0.076, MeOH); [α]_D_^21^ +459.7 (*c* 0.0304, MeOH), [α]_D_^21^ +470.3 (*c* 0.0152, MeOH), UV (MeOH) λ_max_ (log ε) 356.5 (3.45), 262 (3.99), 226.5 (4.1), 222 (4.1) nm; ECD (*c* 1.00 × 10^–4^ M, CH_2_Cl_2_), λ_max_ (Δε) 280.5 (38.5), 255.5 (−7.2), 245 (−2.5) nm; IR (NaCl) *v*_max_ 3400, 2924, 2853, 1601, 1568, 779, cm^–1^; ^1^H and ^13^C NMR data (CDCl_3_), see Table 1; (+)-HRESIMS *m*/*z* 545.0803 [M + H]^+^ (calcd for C_24_H_25_N_4_O_3_S_4_, 545.0804).

#### Lusichelin D (**4a**)

Pale yellow amorphous solid; ^1^H and ^13^C NMR data (CDCl_3_), see Table 2; (+)-HRESIMS *m*/*z* 577.1069 [M + H]^+^ (calcd for C_25_H_29_N_4_O_4_S_4_, 577.1066).

#### Lusichelin E (**5a**)

Pale yellow amorphous solid; ^1^H and ^13^C NMR data (CDCl_3_), see Table 2; (+)-HRESIMS *m*/*z* 577.1069 [M + H]^+^ (calcd for C_25_H_29_N_4_O_4_S_4_, 577.1066).

### Marfey’s of Compound **3**

Compound **3** (0.5 mg) was hydrolyzed with 2 M HCl (500 μL) at 90 °C for 4 h. A 100 μL aliquot of the resulting hydrolysate was neutralized with 1 M NaHCO_3_ (40 μL). To this mixture was added l-FDLA (0.5% w/v in acetone, 200 μL), and the reaction was stirred at 50 °C for 24 h under a nitrogen atmosphere. The solution was then cooled to room temperature, neutralized with 2 M HCl (20 μL), evaporated to dryness, and reconstituted in MeCN (100 μL). The derivatives were analyzed using LC-MS. Chromatographic separation was performed on a reverse phase Kinetex C18 column (2.6 μm, 150 × 4.6 mm) with a gradient elution system and a flow rate of 0.5 mL min^–1^. The mobile phase consisted of H_2_O + 0.1% formic acid (solvent A) and MeCN (solvent B). The gradient program began with a linear increase from 30% to 60% of solvent B over 30 min, followed by an increase to 80% of solvent B over 10 min, and concluded with an isocratic phase at 80% of solvent B for 10 min.

### Computational Methods

The conformational search was carried out in Maestro software using an energy window of 5 kcal/mol. Most relevant conformers were optimized at the opt freq B3LYP/6-31+g­(d,p) level (iefpcm = CH_2_Cl_2_) in Gaussian16 Suit software. Electronic circular dichroism (ECD) simulations were performed at CAM-B3LYP-6-311++g­(2d,p)/DGA1 with 50 electronic states with the COSMO model, parametrized for CH_2_Cl_2_. Experimental and calculated ECD and UV data were handled with SpecDis V 1.7.1.

### Genome Sequencing and Bioinformatics Analysis

After 15 days of cultivation in Z8-TM medium, the freshly grown cyanobacterium’s biomass was sent to the MicrobesNG company to be submitted to a specialized sequencing service (enhanced genome service) including DNA extraction and the use of a combination of short-read Illumina and long-read Nanopore sequencing technologies. After genome sequencing, raw data were submitted to a bioinformatics pipeline, also performed by the company, which includes the identification of the closest reference genomes for reading mapping performed by Kraken 2.^50^ BWA-MEM^51^ was used to assess the quality of the reads, and SPAdes^52^ was used for the *de novo* assembly. The genomic data’s recovered contigs were then examined using the binning tool MaxBin 2.0 ^53^ due to the nonaxenic growth conditions, resulting in a draft genome of *Lusitaniella coriacea* LEGE 07167 (5.97 Mb; Genbank: JBBBDN000000000).

The antiSMASH 7.0 ^18^ software was used to annotate the BGCs of the draft genome, yielding 7 hits, including one hybrid NRPS-PKS matching to a putative *lus* BGC. The identification of enzyme homologues and annotation were performed using Protein BLAST (BlastP) with default settings.

### Iron and Copper Binding Preference of Lusichelin C

The preferred binding of lusichelin C (**3**) was assessed at three different pH levels. Buffer solutions at 0.1 M were prepared for all pH conditions; phosphatase buffer composed of potassium phosphate monobasic and potassium phosphate for pH 6, HEPES buffer (4-(2-hydroxyethyl)-1-piperazineethanesulfonic acid buffer) for pH 7.5, and TAPS buffer (3-((1,3-dihydroxy-2-(hydroxymethyl)­propan-2-yl)­amino)­propane-1-sulfonic acid) for pH 8.5 were prepared in ultrapure water, and their final pH was adjusted using 10 N NaOH. Metal solutions were prepared with FeSO_4_·7H_2_O, FeCl_3_·6H_2_O, and CuSO_4_·5H_2_O in ultrapure water at the same concentration of compound **3** (9.18 mM). Equimolar concentrations of **3**, iron, and/or copper were added to each buffer, and UV–vis spectra were recorded. Sequential metal additions had 5 min intervals between each addition. All glassware used was previously washed with a solution of HCl at 0.5 M to remove any interference caused by other metals.

### Iron and Copper Assays: Experimental Conditions

*Lusitaniella coriacea* LEGE 07167 was grown in ten different experimental conditions (SI Table S4). The main inoculum for the experiments performed in Z8-NaCl medium was adapted to this growth condition one week before the experiment. The cultures were started in 40 mL culture flasks with 12 mL of culture medium and 0.5 mL of a filtrated inoculum. The experiment was carried out in triplicate. The cyanobacterial cultures were incubated for 30 days under standard laboratory conditions (25 °C with light/dark cycle of 14/10 h at a light intensity of 10–30 μmol photons m^–2^ s^–1^). These were shaken continuously at 100 rpm and distributed randomly in piles of three with an empty flask on top to avoid direct light. At the end of the incubation period, the biomass and culture medium were separated by using a 0.41 μm sieve. The freeze-dried biomass was extracted with MeOH and concentrated in a rotary evaporator. Solid phase extraction (SPE) of the culture medium was performed on a Strata C18-E column (55 μm/70 Å; 1 g/6 mL; Phenomenex, Madrid, Spain) eluted with a 95%:5% H_2_O/MeOH mixture to remove remaining salts and then eluted with MeOH.

### Iron and Copper Assays: Data Analysis

Both crude biomass and culture media were analyzed using liquid chromatography–high-resolution electrospray ionization tandem mass spectrometry (LC-HRESIMS/MS). These analyses were performed on a Vanquish HPLC system coupled to an Orbitrap Exploris 120 mass spectrometer and controlled by Q Exactive Focus Tune 2.9 and Xcalibur 4.1 (Thermo Fisher Scientific, Waltham, MA, USA). Full positive scan mode was used, setting a capillary temperature of 262.5 °C and spray voltage of 3.5 kV, at the resolution of 70,000 fwhm (*m*/*z* range of 150–2000), and data dependent MS^2^ (ddMS^2^, discovery mode) at the resolution of 17,500 fwhm (isolation window used was 3.0 amu and normalized collision energy was 35). The extracts (5 μL; 2 mg mL^–1^ in MS-grade MeOH) were separated on an ACE UltraCore 2.5 SuperC18 column (75 × 2.1 mm, ACE, Reading, UK) at 40 °C using a gradient from 99.5% to 10% H_2_O/MeOH/formic acid (95:5:0.1, v/v) to 0.5% to 90% isopropanol/MeOH/formic acid (95:5:0.1, v/v) for 9.5 min, maintaining the last mixture until 15.5 min before returning to the initial conditions, with a flow rate of 0.35 mL min^–1^. MS data were processed using Xcalibur software (ThermoFisher Scientific). Peak area was determined by autointegrating each extracted ion chromatogram peak, with a mass tolerance of 5 ppm, using the calculated masses and reference retention times: compounds **1** and **2**, *m*/*z* 559.0961 [M + H]^+^ (*t*_R_ = 9.90 min); compound **3**, *m*/*z* 545.0804 [M + H]^+^ (*t*_R_ = 9.05 min); compound **3-Fe**, *m*/*z* 597.9919 [M + Fe – 2H]^+^ (*t*_R_ = 4.15 min); and compound **3-Cu**, *m*/*z* 605.9949 [M + Cu – H]^+^ (*t*_R_ = 3.82 min).

GraphPad Prism v10.0.3 (GraphPad, San Diego, CA, USA) was used for statistical analyses. The Shapiro–Wilk test was used to determine the normal distribution of tested values and Student’s *t* test was used to test for significant differences between defined experimental groups. Differential levels with *p* < 0.05 were considered statistically significant. Data are expressed as the mean ± SD.

The octanol–water partition coefficient (logP) of lusichelins **1**–**3** was calculated through the Molinspiration (https://www.molinspiration.com/cgi/properties) calculator of molecular properties.

### Cell Lines and Cell Culture

The human colon carcinoma cell line HCT 116 was obtained from the American Type Culture Collection (ATCC, USA). Cells were maintained in McCoy’s 5A modified media (Merck Life Science S.L.U., Algés, Portugal) supplemented with 10% fetal bovine serum (Biochrom, Berlin, Germany), 1% penicillin/streptomycin, and 0.1% amphotericin B (GE Healthcare, Little Chafont, United Kingdom) and incubated at 37 °C in 5% CO_2_. In culture, cells were tested for mycoplasma contamination.

Mouse T-cell lymphoma cells L5178Y (ECACC Cat. No. 87111908, FDA, Silver Spring, MD, USA) and its *ABCB1*-transfected multidrug-resistant subline (L5178Y-MDR) were cultured in McCoy’s 5A media supplemented with 10% heat-inactivated horse serum, 100 U/L l-glutamine, and 100 mg/L penicillin–streptomycin mixture (Sigma-Aldrich Kft, Budapest, Hungary). The MDR phenotype was maintained by culturing the L5178Y-MDR with 60 ng mL^–1^ colchicine (Sigma-Aldrich Chemie GmbH, Steinheim, Germany).

All cell lines were incubated in a humidified atmosphere at 37 °C and in 5% CO_2_.

### Cytotoxicity Assays with HCT 116 Monolayer Model and HCT 116 Spheroids

HCT 116 cells were detached from the culture flasks by trypsinization, seeded on 96-well plates for the monolayer culture tests at a density of 3.3 × 10^4^ cells mL^–1^, and allowed to adhere for 24 h. The cells were incubated with a serial dilution of pure compounds (0.4–100 μM) for 48 h. Dimethylsulfoxide (0.5%) (Sigma, USA) was used as the solvent for all the substances evaluated in this study; staurosporine (500 nM) was employed as the positive control. After the exposure, 3-(4,5- dimethylthiazol-2-yl)-2,5-diphenyltetrazolium bromide) (MTT) was added at a final concentration of 200 μg mL^–1^ per well for 3 h. Afterward, formazan crystal growth was observed under a microscope and dissolved in DMSO, and the absorbance was measured at 550 nm on a Cytation 5 Cell Imaging Multi-Mode Reader (BioTek, Vermont, USA). The percentage of cell viability was calculated as described in equation 1:

$$\%\mathrm{cell viability}\;\left(\mathrm{to solvent control}\right)=\frac{\mathrm{x̅}\mathrm{absorbance sample}}{\mathrm{x̅}\mathrm{absorbance solvent control}}\times 100$$ 1

For the 3D assays, 200 μL of a suspension of HCT 116 cells at a concentration of 5 × 10^4^ cells mL ^–1^ was seeded on an Ultra-Low Attachment round-bottom 96 well plate (Costar, Corning, New York, NY, USA) and allowed to settle in the flow chamber for 20 min, followed by 5 days of incubation to produce spheroids. Pure compounds ranging from 0.4 to 100 μM (1% DMSO) were applied to the newly generated spheroids for 96 h. After that period, the acid phosphatase viability assay was performed according to Friedrich.^54^ Briefly, following a thorough wash with PBS, 100 μL of sodium acetate buffer (0.1 M) containing *p*-nitrophenyl phosphate (2 mg mL^–1^) was added. The reaction was terminated after 2 h with 10 μL of NaOH (1 N), then the absorbance at 405 nm was measured with a Cytation 5 Cell Imaging Multi-Mode Reader (Biotek). The percentage of cell viability was normalized to the solvent control as specified in equation 1.

### Cytotoxicity Assays with Mouse Lymphoma T-Cells

The cytotoxic profile of lusichelins in a multidrug-resistant mouse lymphoma T-cell line (L5178Y-MDR) and its parental counterpart (L5178Y-PAR) was initially evaluated at concentrations of 20 and 2 μM. For the assay, 100 μL of McCoy’s 5A medium was prepared with the test compounds, followed by the addition of cells at a concentration of 1 × 10^4^ cells mL^–1^, bringing the final volume to 200 μL per well. Control wells contained only cells without the test compounds. After 24 h of incubation, 20 μL of the MTT stock solution (5 mg mL^–1^) was added to each well, and the cells were incubated for an additional 4 h. Subsequently, 100 μL of 10% sodium dodecyl sulfate (SDS) in 0.01 N HCl was added to each well and incubated overnight. The optical density was measured at 540/630 nm using a Multiscan EX ELISA reader (ThermoLabsystems, Cheshire, WA, USA). The percentage of cell inhibition was calculated as outlined in equation 2. Compounds exhibiting cytotoxicity in the preliminary assay were further evaluated to determine their IC_50_ values by using a similar procedure. Specifically, lusichelins A (**1**), B (**2**), and E (**5a**) were tested in McCoy’s 5A medium across a concentration range of 60–0.1 μM, while lusichelin C (**3**) and its iron complex (**3-Fe**) were tested across a range of 100–0.2 μM. The MTT assay was conducted following the same protocol as previously described.

$$\%\mathrm{inhibition}\;\left(\mathrm{to cell control}\right)=100-\left(\frac{\left(\mathrm{x̅}\mathrm{absorbance compound}\right)-\left(\mathrm{x̅}\mathrm{absorbance medium control}\right)}{\left(\mathrm{x̅}\mathrm{absorbance cell control}\right)-\left(\mathrm{x̅}\mathrm{absorbance medium control}\right)}\times 100\right)$$ 2

Data from two valid independent experiments, each with at least three replicates, were collected and analyzed using GraphPad Prism 8. Dose–response curves were generated after transforming the data to a logarithmic scale and normalizing them to the solvent control. Nonlinear regression was applied to calculate the IC_50_ values.

### Rhodamine-123 Accumulation Assay

This assay was used to understand whether lusichelins could act as inhibitors or substrates of the efflux pump ABCB1. L5178Y-MDR cells at a concentration of 2 × 10^6^ cells mL^–1^ were resuspended in 500 μL of serum-free McCoy’s 5A medium. Compounds **1**–**4** and **3-Fe** were added to the cells at final concentrations of 0.2 and 2 μM and incubated for 10 min at room temperature. Tariquidar (0.2 μM) was used as the positive control, and DMSO (2%) was used as the solvent control. The fluorescent dye rhodamine-123 was added to each sample at a concentration of 5.2 μM following incubation at 37 °C for 20 min. For flow cytometry analysis, cells were washed twice and resuspended in 1 mL of PBS. Fluorescence was measured with a CyFlow flow cytometer (Partec, Münster, Germany). The fluorescence activity ratio (FAR) was calculated by dividing the mean fluorescence intensity (FL-1) of treated MDR cells against the FL-1 of untreated cells.

## Supplementary Material



## Data Availability

The genome sequence of *Lusitaniella coriacea* LEGE 07167 is available at GenBank (Accession: JBBBDN000000000). The NMR data for the lusichelins have been deposited in the Natural Products Magnetic Resonance Database (NP-MRD; www.np-mrd.org) and can be found at NP0350681 (lusichelin C), NP0350682 (lusichelin A), NP0350683 (lusichelin B), NP0350684 (lusichelin D), and NP0350685 (lusichelin E). HRESIMS/MS spectra of lusichelins A-E is available in MassIVE (ftp://massive-ftp.ucsd.edu/v09/MSV000097074/).

## Notes

Bioinformatics, spectrometric and spectroscopic data, and biological assays of lusichelins (PDF)
