# Supplementary material for: Decoding Lusichelins A–E: An In-Depth Look at the Metallophores of Lusitaniella coriacea LEGE 07167–Structure, Production, and Functionality
Source: J Nat Prod. 2025 May 21;88(6):1319–33. doi: 10.1021/acs.jnatprod.5c00204 (PMC12210259; doi:10.1021/acs.jnatprod.5c00204)
Supplement: Supplementary file 1 [file np5c00204_si_001.pdf]

Supporting Information

Decoding Lusichelins A-E: An In-Depth Look at the Metallophores of *Lusitaniella coriacea* LEGE 07167 – Structure, Production, and Functionality

Maria Lígia Sousa[1], Leonor Ferreira[1,2], Dora Ferreira[1], Abel M. Forero[3], Raquel Castelo-Branco [1,2], Nikoletta Szemerédi[4], Gabriella Spengler[4], Jaime Rodríguez[3], Carlos Jiménez[3], Pedro Nuno Leão[1], Vitor Vasconcelos[1,2], Mariana Alves Reis[1,*]

[1] CIIMAR/CIMAR, Interdisciplinary Centre of Marine and Environmental Research, University of Porto, 4450-208 Matosinhos, Portugal
[2] Departamento de Biologia, Faculdade de Ciências, Universidade do Porto, Rua do Campo Alegre, Edifício FC4, 4169-007 Porto, Portugal.
[3] CICA - Centro Interdisciplinar de Química e Bioloxía e Departamento de Química, Rua As Carballeiras, Campus do Elviña, Universidade da Coruña, 15071, A Coruña, Spain
[4] Department of Medical Microbiology, Albert Szent-Györgyi Health Center and Albert Szent-Györgyi Medical School, University of Szeged, Semmelweis utca 6, 6725 Szeged, Hungary

Table of contents

Table S1. Bioinformatics annotation of *lus* BGC........ 4

Table S2. Predicted adenylation domain substrate specificities for the *lus* BGC using antiSMASH v7.0, NRPSsp, and NRPS Predictive Blast 6

Table S3. Cytotoxic activity of lusichelins A-D (**1-5a**) and Fe-lusichelin C complex (**3-Fe**) against HCT116 colon cancer cells (2D and 3D cell models) and mouse T-cell lymphoma cells L5178Y (PAR: sensitive and MDR: transfected with ABCB1) 6

Table S4. Iron and copper assays: experimental conditions........ 6

Figure S1. (**A**) Total ion chromatograms (ESI positive mode) of the vacuum liquid chromatography (VLC) fractions D-G, along with the (+)-HRESIMS spectra corresponding to the protonated molecules at *m/z* 559 (t_R = 10.5 min) and *m/z* 577 (t_R = 10.0 min). (**B**) Total ion chromatogram (ESI positive mode) of the VLC fraction H with the (+)-HRESIMS spectra corresponding to the protonated molecules at *m/z* 545 (t_R = 9.79 min) and *m/z* 597 (t_R = 4.75 min). 7

Figure S2. ^{1}H NMR spectrum for **1** in $CDCl_3$ (600 MHz)........ 8

Figure S3. ^{13}C NMR spectrum for **1** in $CDCl_3$ (151 MHz)**.** 8

Figure S4. ^{1}H-^{1}H COSY spectrum for **1** in $CDCl_3$ (600 MHz)........ 9

Figure S5. Multiplicity-edited HSQC spectrum for **1** in $CDCl_3$ (600 MHz, 151 MHz) 9

Figure S6. HMBC spectrum for **1** in $CDCl_3$ (600 MHz, 151 MHz) 10

Figure S7. Total ion chromatogram (ESI positive mode) and the (+)-HRESIMS of **1.**........ 10

Figure S8. UV spectrum of **1**........ 11

Figure S9. IR spectrum of **1**. 11

Figure S10. ^{1}H NMR spectrum for **2** in $CDCl_3$ (600 MHz)........ 12

Figure S11. ^{13}C NMR spectrum for **2** in $CDCl_3$ (600 MHz)........ 12

Figure S12. ^{1}H-^{1}H COSY spectrum for **2** in $CDCl_3$ (600 MHz)........ 13

*E-mail: mreis@ciimar.up.pt

Figure S13. Multiplicity-edited HSQC spectrum for **2** in $CDCl_3$ (600 MHz, 151 MHz) 13
Figure S14.HMBC spectrum for **2** in $CDCl_3$ (600 MHz, 151 MHz) 14
Figure S15. Total ion chromatogram (ESI positive mode) and the HRESIMS of **2**. 14
Figure S16. UV spectrum of **2**................ 15
Figure S17. ^{1}H NMR spectrum for **3** in $CDCl_3$ (400 MHz)................ 16
Figure S18. ^{13}C APT NMR spectrum for **3** in $CDCl_3$ (101 MHz)................ 16
Figure S19. ^{1}H-^{1}H COSY spectrum for **3** in $CDCl_3$ (400 MHz)................ 17
Figure S20. Multiplicity-edited HSQC spectrum for **3** in $CDCl_3$ (400 MHz, 101 MHz) 17
Figure S21. Multiplicity-edited HSQC spectrum for **3** in $CDCl_3$ (400 MHz, 101 MHz) 18
Figure S22. HMBC spectrum for **3** in $CDCl_3$ (400 MHz, 101 MHz) 18
Figure S23. HMBC spectrum for **3** in $CDCl_3$ (400 MHz, 101 MHz) 19
Figure S24. HSQC-TOCSY HECADE spectrum for **3** in $CDCl_3$ (750 MHz, 187.5 MHz) 19
Figure S25. NOESY spectrum of **3** in CD_2Cl_2 (750 MHz)................ 20
Figure S26. LC-MS analysis of Marfey's derivatives................ 21
Figure S27. Total ion chromatogram (ESI positive mode) and the HRESIMS of **3**. 21
Figure S28. IR spectrum of **3**. 22
Figure S29. UV spectrum of **3**................ 22
Figure S30. ^{1}H NMR spectrum for the complex **3-Fe** in $CDCl_3$ (400 MHz)................ 23
Figure S31. Total ion chromatogram (ESI positive mode) and the HRESIMS of the complex **3-Fe**. 23
Figure S32. ^{1}H NMR spectrum for **4** in $CDCl_3$ (600 MHz)................ 24
Figure S33. ^{13}C NMR spectrum for **4** in $CDCl_3$ (151 MHz)................ 24
Figure S34. ^{1}H-^{1}H COSY spectrum for **4** in $CDCl_3$ (600 MHz)................ 25
Figure S35. Multiplicity-edited HSQC spectrum for **4** in $CDCl_3$ (600 MHz, 151 MHz) 25
Figure S36.HMBC spectrum for **4** in $CDCl_3$ (600 MHz, 151 MHz) 26
Figure S37. High-resolution mass spectrometry (HRESIMS) data of **4a** and **4b**. **(A)** Extracted ion chromatograms (within 5 ppm error) for **4a** (t_R = 4.33 min) and **4b** (t_R = 13.88 min). **(B)** (+)-HRESIMS of **4a.** (C) (+)- HRESIMS of **4b.**................ 27
Figure S38. ^{1}H NMR spectrum for **5** in $CDCl_3$ (600 MHz)................ 28
Figure S39. ^{13}C NMR spectrum for **5** in $CDCl_3$ (151 MHz)................ 28
Figure S40. ^{1}H-^{1}H COSY spectrum for **5** in $CDCl_3$ (600 MHz). Moreover, the NMR spectra allowed to infer the presence of the methylester cysteine moiety in **5a** and **5b**. This observation was confirmed by the COSY correlations between the amide proton at δ_H 6.64 d (thiol **5a**)/6.79 d (sulfide dimer **5b**), and methine H22 at δ_H 4.87/ δ_C 53.1 (thiol **5a**); δ_H 4.85/ δ_C 51.0 (sulfide dimer **5b**) and in turn to methylene H21 at δ_H 3.00/ δ_C 26.2 (thiol **5a**); δ_H 3.17/ δ_C 40.0 (sulfide dimer **5b**). 29
Figure S41. Multiplicity-edited HSQC spectrum for **5** in $CDCl_3$ (600 MHz, 151 MHz) 30
Figure S42.HMBC spectrum for **5** in $CDCl_3$ (600 MHz, 151 MHz) 30

Figure S43. High-resolution mass spectrometry data of **5a** and **5b**. **(A)** Extracted ion chromatograms (within 5 ppm error) for **5a** (t_R = 4.76 min) and **5b** (t_R = 11.75 min). **(B)** (+)-HRESIMS of **5a. (C)** (+)- HRESIMS of **5b.** The total ion chromatogram showed a peak at t_R 3.75 min that displayed the $[M+H]^+$ ion at *m/z* 577.1069 in its corresponding (+)-HRESIMS which assigned to the thiol **5a**. Similarly, another peak at t_R 11.76 min was observed in that chromatogram that showed a $[M+H]^+$ ion at *m/z* 1151.1895 and $[M+H]^{2+}$ ion at *m/z* 576.0984 in its (+)-HRESIMS that was assigned to the sufide **5b**.. 31

Figure S44. Comparison of the extracted ion chromatograms of the masses corresponding to lusichelins **1-5b** using different extraction methods (**A**) Data obtained from biomass extracted with MeOH, relative abundance scale was normalized to the highest intensity mass at 1.76E9, showing the presence of lusichelins **1-5a**. (**B**) Data obtained from biomass extracted with $CHCl_2$, relative abundance scale was normalized to the highest intensity mass at 1.18E9, showing that lusichelins **1-2** are the most abundant compounds in the extract. 32

Figure S45. Comparison of the extracted ion chromatograms of the masses corresponding to lusichelins **1-5b** using different extraction methods (**A**) Data obtained from biomass extracted with MeOH, relative abundance scale was normalized to the highest intensity mass in each plot, showing the presence of lusichelins **1-5a**. (**B**) Data obtained from biomass extracted with $CHCl_2$, relative abundance scale was normalized to the highest intensity mass in each plot, showing the presence of lusichelins **1-5b**.. 33

Figure S46. UV–vis absorbance spectra of **3** and **3-Fe**, along with binding experiments with $FeCl_2$ (Fe^{2+}) and $FeCl_3$ (Fe^{3+}) in equimolar amounts at pH 6.0, 7.5, and 8.5: compound **3** (black line), **3: Fe^{2+}** (blue line), **3: Fe^{3+}** (green line), and **3-Fe** (isolated **3-Fe** complex; red line). 34

Figure S47. Modulation of ABCB1-mediated rhodamine-123 efflux. (**A**) Parental L5178Y cells exhibit cytoplasmic accumulation of the dye, while (**B**) L5178Y-MDR cells show reduced accumulation due to the activity of the efflux pump. The Forward Scatter (FSC) versus Side Scatter (SSC) plots display the gated cell population (R1). In the corresponding histograms, the term “Count” on the y-axis represents the number of individual cells within the gated populations M1 or M2, with M3 indicating the total cell count in the sample (R1). The x-axis label “FL1” refers to the mean fluorescence intensity of rhodamine-123... 34

Figure S48. Modulation of ABCB1-mediated rhodamine-123 efflux. L5178Y-MDR cells exposed to (**A**) 2% DMSO (solvent control) show baseline efflux activity, as indicated by rhodamine-123 fluorescence levels. In contrast, cells treated with (**B**) 0.2 µM tariquidar, a known efflux pump inhibitor, display increased intracellular accumulation of rhodamine-123 due to inhibited efflux. The Forward Scatter (FSC) versus Side Scatter (SSC) plots depict the gated cell population (R1). In the corresponding histograms, the y-axis labeled "Count" represents the number of individual cells within the gated populations M1 or M2, while M3 indicates the total cell count of the sample (R1). The x-axis label "FL1" corresponds to the mean fluorescence intensity of rhodamine-123.. 35

Figure S49. Modulation of ABCB1-mediated rhodamine-123 efflux. L5178Y-MDR cells exposed to 2 µM (**A**) of lusichelin A (**1**) and (**B**) lusichelin B (**2**). Both compounds effectively reversed the multidrug resistance (MDR) phenotype, with fluorescence activity ratios (FAR) of 28 and 14.9, respectively. The Forward Scatter (FSC) versus Side Scatter (SSC) plots depict the gated cell population (R1). In the corresponding histograms, the y-axis labeled "Count" represents the number of individual cells within the gated populations M1 or M2, while M3 indicates the total cell count of the sample (R1). The x-axis label "FL1" corresponds to the mean fluorescence intensity of rhodamine-123. 36

Table S1. Bioinformatics annotation of *lus* BGC**.**

**Protein**	**Length (aa)**	**Locus tag ID**	**Closest homologue and origin**	**Identity/ similarity [%]**	**Accession no. (closest homologue)**
LusA	642	WB391_24900	heavy metal translocating P-type ATPase [*Calothrix parasitica*]	67/99	WP_096658751.1
LusB	411	WB391_24905	MFS transporter [*Roseofilum casamattae*]	87/98	WP_283758879.1
LusC	589	WB391_24910	ABC transporter ATP-binding protein [*Roseofilum* sp. SID3]	83/99	MBP0011784.1
LusD	578	WB391_24915	ABC transporter ATP-binding protein [*Roseofilum casamattae*]	90/100	WP_283758881.1
LusE	468	WB391_24920	nickel resistance protein [*Rivularia* sp. IAM M-261]	61/100	GJD20024.1
LusF	366	WB391_24925	precorrin-8X methylmutase [*Roseofilum casamattae*]	82/100	WP_283758903.1
LusG	641	WB391_24930	heavy metal translocating P-type ATPase [*Roseofilum casamattae*]	89/100	WP_283758902.1
LusH	806	WB391_24935	TonB-dependent receptor [*Roseofilum* sp. SID2]	78/100	MBP0023305.1
LusI	527	WB391_24940	benzoate-CoA ligase family protein [*Roseofilum casamattae*]	86/100	WP_283758900.1|
LusJ	152	WB391_24945	4'-phosphopantetheinyl transferase superfamily protein [*Roseofilum* sp. SID3]	84/100	MBP0011802.1
LusK	84	WB391_24950	phosphopantetheine-binding protein [*Roseofilum casamattae*]	88/100	WP_283758898.1
LusL	340	WB391_24955	AraC family transcriptional regulator [*Roseofilum casamattae*]	87/99	WP_283758897.1|
LusM	266	WB391_24960	Class I SAM-dependent methyltransferase [*Roseofilum* sp. Belize Diploria]	75/99	MBP0007657.1
LusN	443	WB391_24965	chorismate-binding protein [*Roseofilum* sp. SID3]	84/99	MBP0011798.1
LusO	126	WB391_24970	hypothetical protein [*Roseofilum* sp. Belize Diploria]	81/97	MBP0007659.1
LusP	500	WB391_24975	3-oxoacyl-ACP synthase [*Roseofilum* sp. SID3]	83/98	MBP0011796.1

LusQ	1149	WB391_24980	amino acid adenylation domain-containing protein [*Roseofilum casamattae*]	83/100	WP_283758892.1
LusR	1621	WB391_24985	SDR family NAD(P)-dependent oxidoreductase [*Roseofilum* sp. SID3]	81/99	MBP0011794.1
LusS	1872	WB391_24990	amino acid adenylation domain-containing protein [*Roseofilum casamattae*]	84/99	WP_283758890.1
LusT	1520	WB391_24995	amino acid adenylation domain-containing protein [*Roseofilum* sp. SID3]	88/99	MBP0011792.1
LusU	2155	WB391_25000	SDR family NAD(P)-dependent oxidoreductase [*Roseofilum* sp. Belize Diploria]	86/100	MBP0007664.1
LusV	1735	WB391_25010	amino acid adenylation domain-containing protein [*Roseofilum* sp. Belize Diploria]	85/99	MBP0007665.1|
LusW	360	WB391_25015	PEP-CTERM sorting domain-containing protein [*Roseofilum casamattae*]	80/97	WP_283758886.1
LusX	467	WB391_25020	cytochrome P450 [unclassified *Microcoleus*]	94/100	WP_293297346.1
LusY	251	WB391_25025	thioesterase domain-containing protein [unclassified *Microcoleus*]	92/98	WP_293297344.1

Table S2. Predicted adenylation domain substrate specificities for the *lus* BGC using antiSMASH v7.0, NRPSsp, and NRPS Predictive Blast

Adenylation domain gene	Adenylation domain location		antiSMASH prediction	NRPSsp prediction			NRPS predictive blast
	start	end		Score	Substrate	Fallout	
LusQ	559	955	Cys	785.5	Cys	0.0297	No prediction
LusS	1030	1439	Cys	812.7	Cys	0.0199	No prediction
LusT	537	941	Cys	777.4	Cys	0.0326	No prediction
LusV	559	960	Cys	812.4	Cys	0.0199	No prediction

Table S3. Cytotoxic activity of lusichelins A-D (**1-5a**) and Fe-lusichelin C complex (**3-Fe**) against HCT116 colon cancer cells (2D and 3D cell models) and mouse T-cell lymphoma cells L5178Y (PAR: sensitive and MDR: transfected with ABCB1)

	IC_{50} ± SD (μM)				
Compound	HCT116 2D	HCT116 3D	L5178Y-PAR	L5178Y-MDR	Relative resistance ratio[a]
**1**	*	*	*	*	
**2**	1.16 ± 0.50	22.01 ± 4.16	6.77 ± 2.96	3.91 ± 0.18	0.58
**3**	1.58 ± 0.12	> 100	5.55 ± 0.32	9.80 ± 0.45	1.77
**3-Fe**	0.45 ± 0.10	> 100	0.60 ± 0.04	1.00 ± 0.06	1.67
**4a**	*	*	*	*	
**5a**	17.63 ± 8.80	*	> 100	*	
Doxorubicin	-	-	0.74 ± 0.07	2.75 ± 0.17	3.72

* did not fit a dose-response curve

[a] Relative resistance ratio = [IC_{50} L5178Y-MDR] / [IC_{50} L5178Y-PAR].

Table S4. Iron and copper assays: experimental conditions

**Condition**	**Origin of inoculum**	**Culture medium**	**$FeCl_3$**	**[$CuSO_4$] nM**
TM	Z8-TM	Z8-TM	+	5
TM -Fe	Z8-TM	Z8-TM	-	5
NaCl	Z8-NaCl	Z8-NaCl	+	5
NaCl -Fe	Z8-NaCl	Z8-NaCl	-	5
NaCl -Fe -Cu	Z8-NaCl	Z8-NaCl	-	-
NaCl -Cu	Z8-NaCl	Z8-NaCl	+	-
NaCl [200 nM Cu]	Z8-NaCl	Z8-NaCl	+	200
NaCl -Fe [200 nM Cu]	Z8-NaCl	Z8-NaCl	-	200
NaCl [450 nM Cu]	Z8-NaCl	Z8-NaCl	+	450
NaCl –Fe [450 nM Cu]	Z8-NaCl	Z8-NaCl	-	450

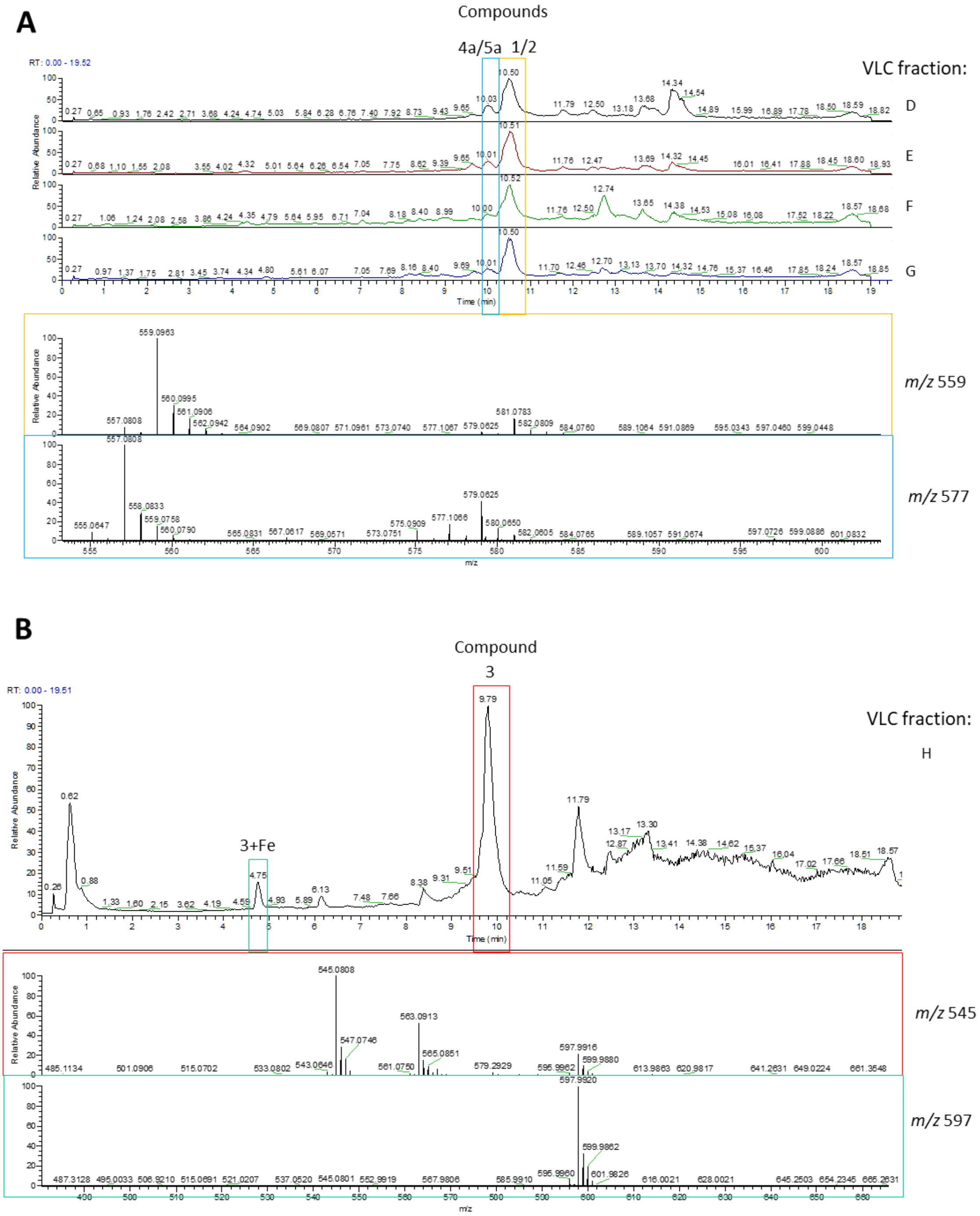

Figure S1. (**A**) Total ion chromatograms (ESI positive mode) of the vacuum liquid chromatography (VLC) fractions D-G, along with the (+)-HRESIMS spectra corresponding to the protonated molecules at *m/z* 559 (t_R = 10.5 min) and *m/z* 577 (t_R = 10.0 min). (**B**) Total ion chromatogram (ESI positive mode) of the VLC fraction H with the (+)-HRESIMS spectra corresponding to the protonated molecules at *m/z* 545 (t_R = 9.79 min) and *m/z* 597 (t_R = 4.75 min).

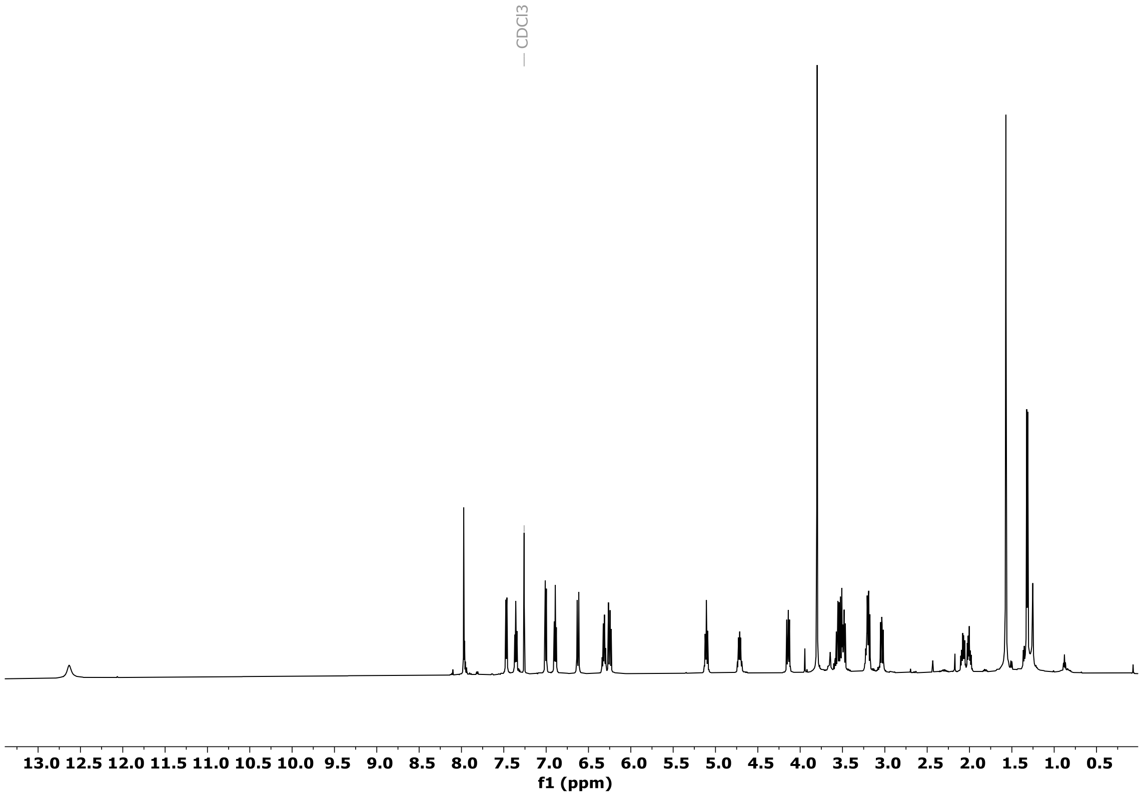

Figure S2. ^{1}H NMR spectrum for **1** in $CDCl_3$ (600 MHz).

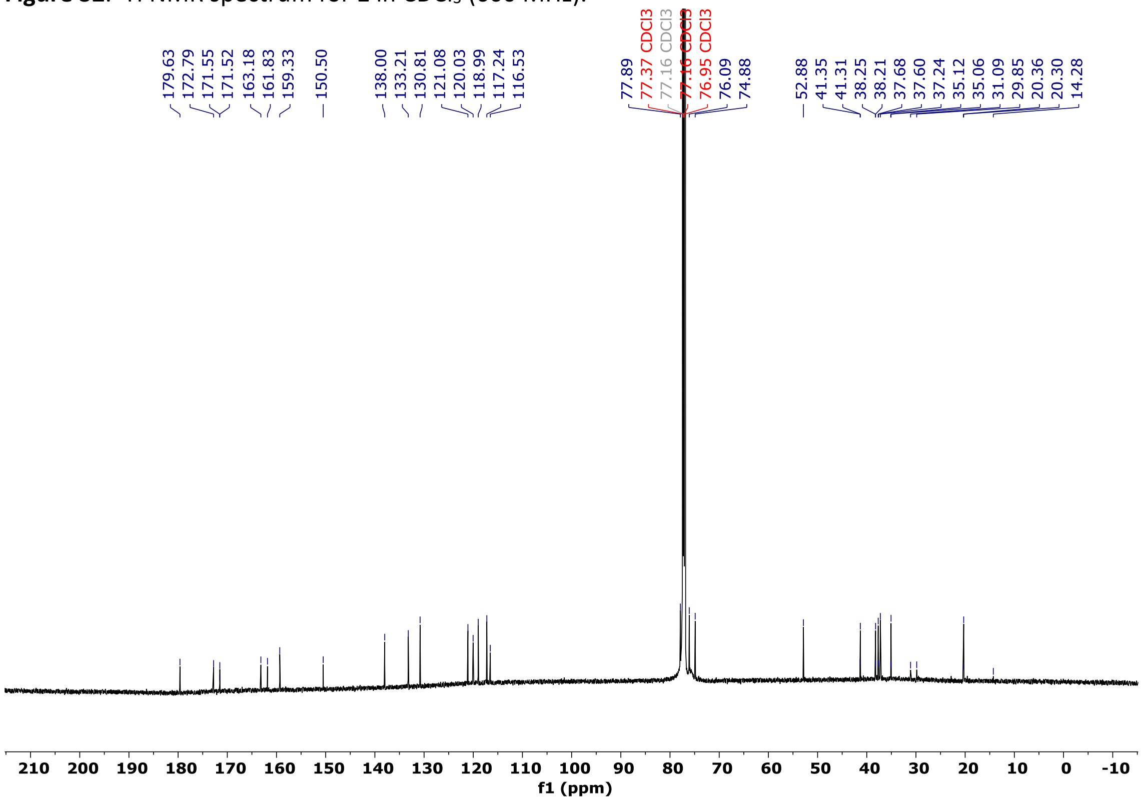

Figure S3. ^{13}C NMR spectrum for **1** in $CDCl_3$ (151 MHz).

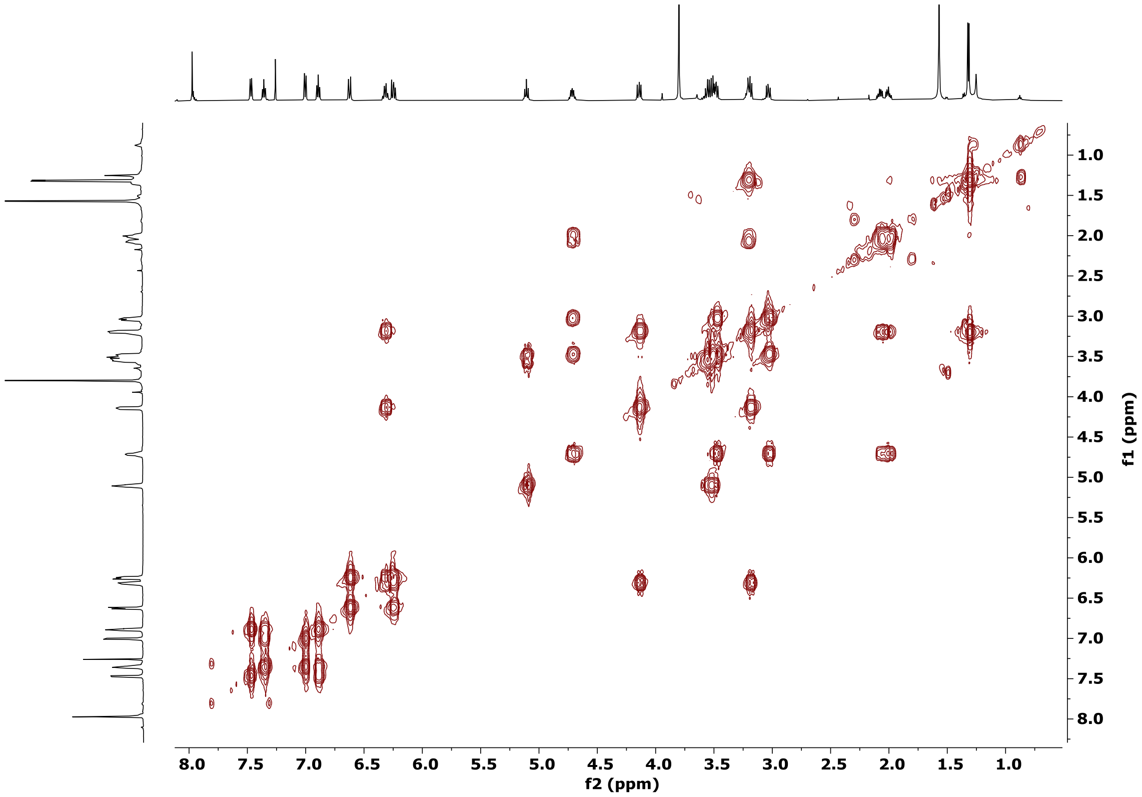

Figure S4. ^{1}H-^{1}H COSY spectrum for **1** in $CDCl_3$ (600 MHz).

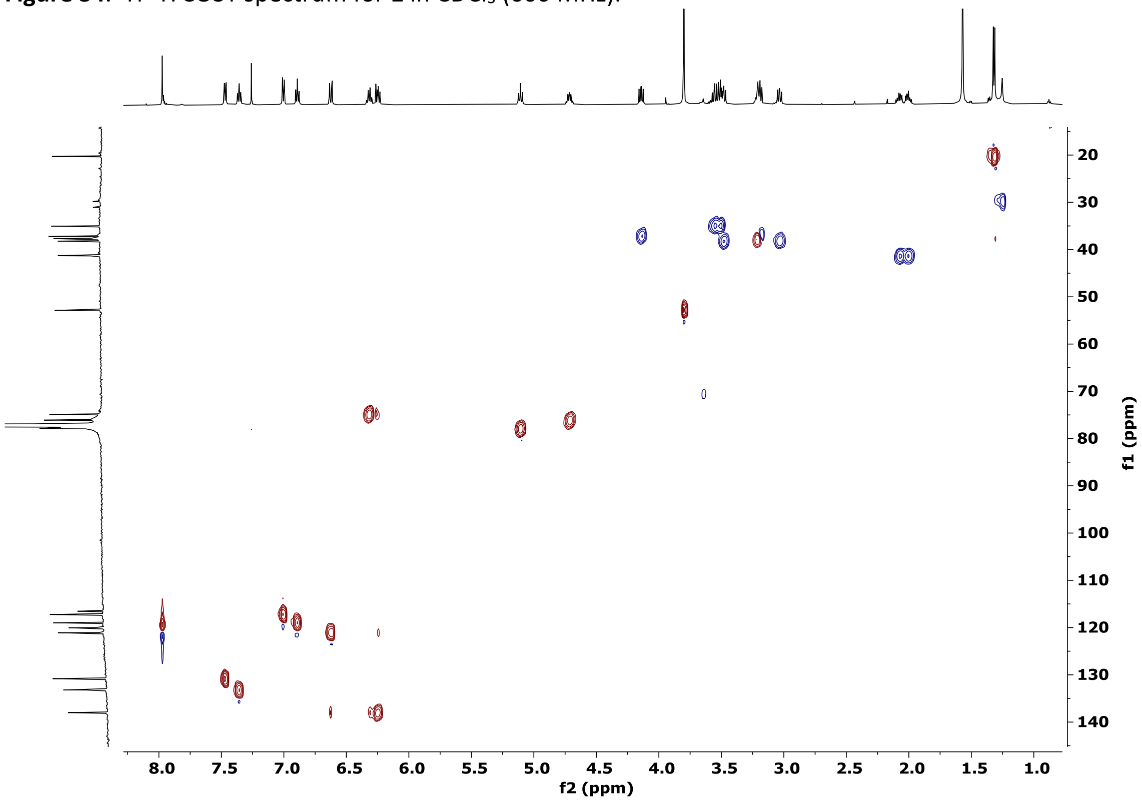

Figure S5. Multiplicity-edited HSQC spectrum for **1** in $CDCl_3$ (600 MHz, 151 MHz)

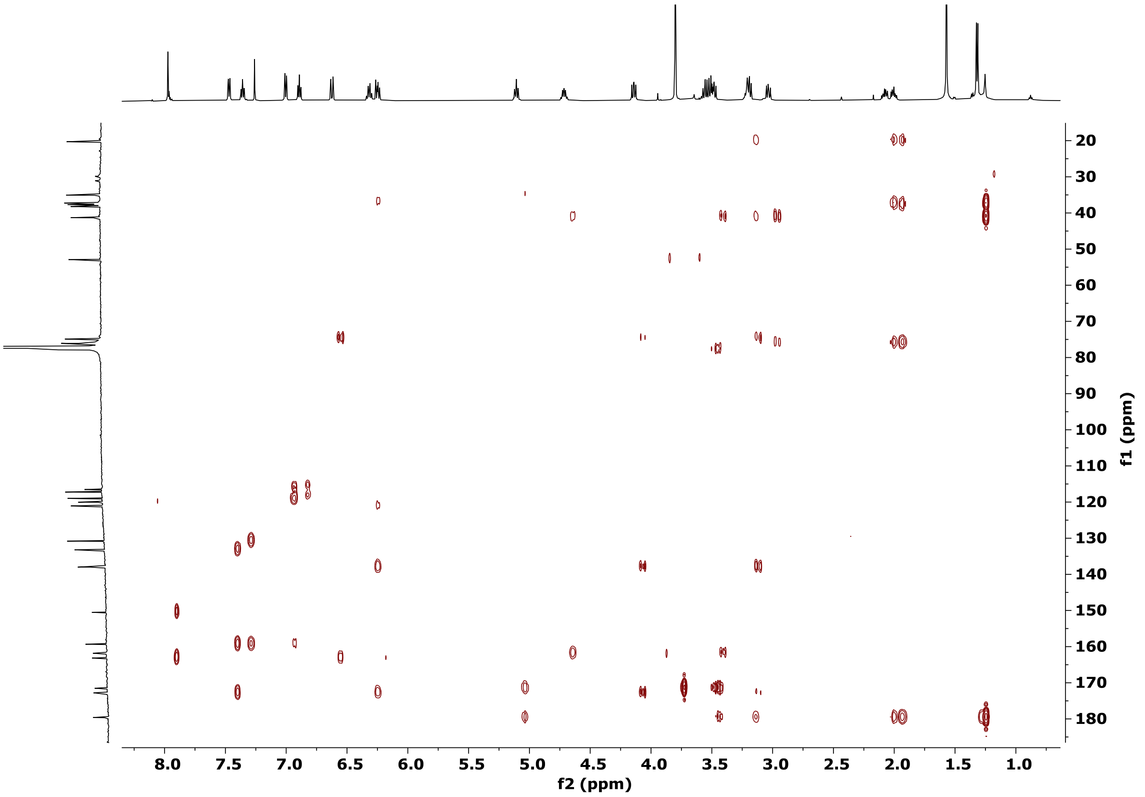

Figure S6. HMBC spectrum for **1** in $CDCl_3$ (600 MHz, 151 MHz)

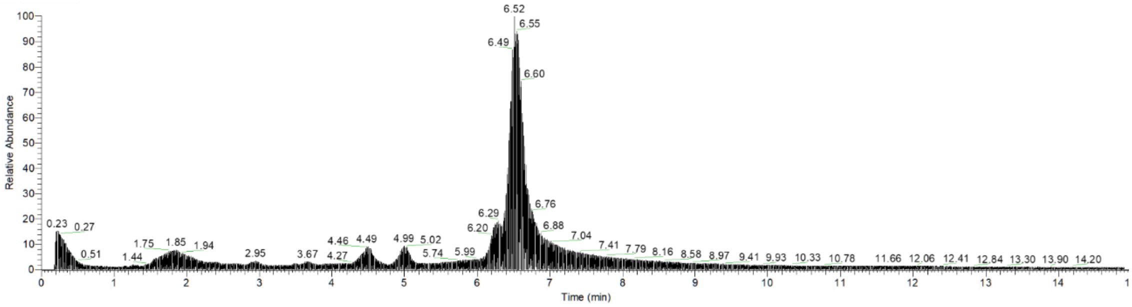

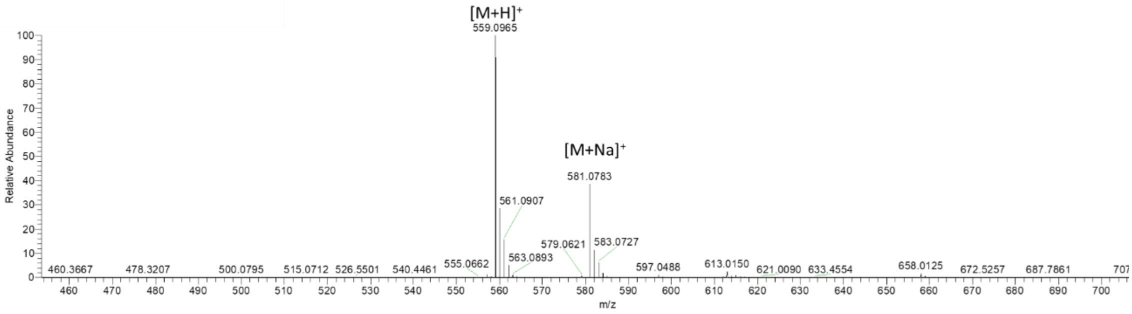

Figure S7. Total ion chromatogram (ESI positive mode) and the (+)-HRESIMS of **1.**

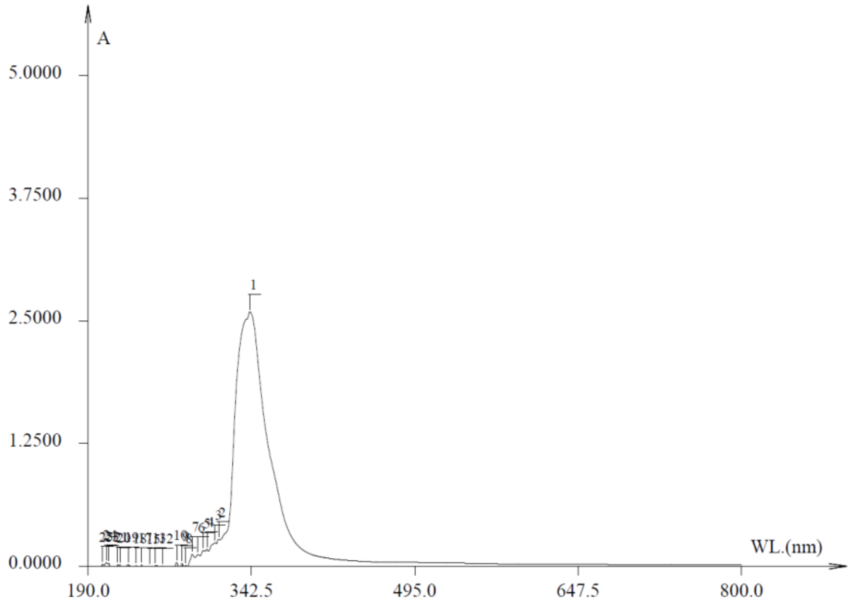

Peak List

ID	WL(nm)	Abs.	%T
1	341.0	2.5940	0.25
2	312.0	0.2750	53.24
3	308.5	0.2398	57.39
4	301.0	0.1722	66.89
5	297.5	0.1618	68.71

Figure S8. UV spectrum of **1**.

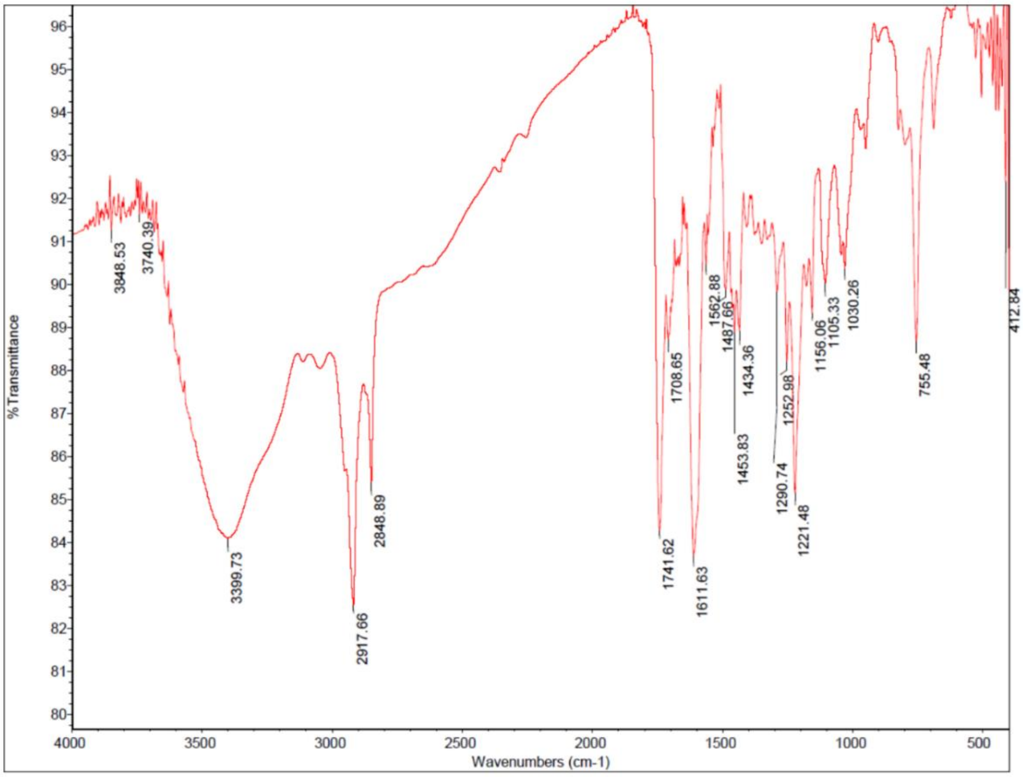

Figure S9. IR spectrum of **1**.

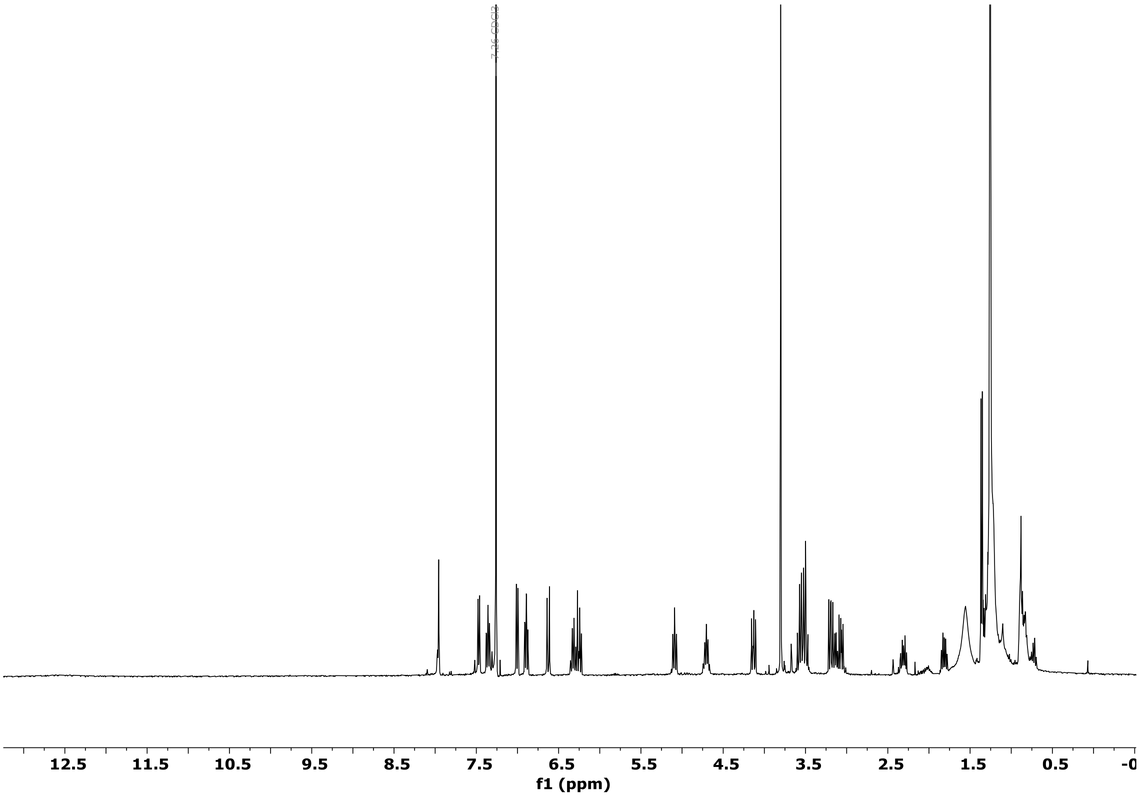

Figure S10. ^{1}H NMR spectrum for **2** in $CDCl_3$ (600 MHz).

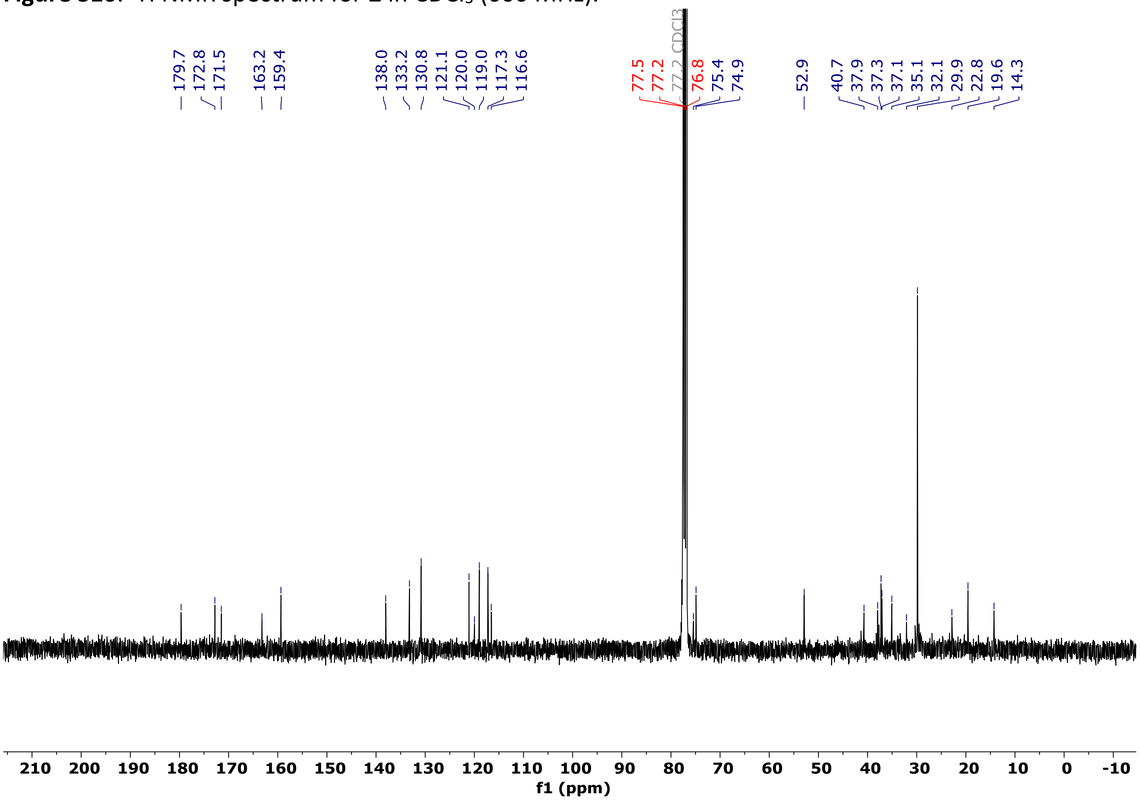

Figure S11. ^{13}C NMR spectrum for **2** in $CDCl_3$ (600 MHz).

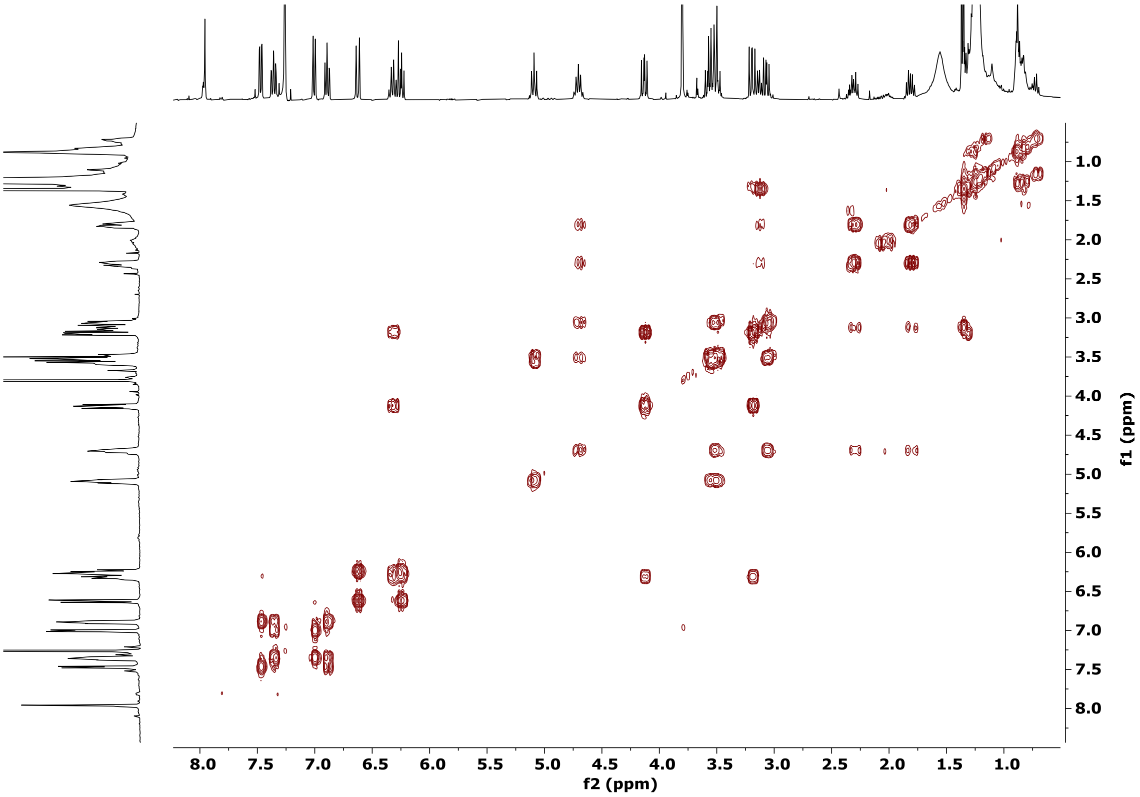

Figure S12. ^{1}H-^{1}H COSY spectrum for **2** in $CDCl_3$ (600 MHz).

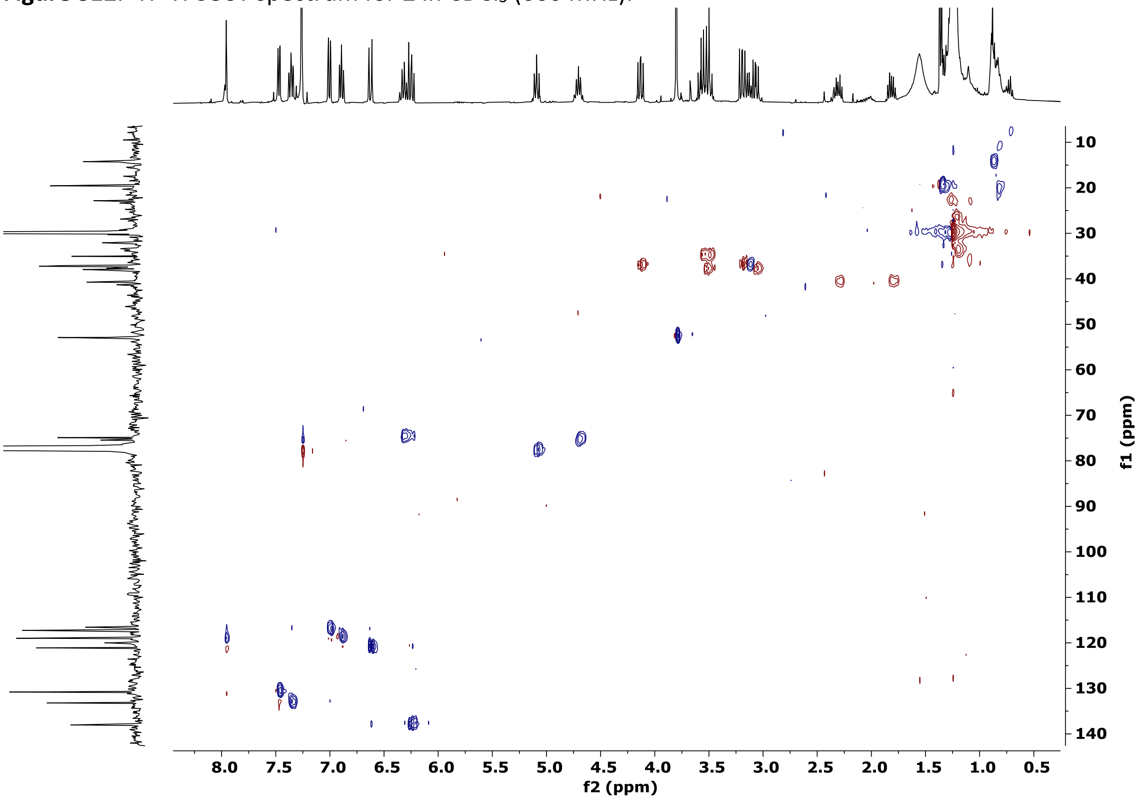

Figure S13. Multiplicity-edited HSQC spectrum for **2** in $CDCl_3$ (600 MHz, 151 MHz)

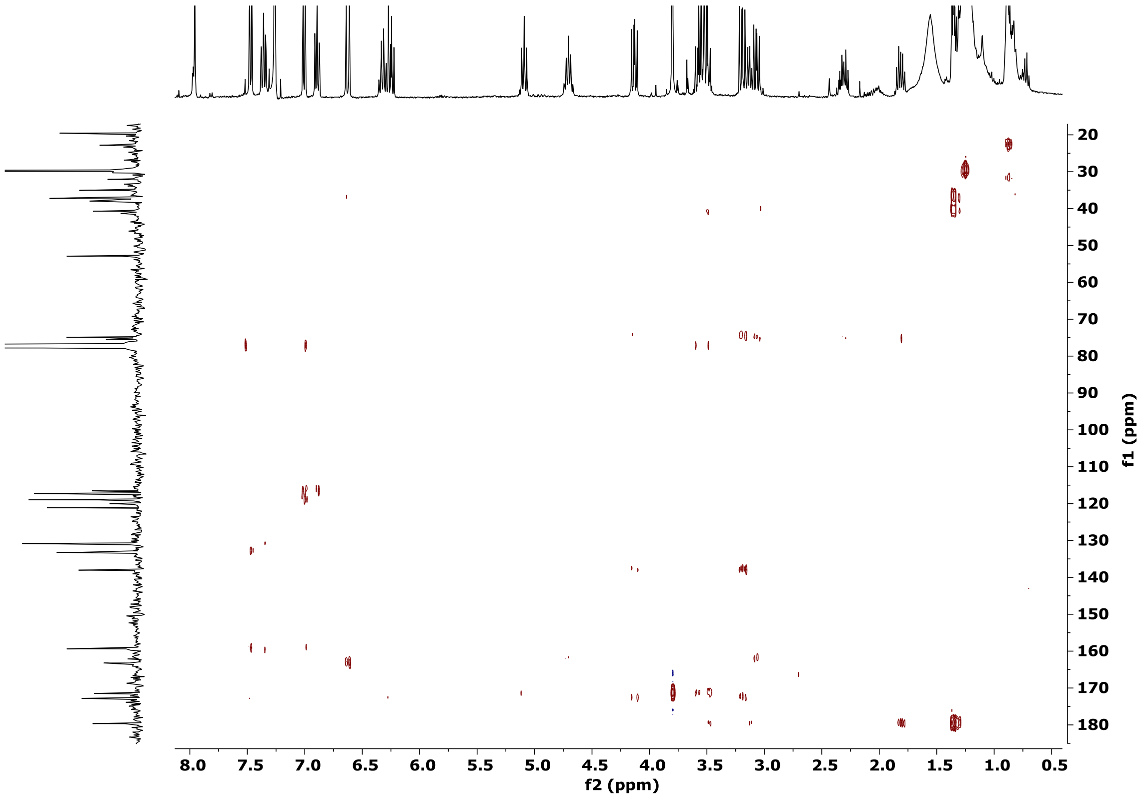

Figure S14.HMBC spectrum for **2** in $CDCl_3$ (600 MHz, 151 MHz)

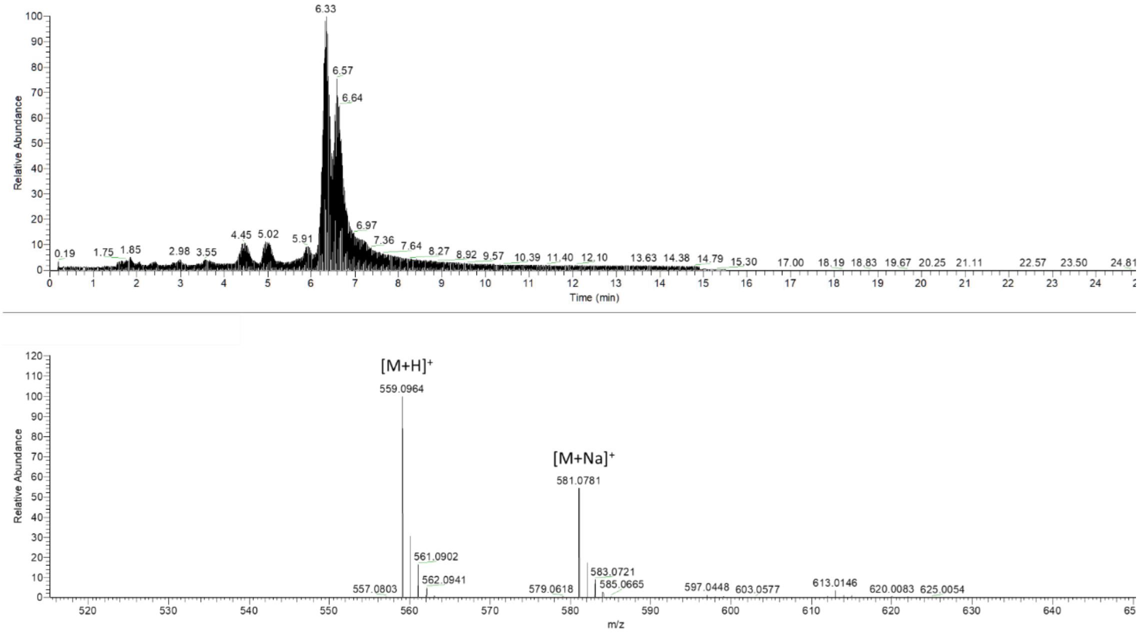

Figure S15. Total ion chromatogram (ESI positive mode) and the HRESIMS of **2**.

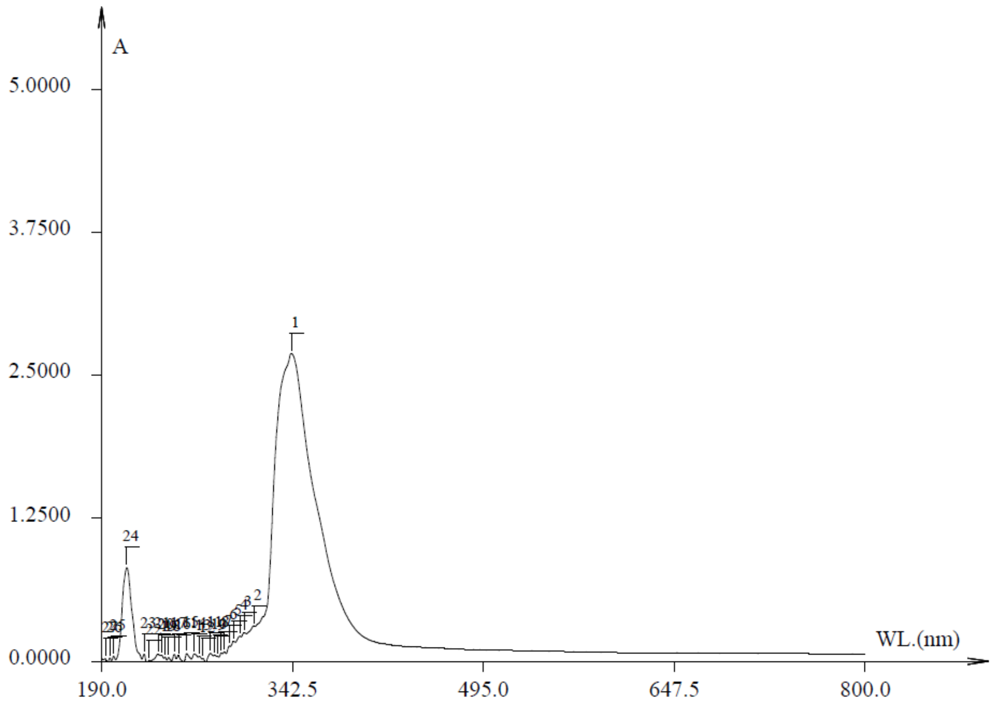

Peak List

ID	WL(nm)	Abs.	%T
1	341.5	2.6922	0.20
2	311.5	0.3098	48.91
3	304.0	0.2509	55.87
4	300.5	0.2192	59.71
5	295.5	0.1759	67.69

Figure S16. UV spectrum of **2**.

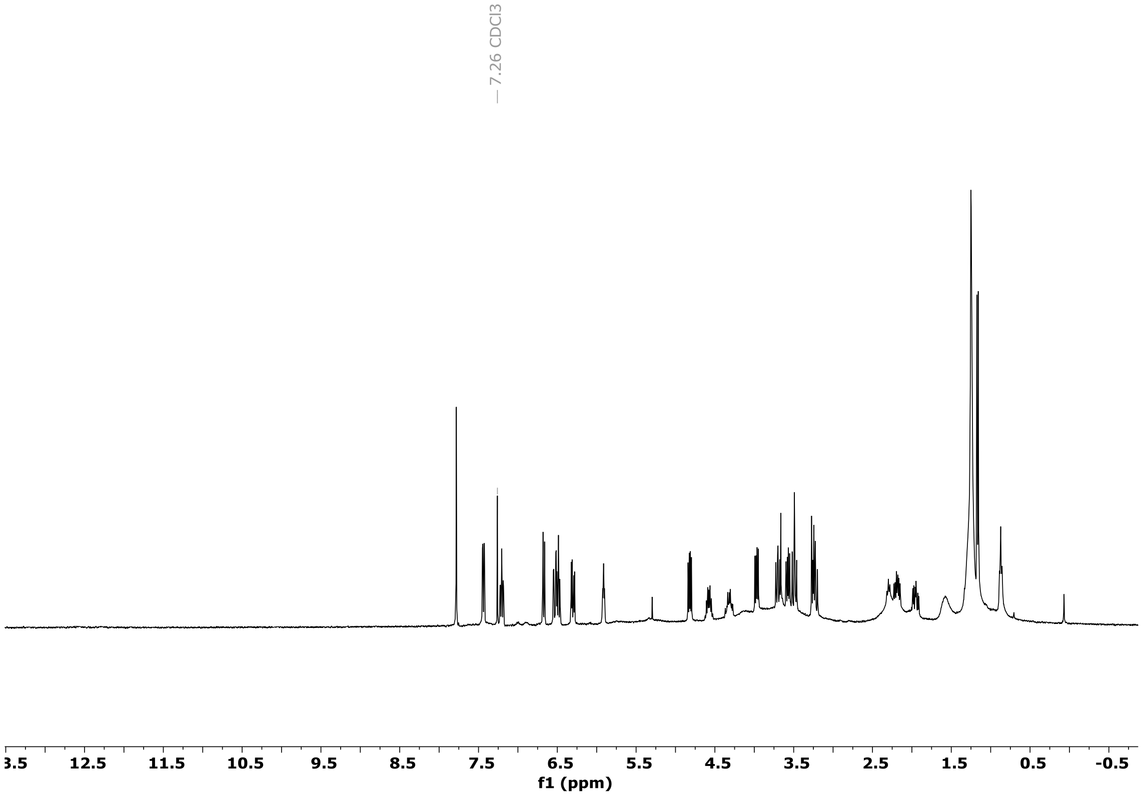

Figure S17. ^{1}H NMR spectrum for **3** in $CDCl_3$ (400 MHz).

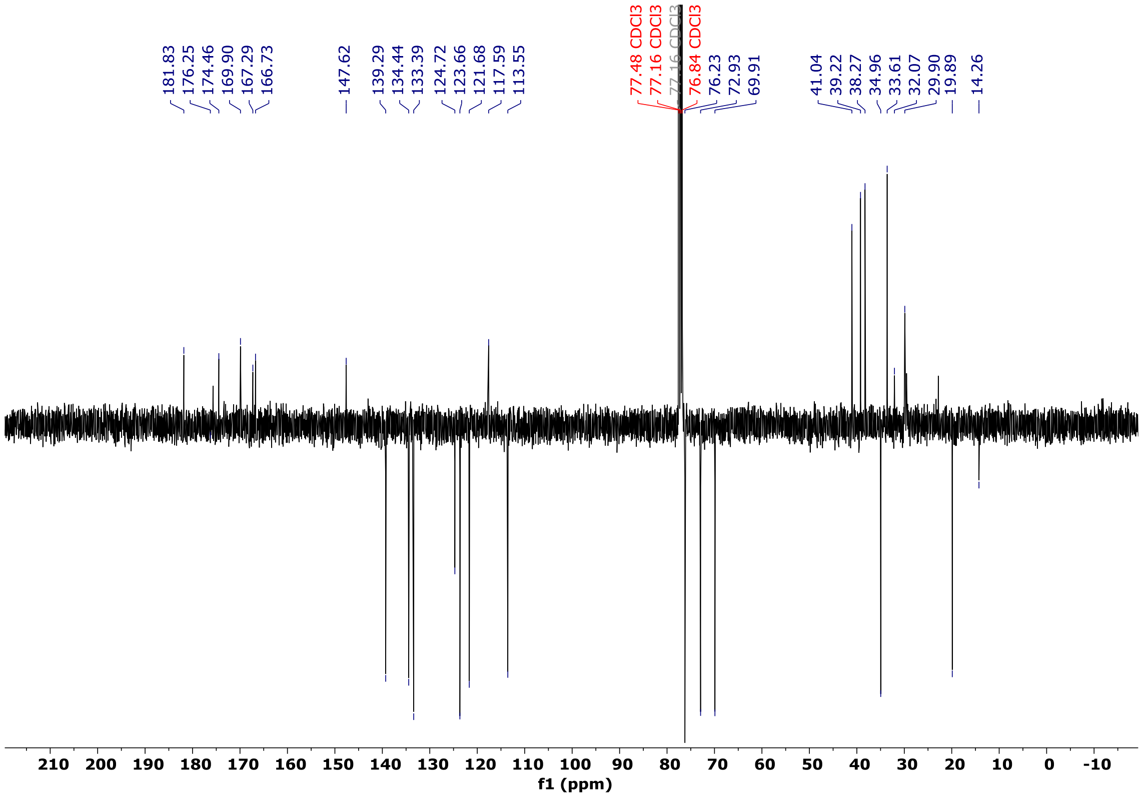

Figure S18. ^{13}C APT NMR spectrum for **3** in $CDCl_3$ (101 MHz).

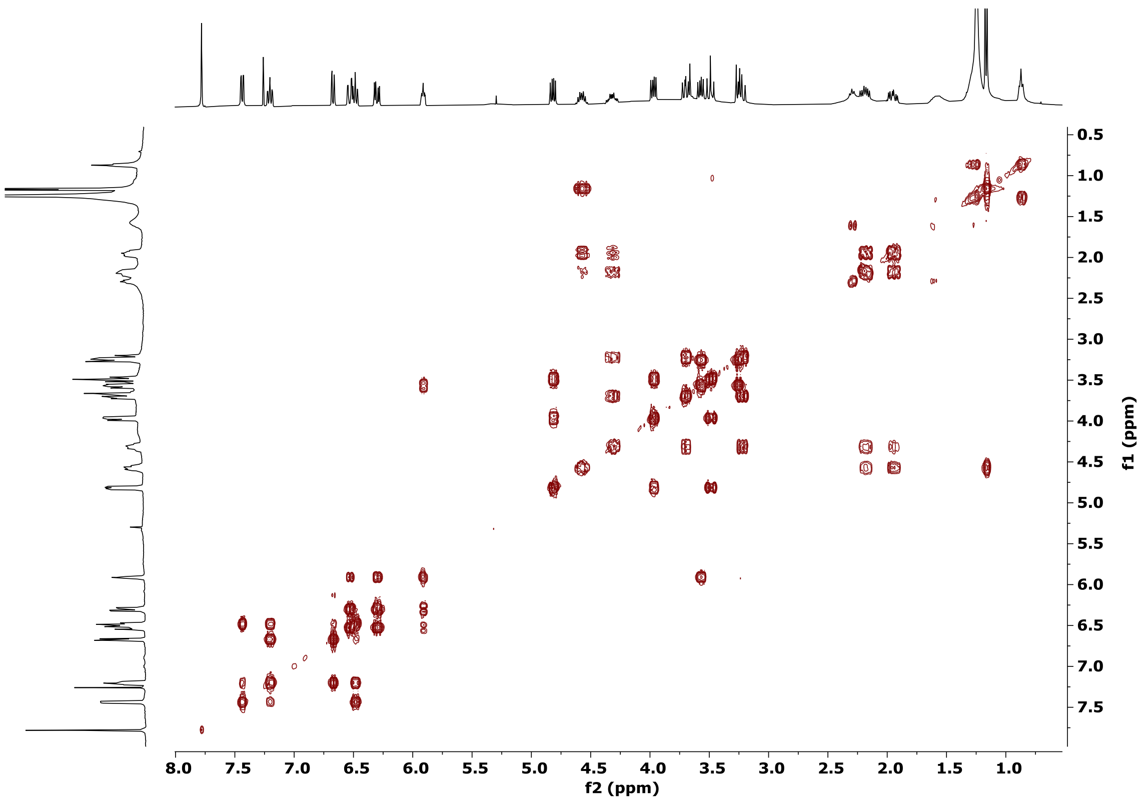

Figure S19. ^{1}H-^{1}H COSY spectrum for **3** in $CDCl_3$ (400 MHz).

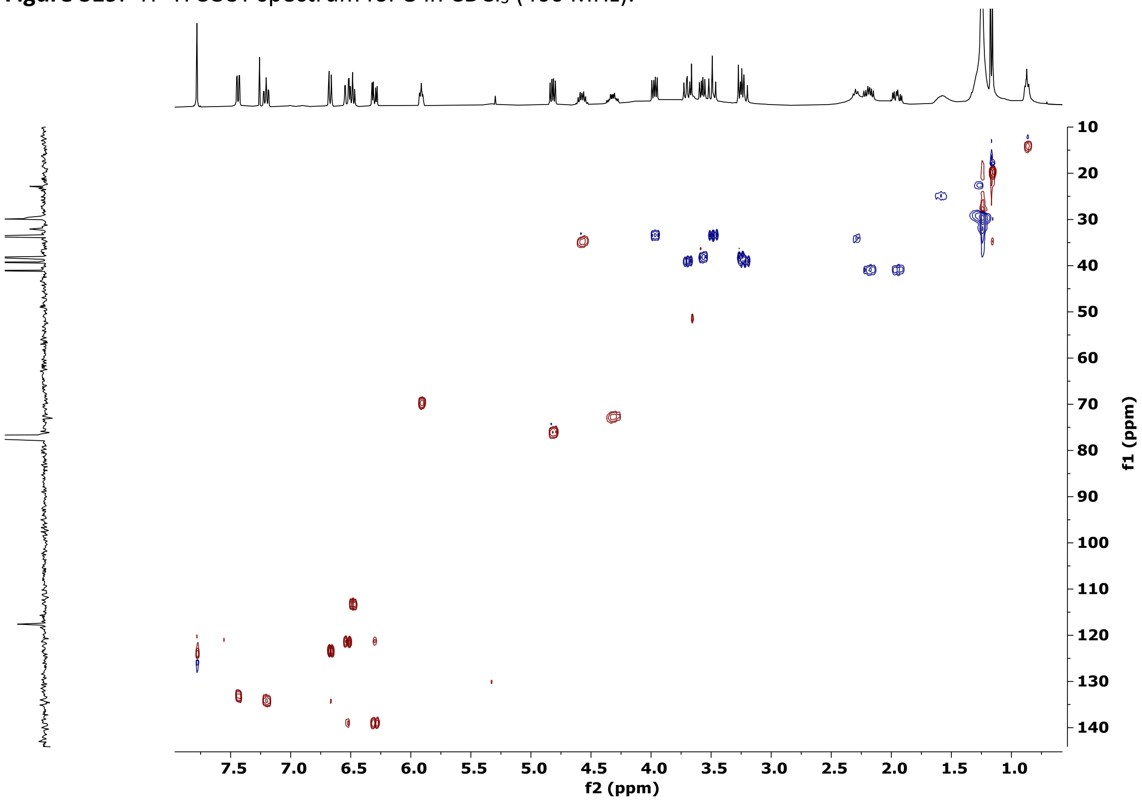

Figure S20. Multiplicity-edited HSQC spectrum for **3** in $CDCl_3$ (400 MHz, 101 MHz)

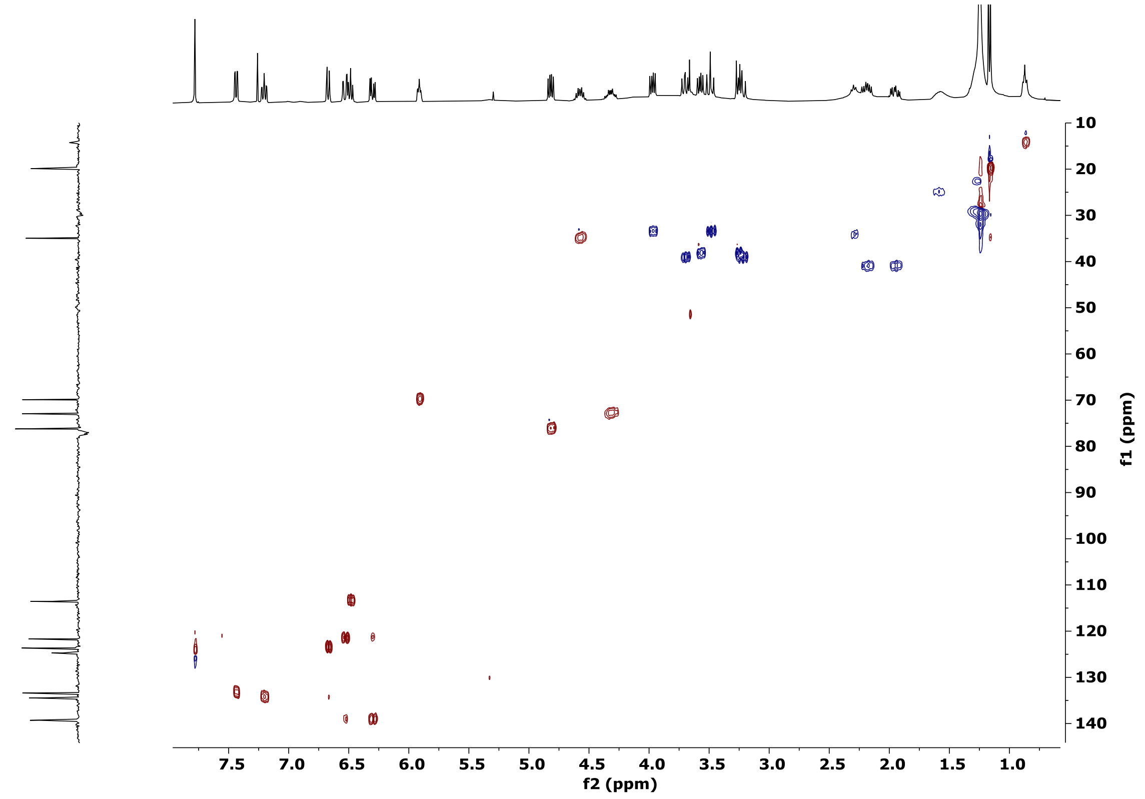

Figure S21. Multiplicity-edited HSQC spectrum for **3** in $CDCl_3$ (400 MHz, 101 MHz)

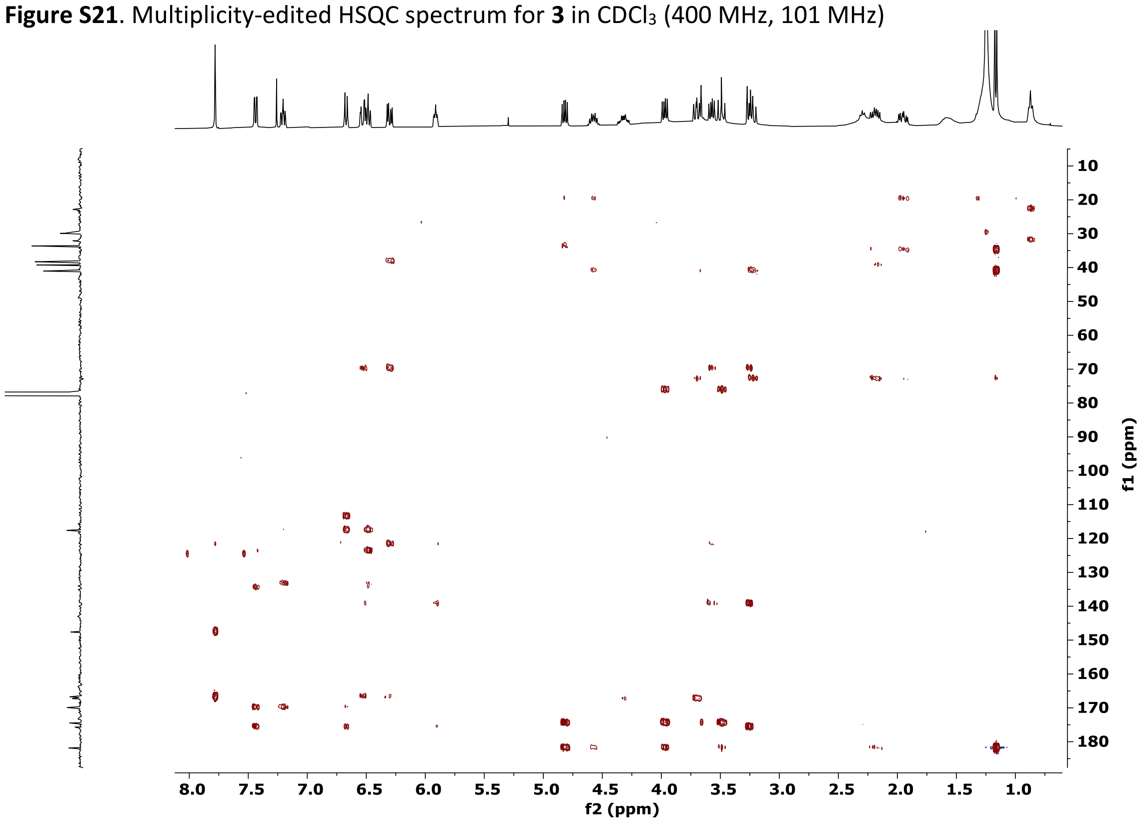

Figure S22. HMBC spectrum for **3** in $CDCl_3$ (400 MHz, 101 MHz)

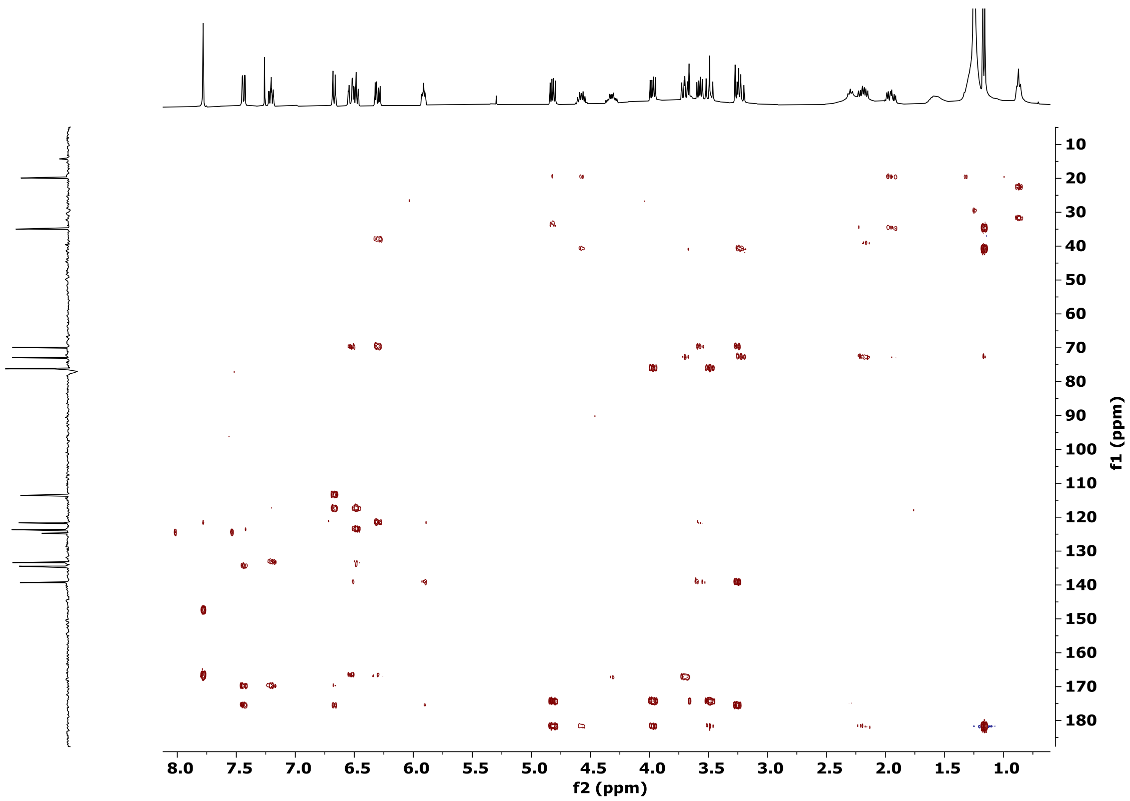

Figure S23. HMBC spectrum for **3** in $CDCl_3$ (400 MHz, 101 MHz)

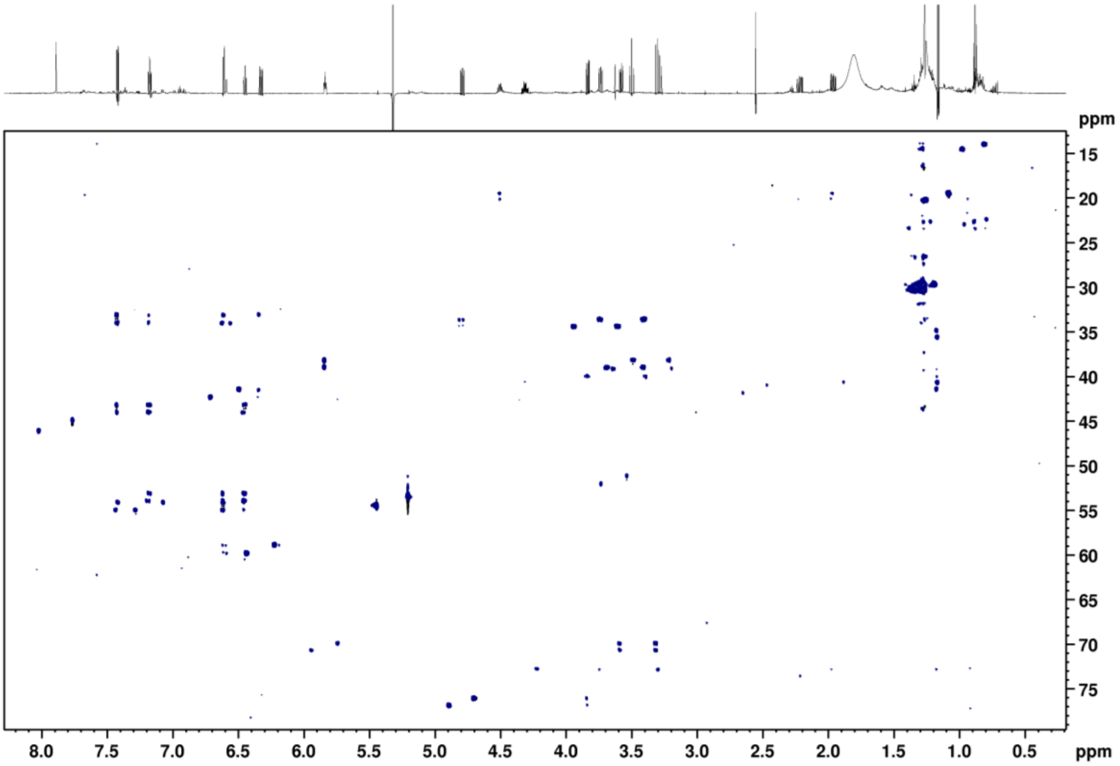

Figure S24. HSQC-TOCSY HECADE spectrum for **3** in $CDCl_3$ (750 MHz, 187.5 MHz)

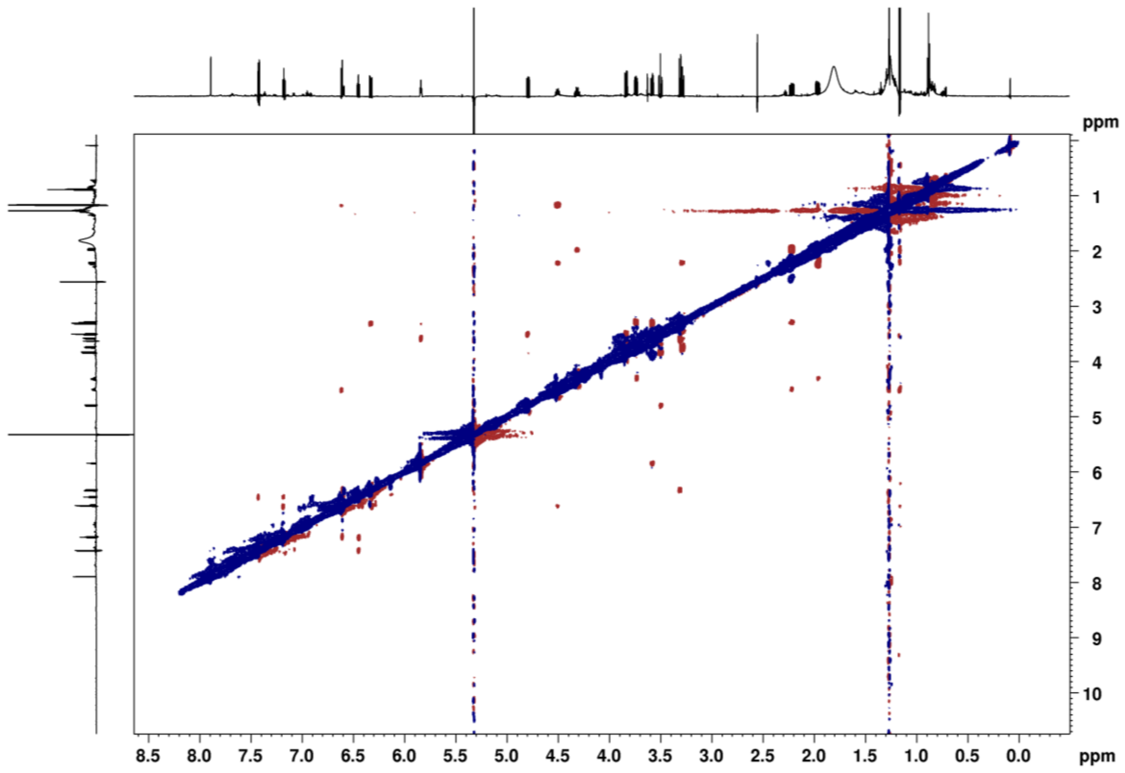

Figure S25. NOESY spectrum of **3** in CD_2Cl_2 (750 MHz).

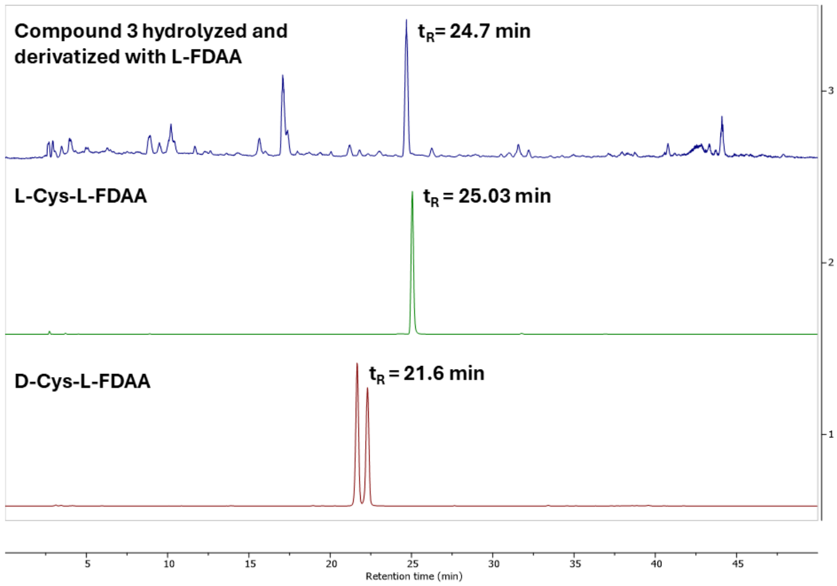

Figure S26. LC-MS analysis of Marfey's derivatives.

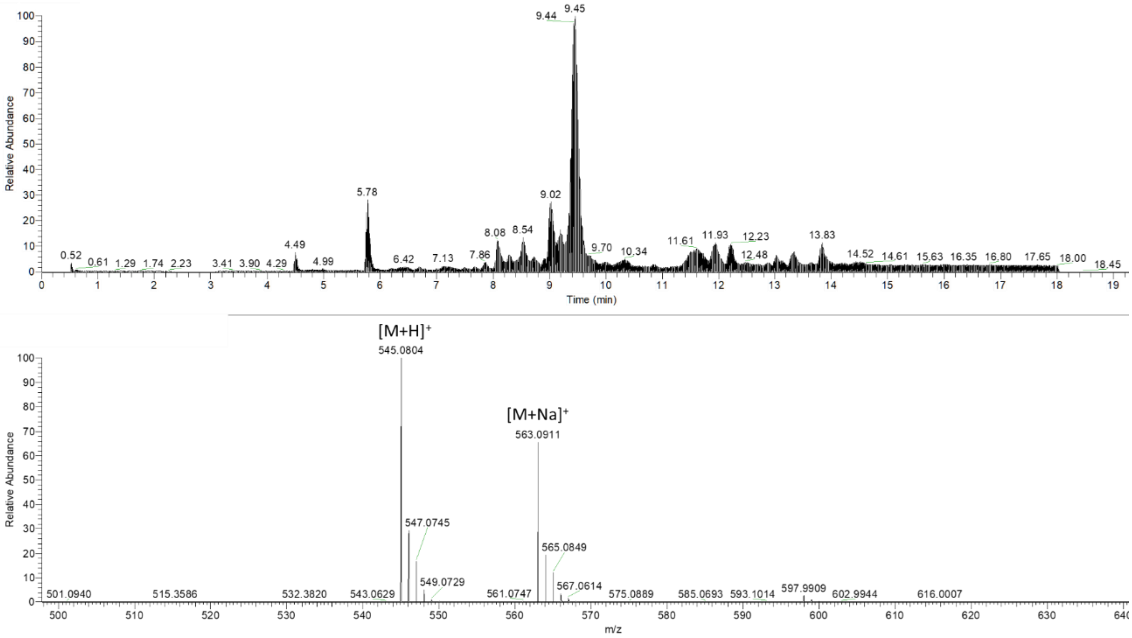

Figure S27. Total ion chromatogram (ESI positive mode) and the HRESIMS of **3**.

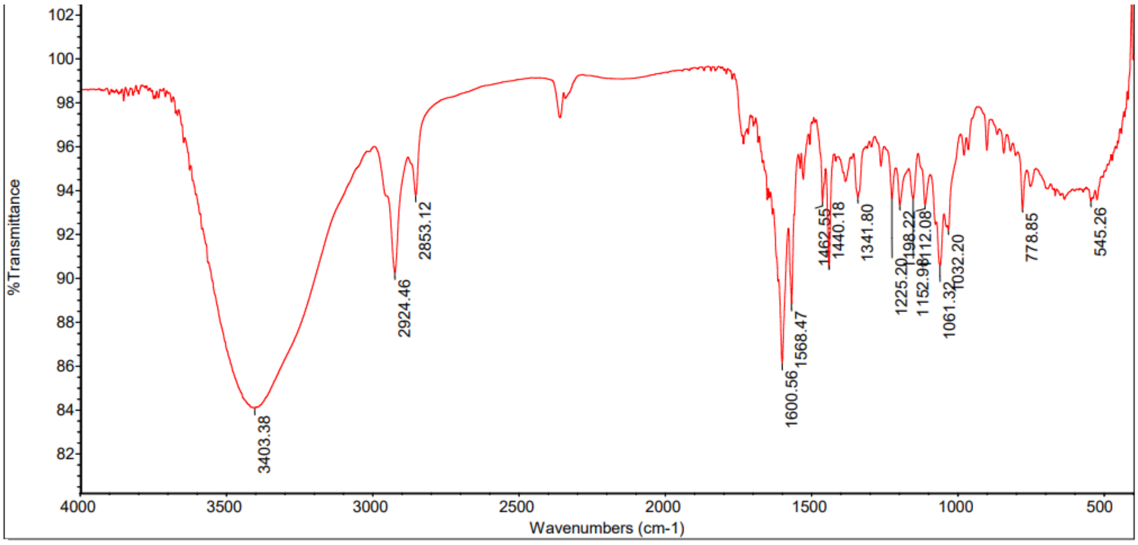

Figure S28. IR spectrum of **3**.

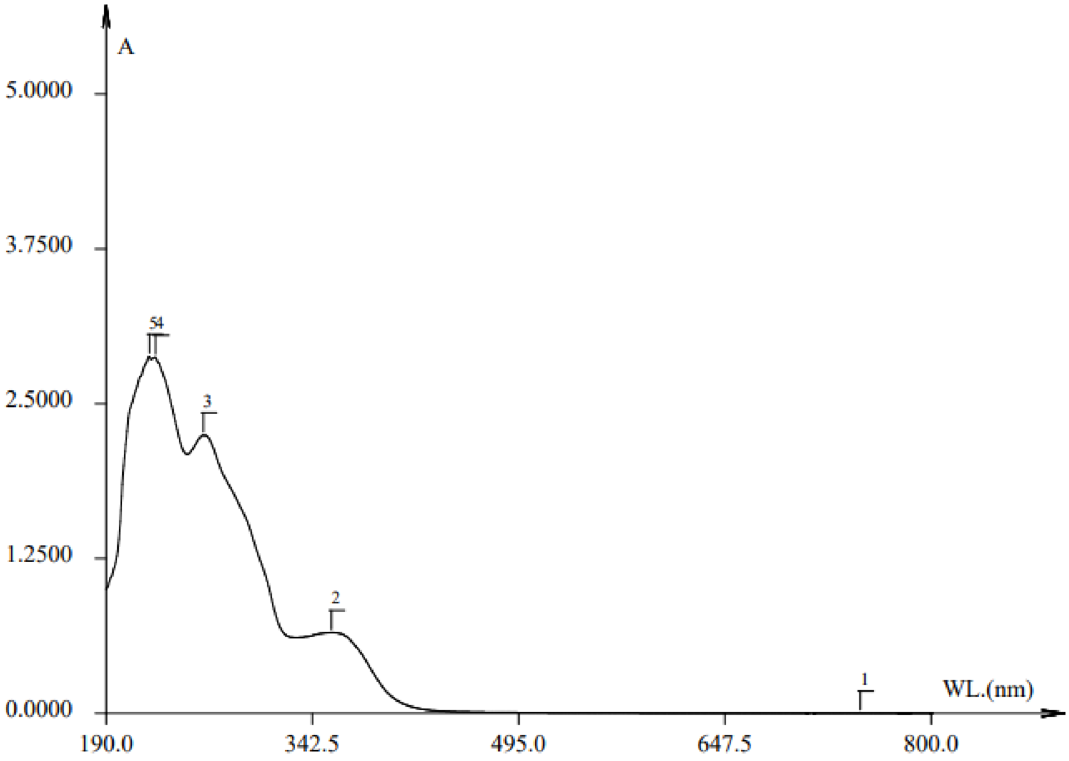

Peak List

ID	WL(nm)	Abs.	%T
1	747.5	0.0013	99.70
2	356.5	0.6513	22.32
3	262.0	2.2462	0.57
4	226.5	2.8738	0.13
5	222.0	2.8816	0.13

Figure S29. UV spectrum of **3**

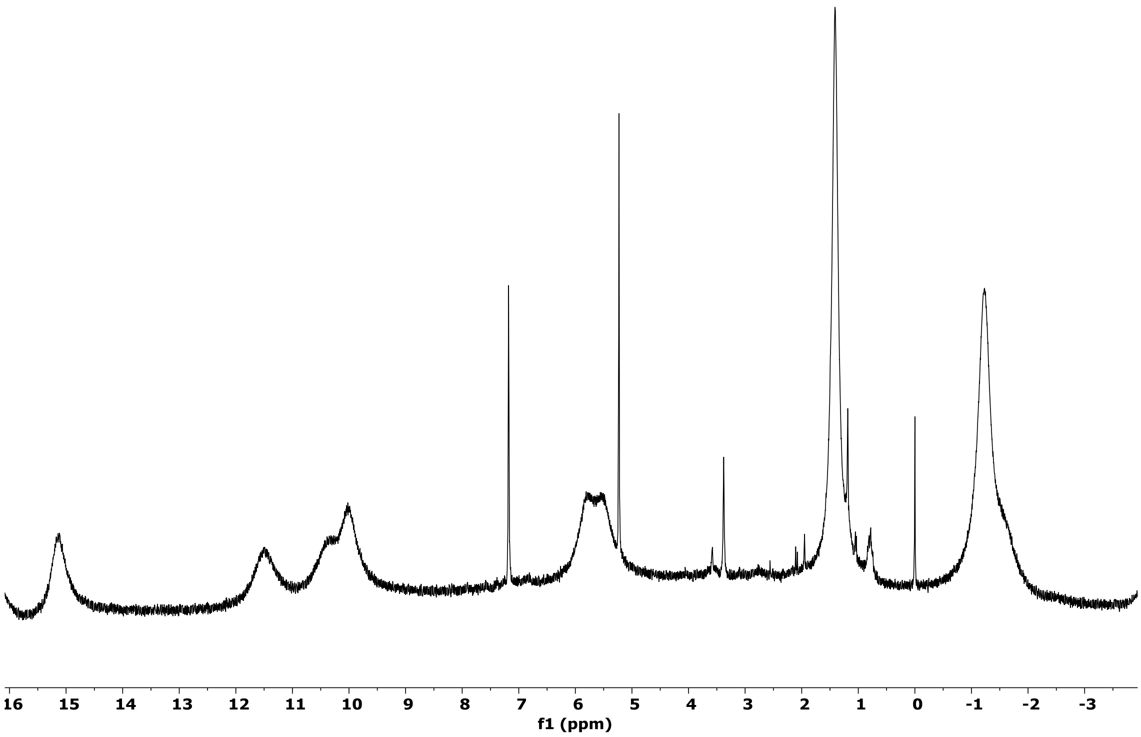

Figure S30. ^{1}H NMR spectrum for the complex **3-Fe** in $CDCl_3$ (400 MHz).

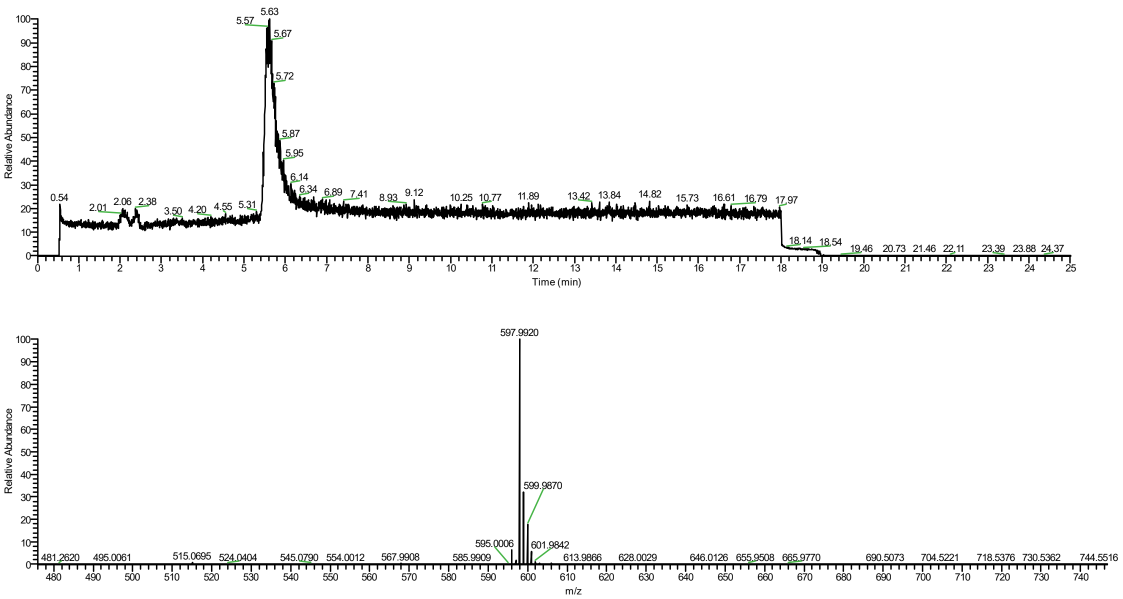

Figure S31. Total ion chromatogram (ESI positive mode) and the HRESIMS of the complex **3-Fe**.

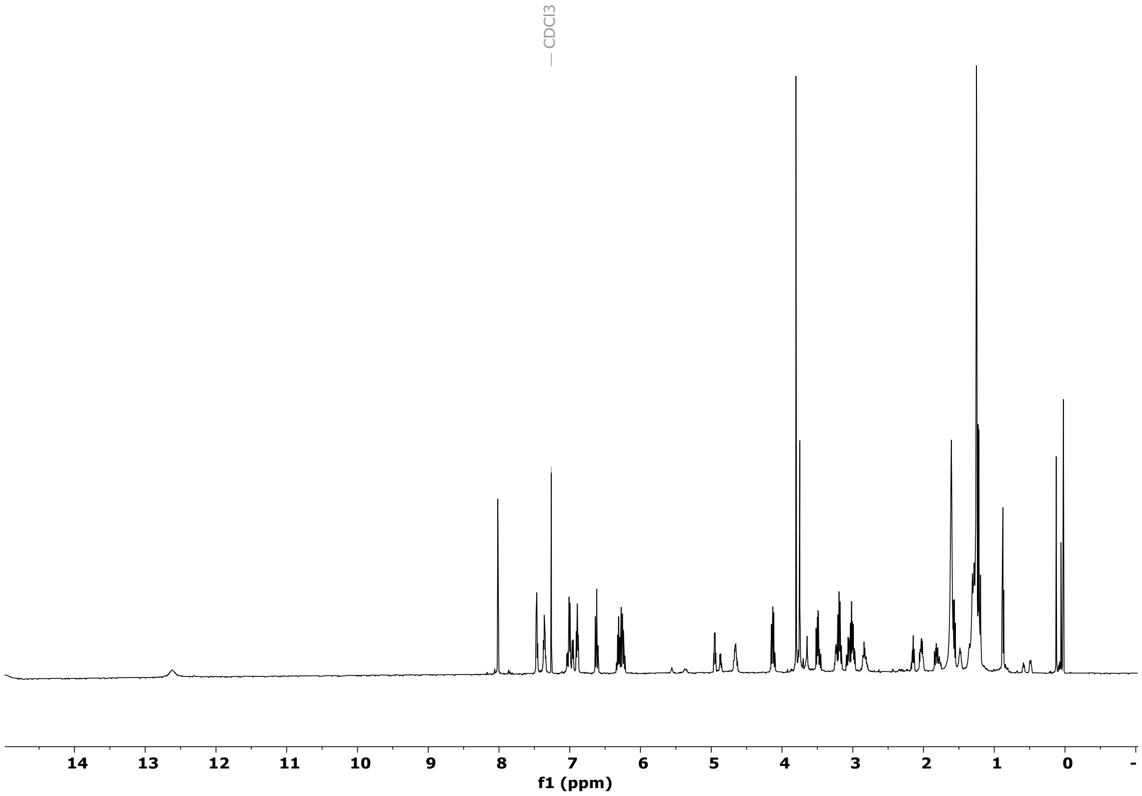

Figure S32. ^{1}H NMR spectrum for **4** in $CDCl_3$ (600 MHz).

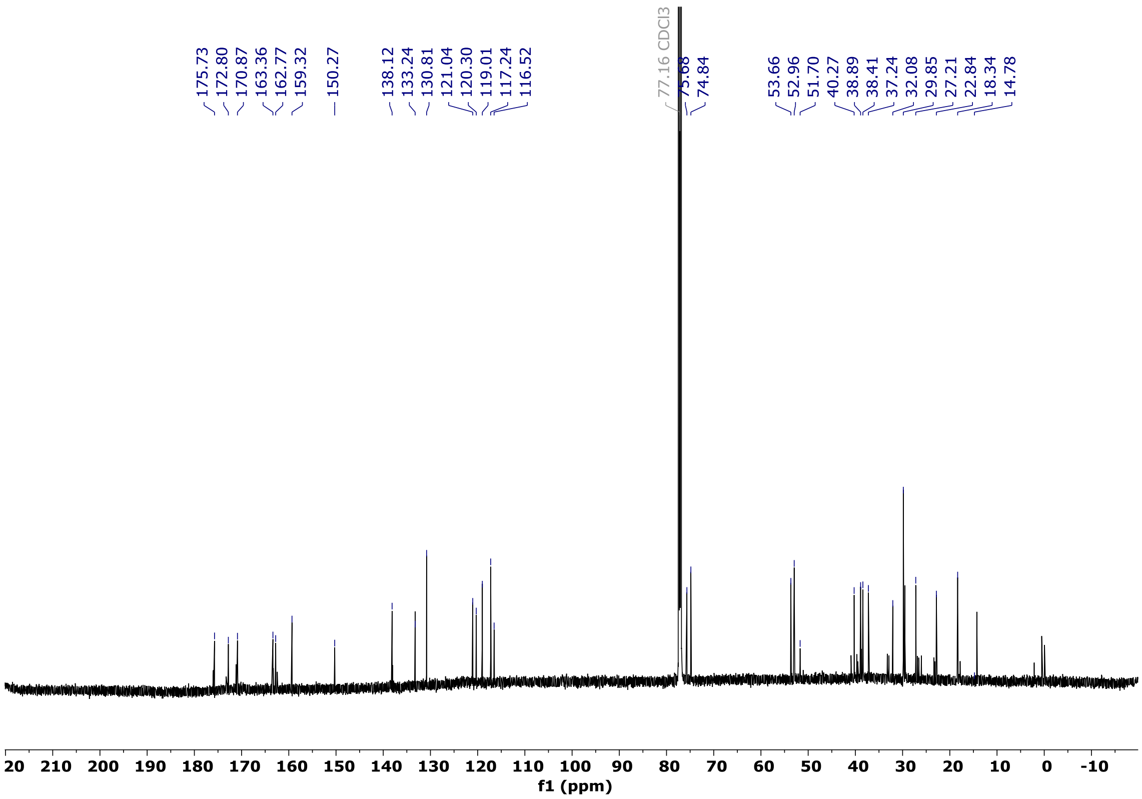

Figure S33. ^{13}C NMR spectrum for **4** in $CDCl_3$ (151 MHz).

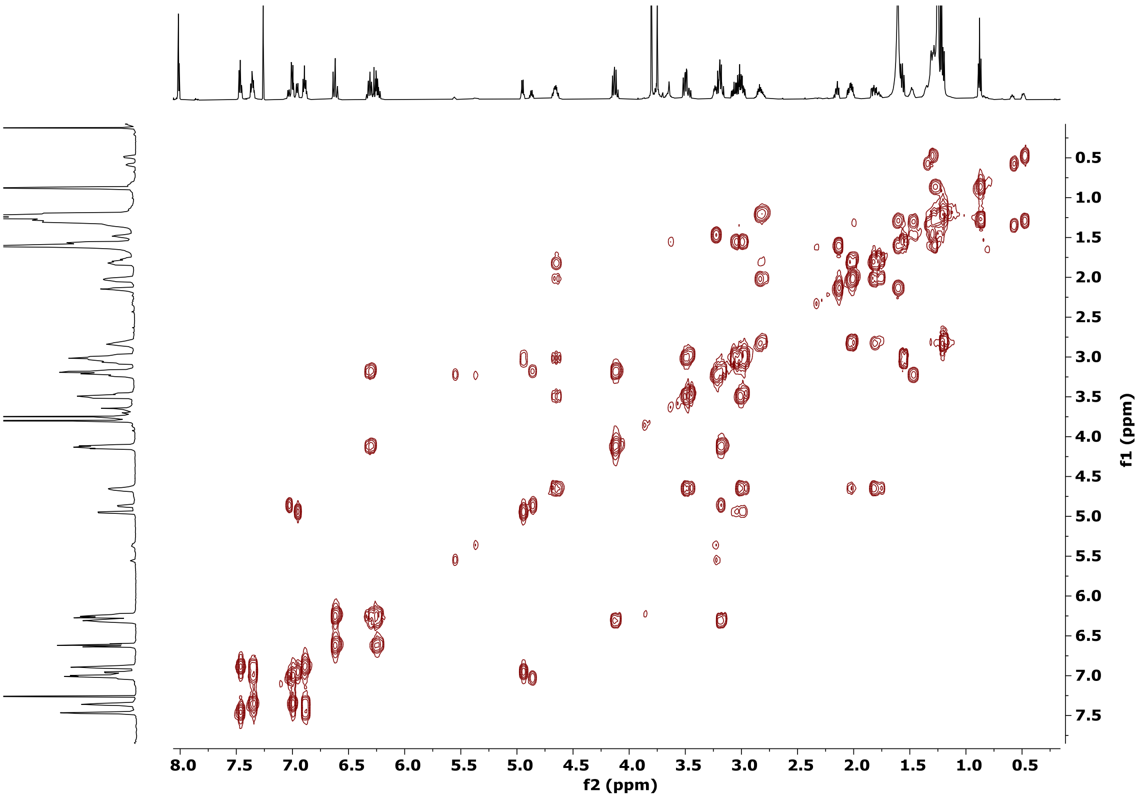

Figure S34. ^{1}H-^{1}H COSY spectrum for **4** in $CDCl_3$ (600 MHz).

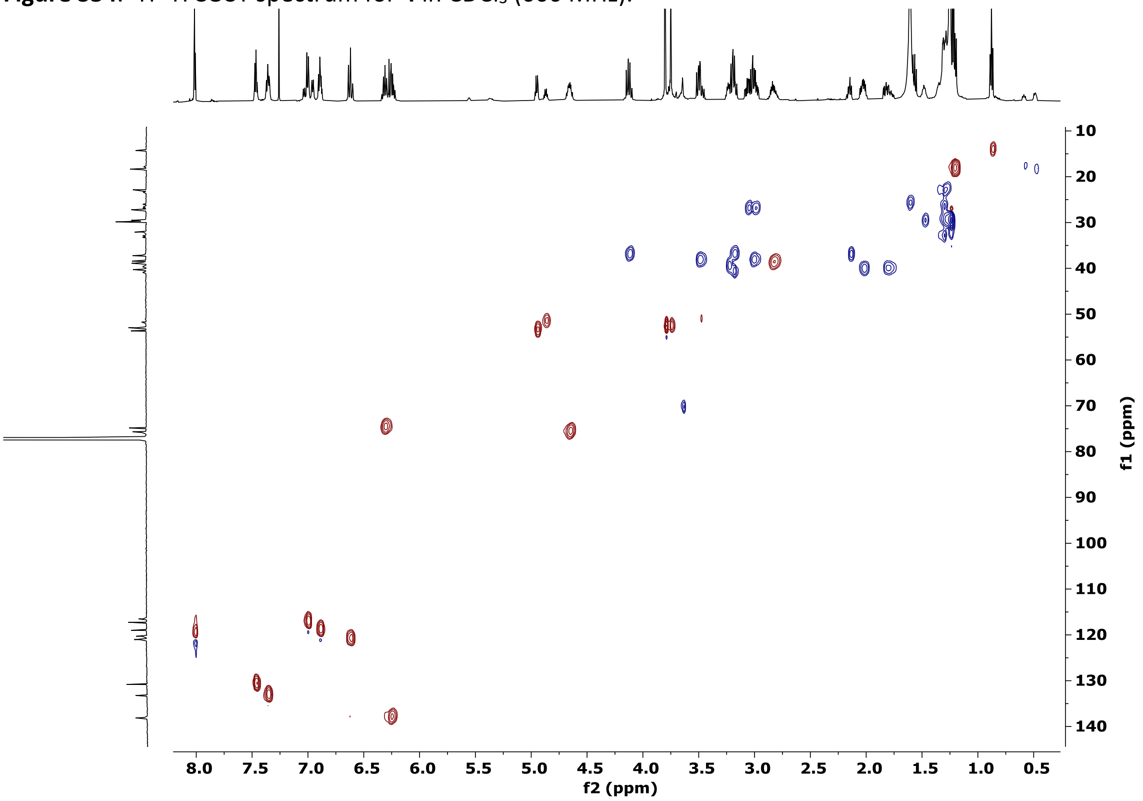

Figure S35. Multiplicity-edited HSQC spectrum for **4** in $CDCl_3$ (600 MHz, 151 MHz)

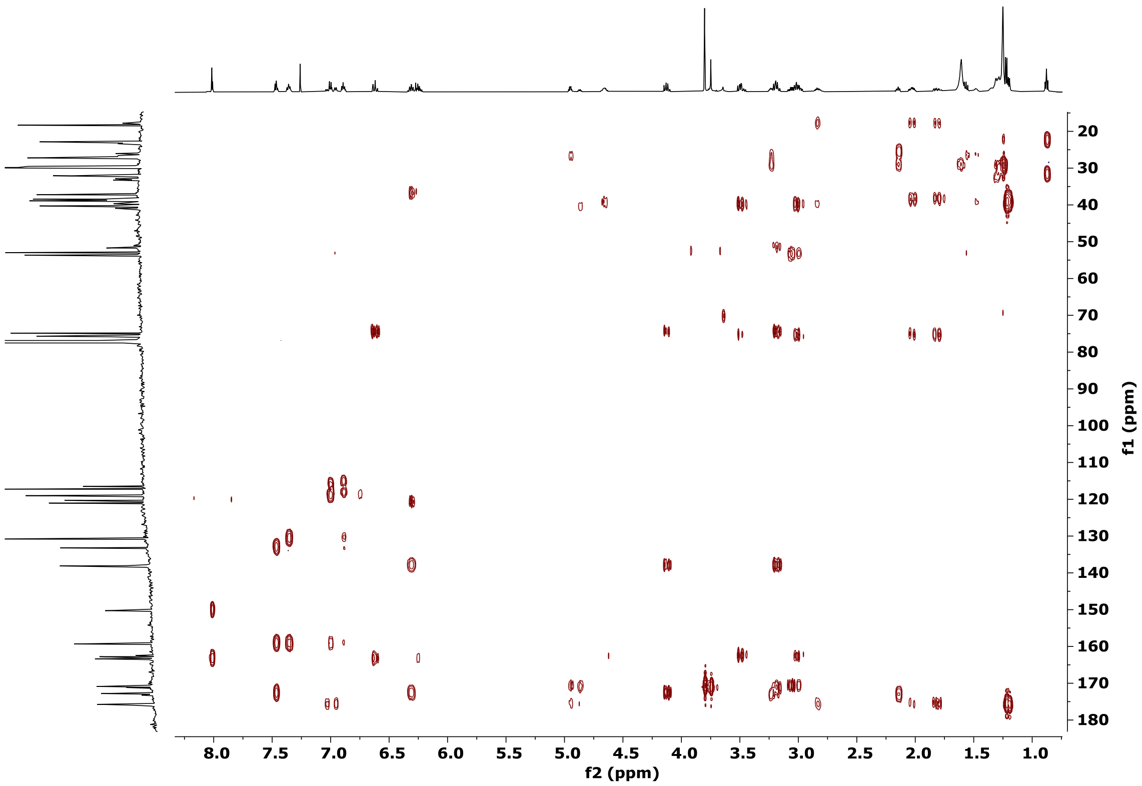

Figure S36.HMBC spectrum for **4** in $CDCl_3$ (600 MHz, 151 MHz)

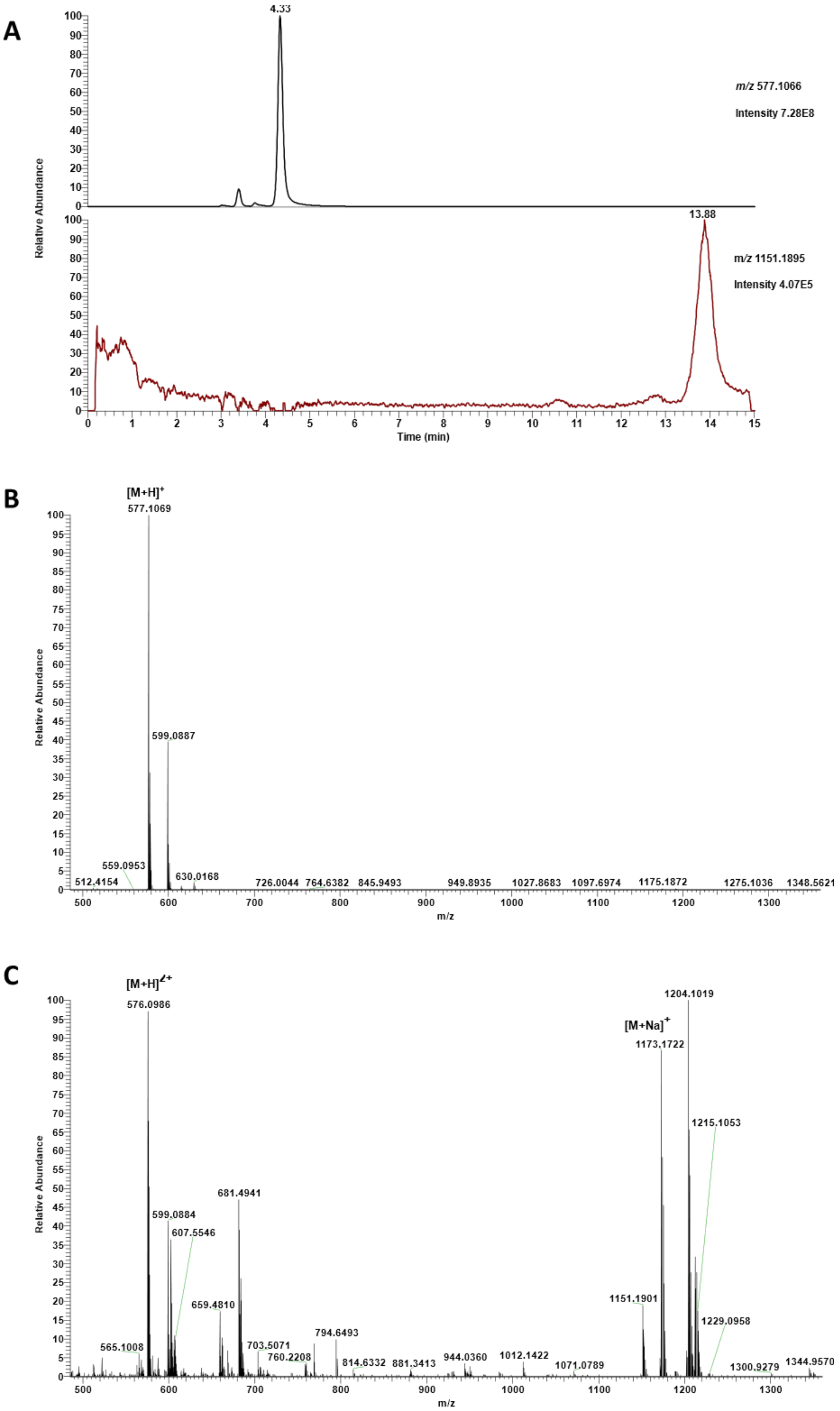

Figure S37. High-resolution mass spectrometry (HRESIMS) data of **4a** and **4b**. **(A)** Extracted ion chromatograms (within 5 ppm error) for **4a** (t_R = 4.33 min) and **4b** (t_R = 13.88 min). **(B)** (+)-HRESIMS of **4a.** (C) (+)- HRESIMS of **4b.**

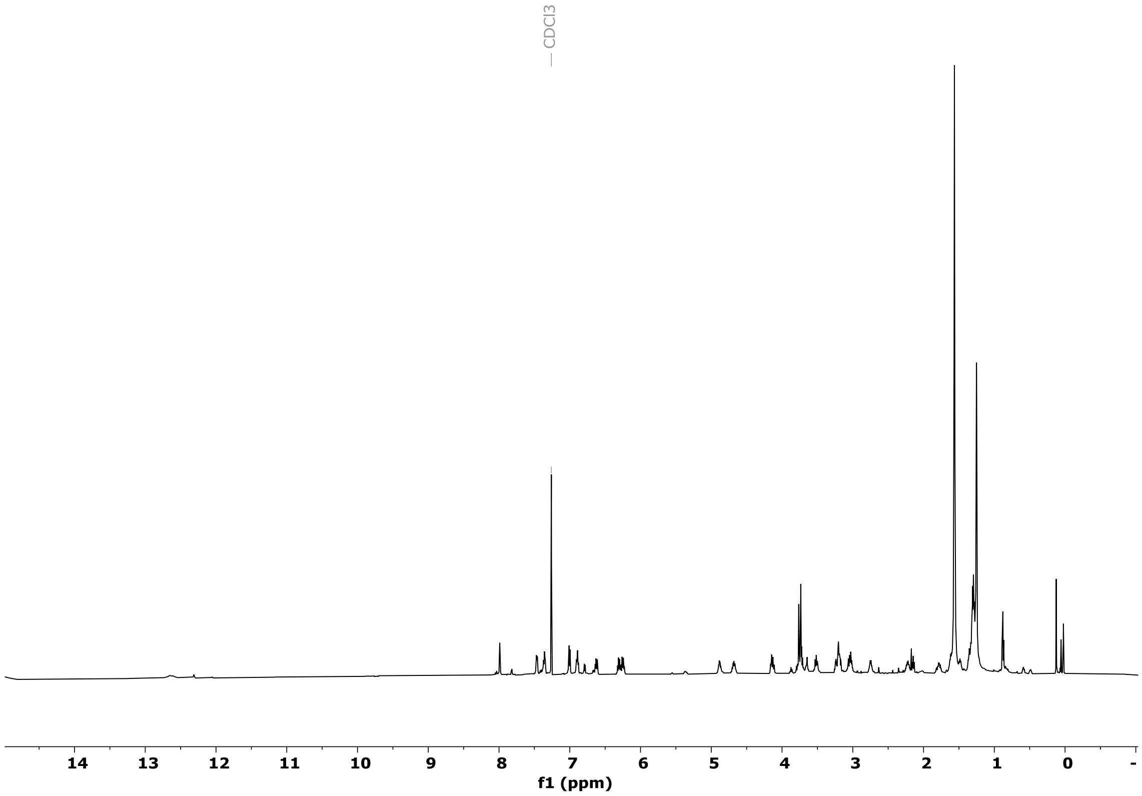

Figure S38. ^{1}H NMR spectrum for **5** in $CDCl_3$ (600 MHz).

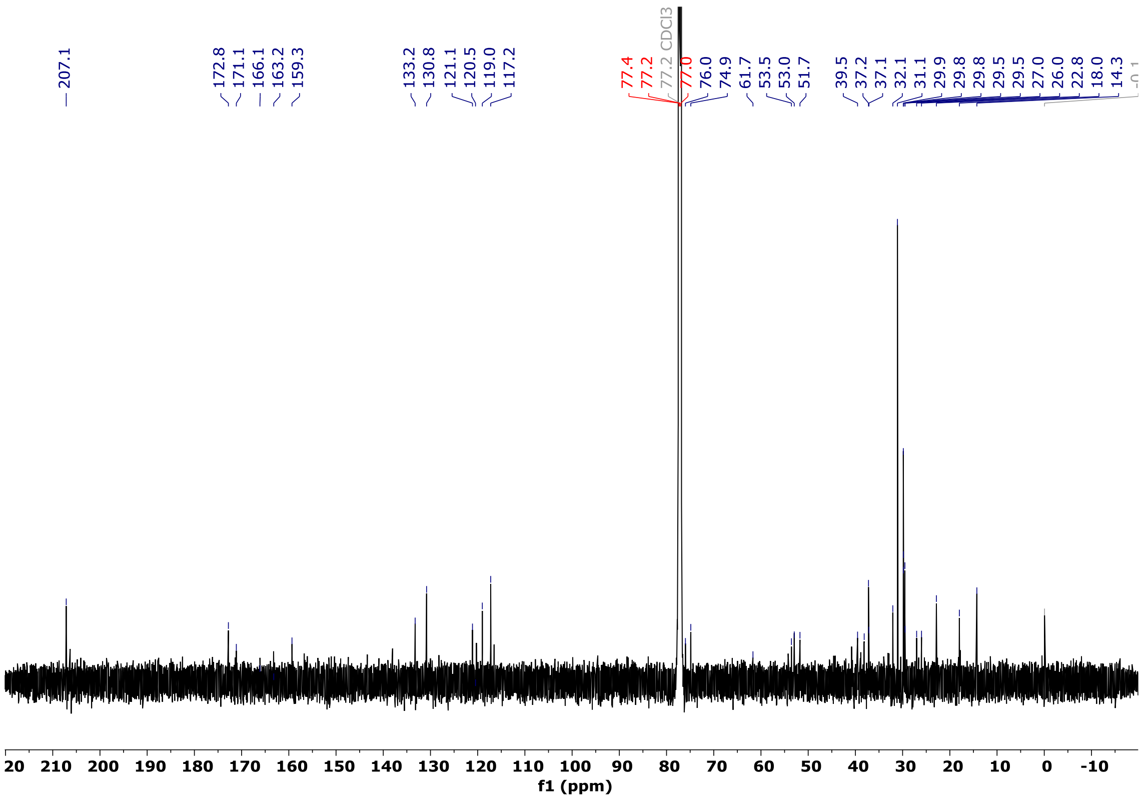

Figure S39. ^{13}C NMR spectrum for **5** in $CDCl_3$ (151 MHz).

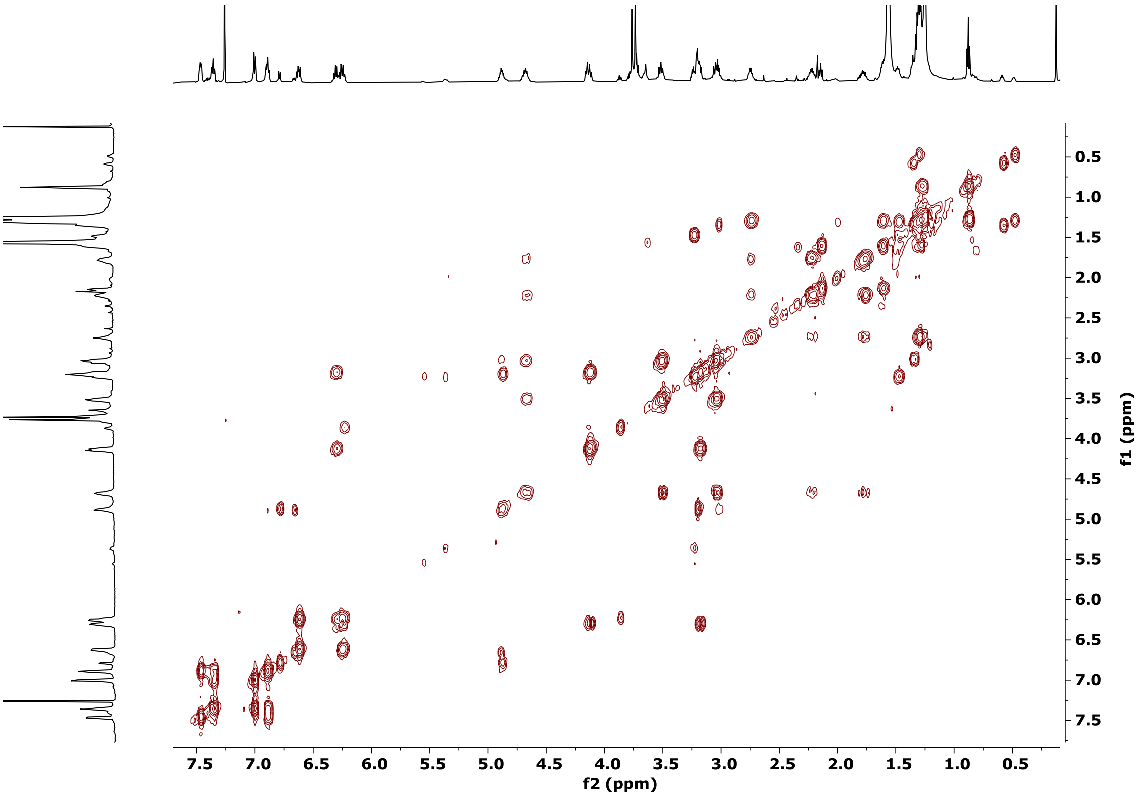

Figure S40. ^1H-^1H COSY spectrum for **5** in $CDCl_3$ (600 MHz). Moreover, the NMR spectra allowed to infer the presence of the methylester cysteine moiety in **5a** and **5b**. This observation was confirmed by the COSY correlations between the amide proton at δ_H 6.64 d (thiol **5a**)/6.79 d (sulfide dimer **5b**), and methine H22 at δ_H 4.87/ δ_C 53.1 (thiol **5a**); δ_H 4.85/ δ_C 51.0 (sulfide dimer **5b**) and in turn to methylene H21 at δ_H 3.00/ δ_C 26.2 (thiol **5a**); δ_H 3.17/ δ_C 40.0 (sulfide dimer **5b**).

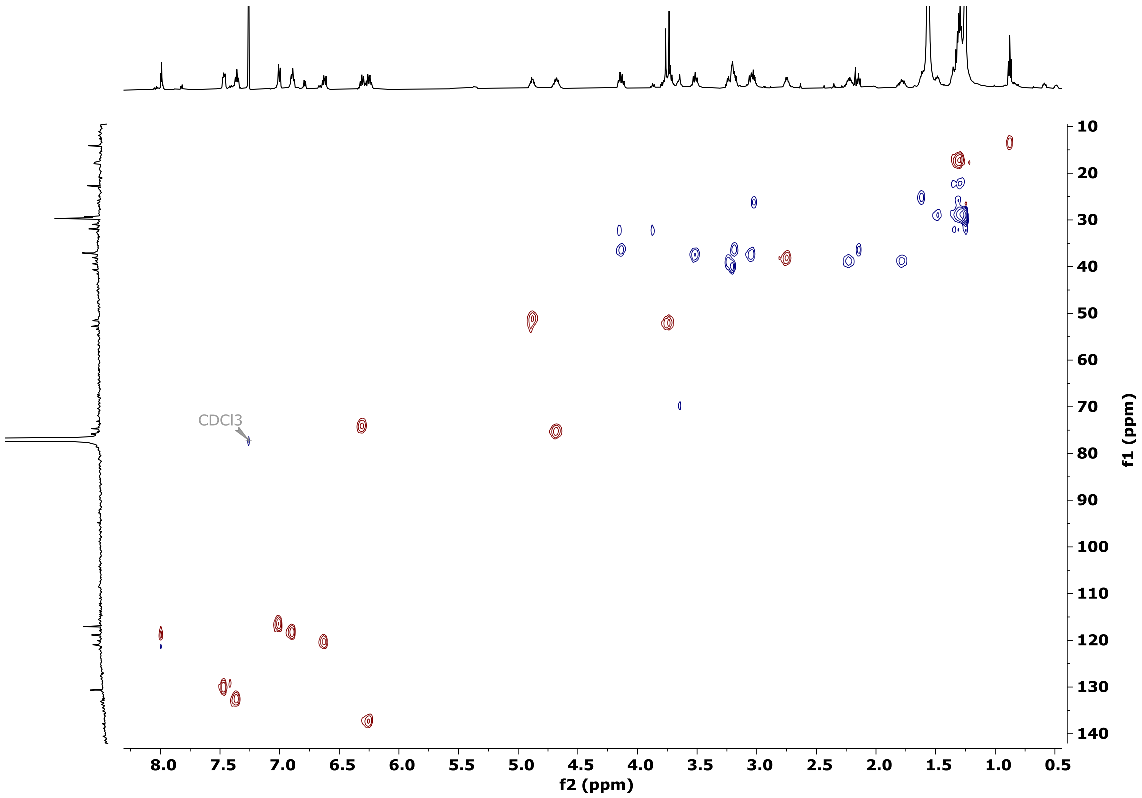

Figure S41. Multiplicity-edited HSQC spectrum for **5** in $CDCl_3$ (600 MHz, 151 MHz)

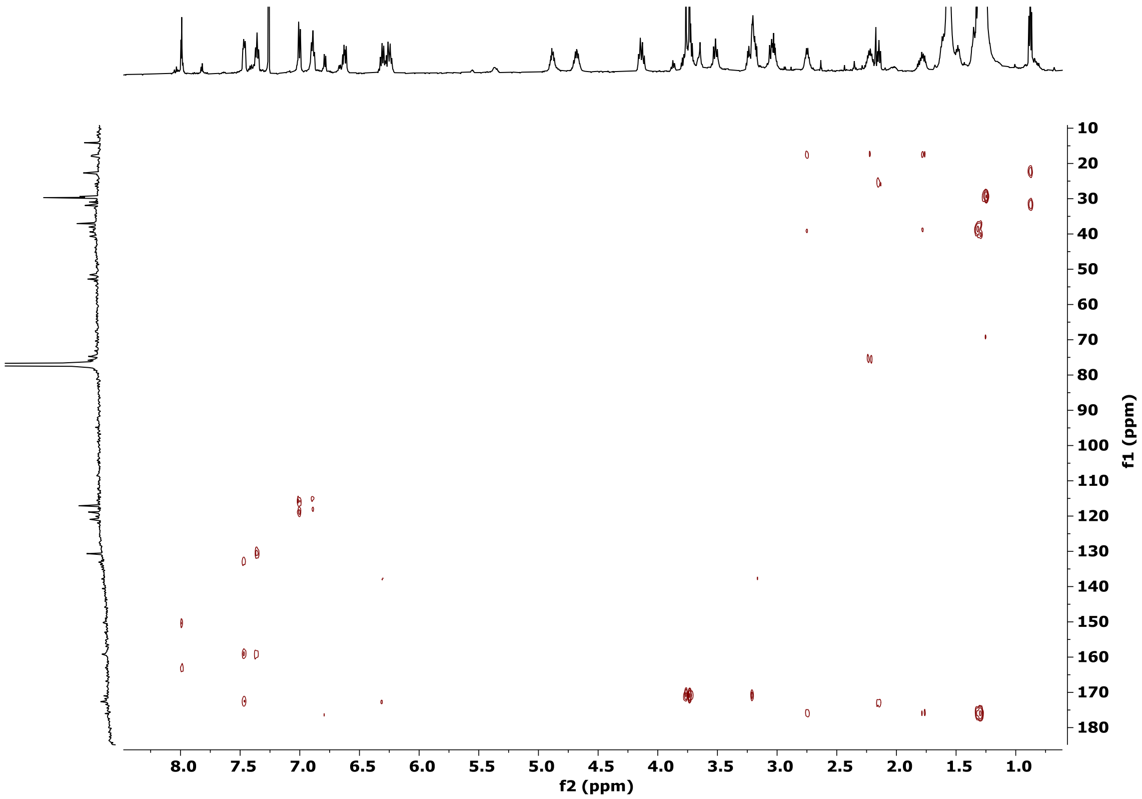

Figure S42.HMBC spectrum for **5** in $CDCl_3$ (600 MHz, 151 MHz)

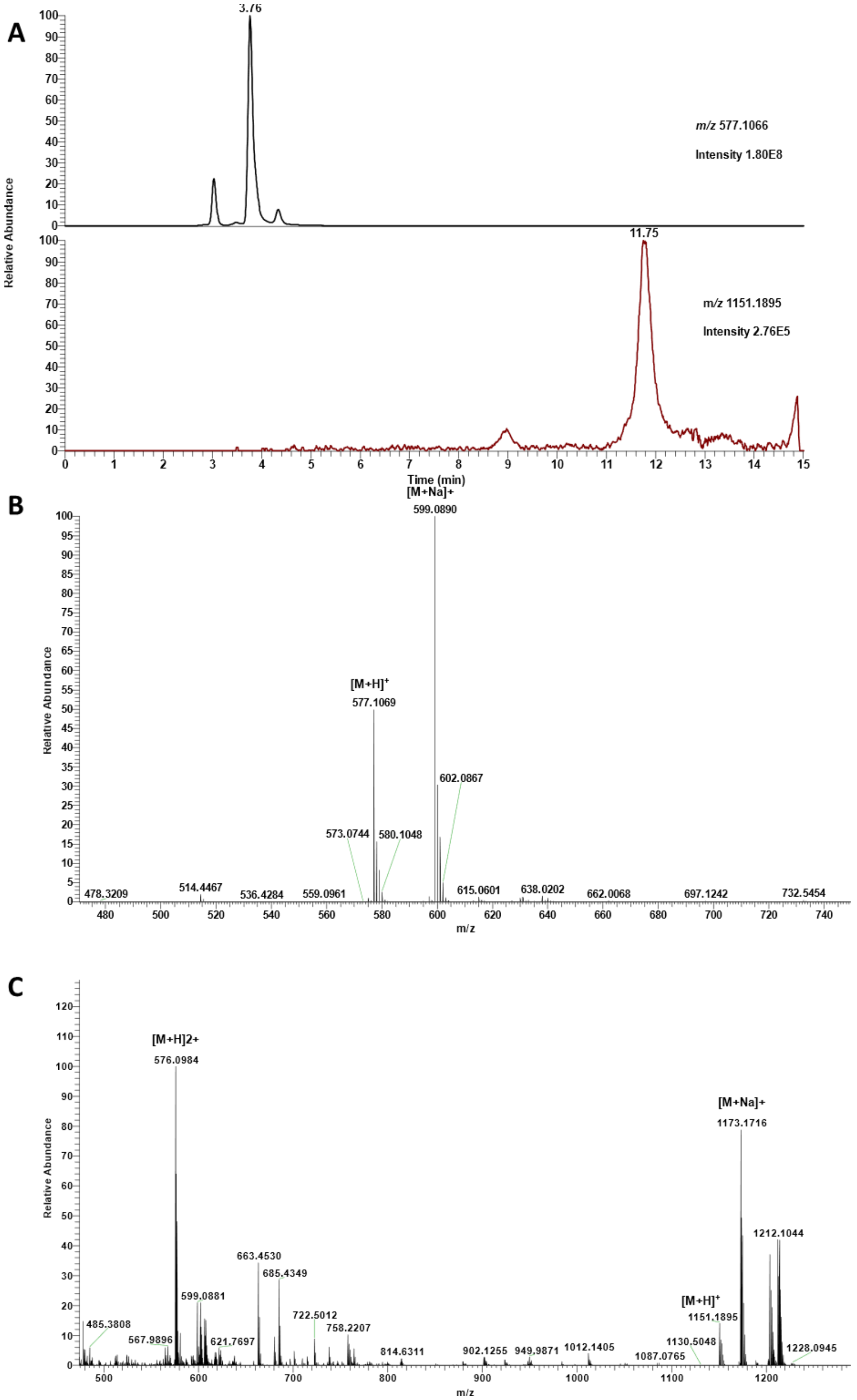

Figure S43. High-resolution mass spectrometry data of **5a** and **5b**. **(A)** Extracted ion chromatograms (within 5 ppm error) for **5a** (t_R = 4.76 min) and **5b** (t_R = 11.75 min). **(B)** (+)-HRESIMS of **5a. (C)** (+)- HRESIMS of **5b.** The total ion chromatogram showed a peak at t_R 3.75 min that displayed the $[M+H]^+$ ion at *m/z* 577.1069 in its corresponding (+)-HRESIMS which assigned to the thiol **5a**. Similarly, another peak at t_R 11.76 min was observed in that chromatogram that showed a $[M+H]^+$ ion at *m/z* 1151.1895 and $[M+H]^{2+}$ ion at *m/z* 576.0984 in its (+)-HRESIMS that was assigned to the sufide **5b**.

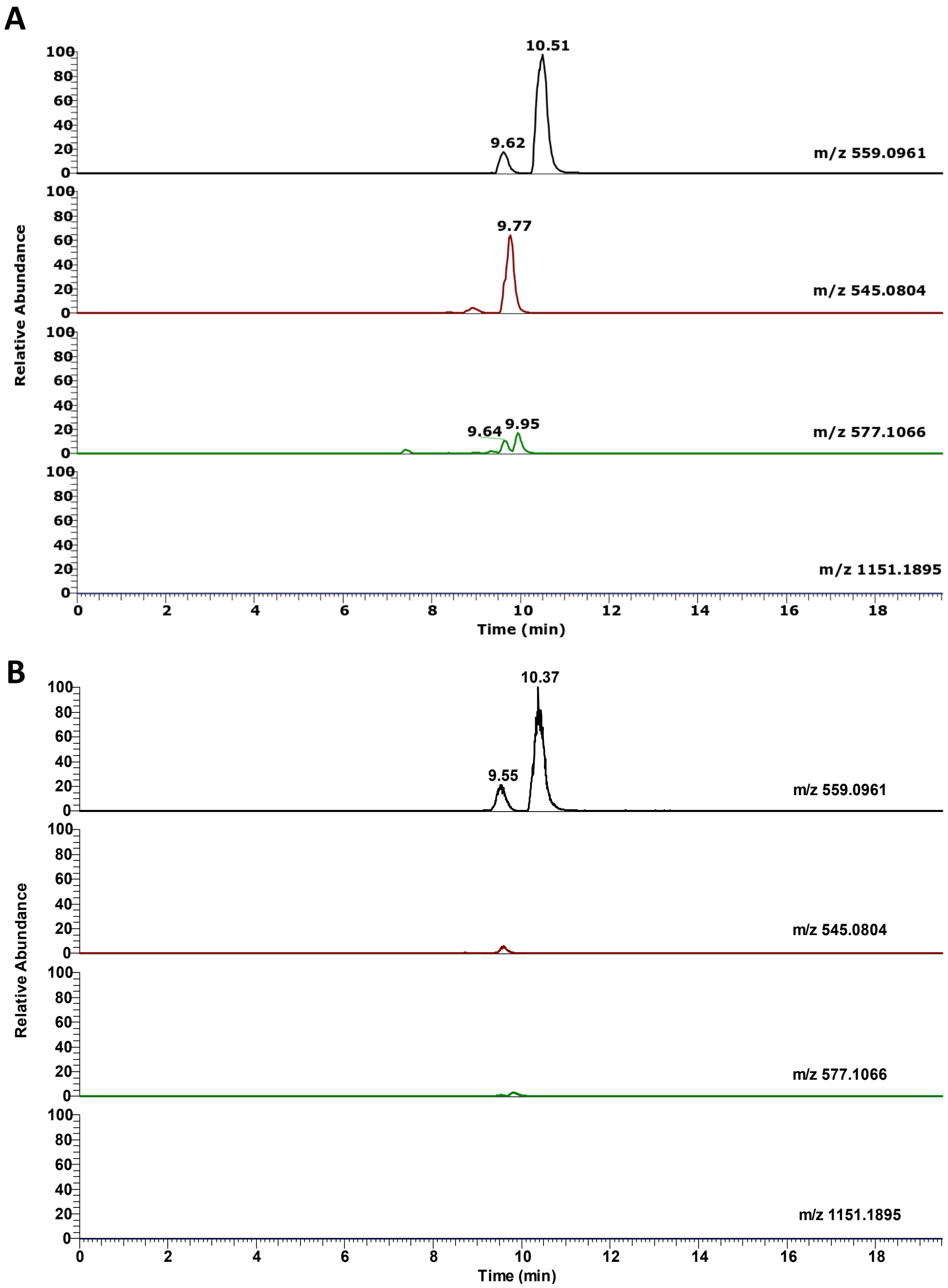

Figure S44. Comparison of the extracted ion chromatograms of the masses corresponding to lusichelins **1-5b** using different extraction methods (**A**) Data obtained from biomass extracted with MeOH, relative abundance scale was normalized to the highest intensity mass at 1.76E9, showing the presence of lusichelins **1-5a**. (**B**) Data obtained from biomass extracted with $CHCl_2$, relative abundance scale was normalized to the highest intensity mass at 1.18E9, showing that lusichelins **1-2** are the most abundant compounds in the extract.

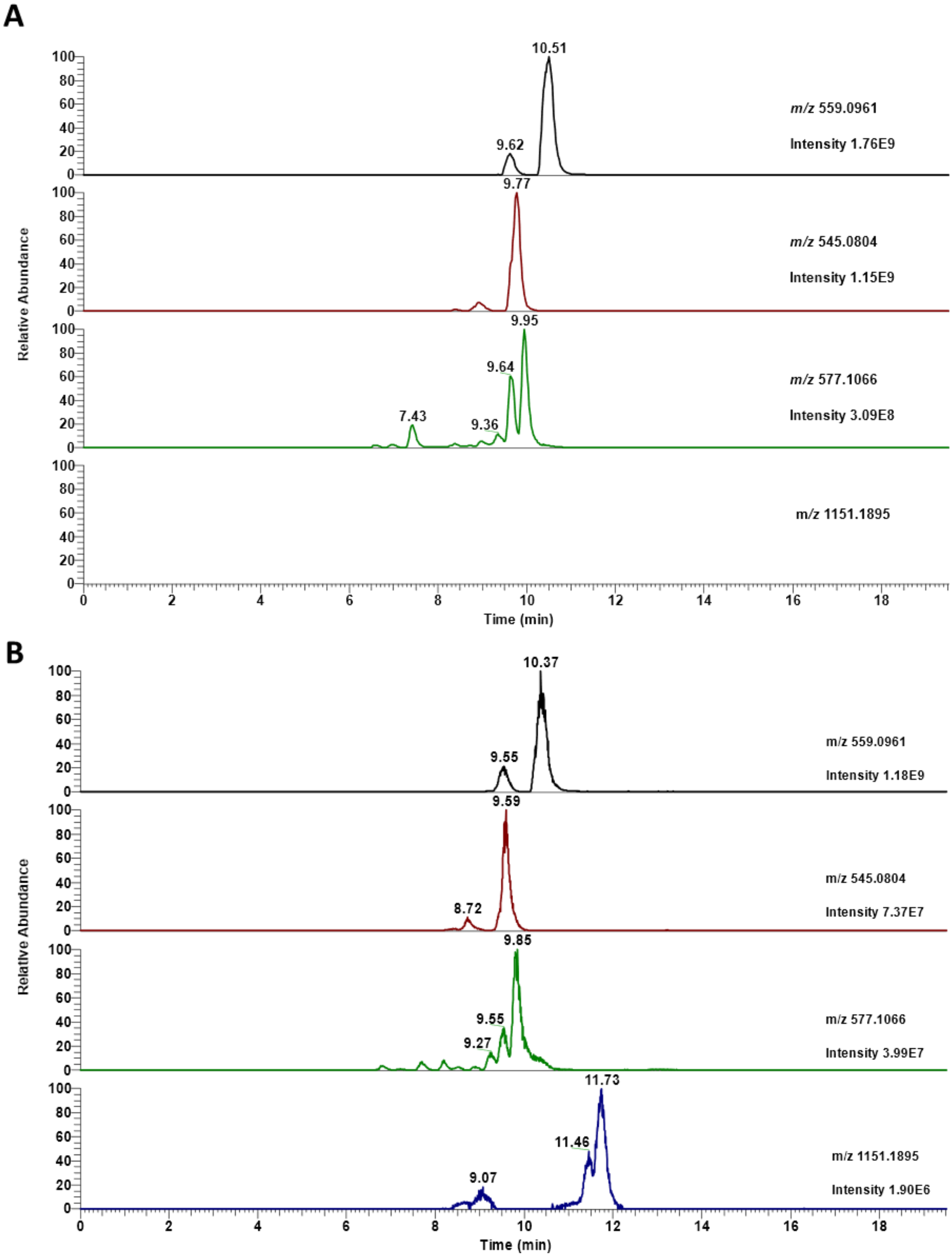

Figure S45. Comparison of the extracted ion chromatograms of the masses corresponding to lusichelins **1-5b** using different extraction methods (**A**) Data obtained from biomass extracted with MeOH, relative abundance scale was normalized to the highest intensity mass in each plot, showing the presence of lusichelins **1-5a**. (**B**) Data obtained from biomass extracted with $CHCl_2$, relative abundance scale was normalized to the highest intensity mass in each plot, showing the presence of lusichelins **1-5b**.

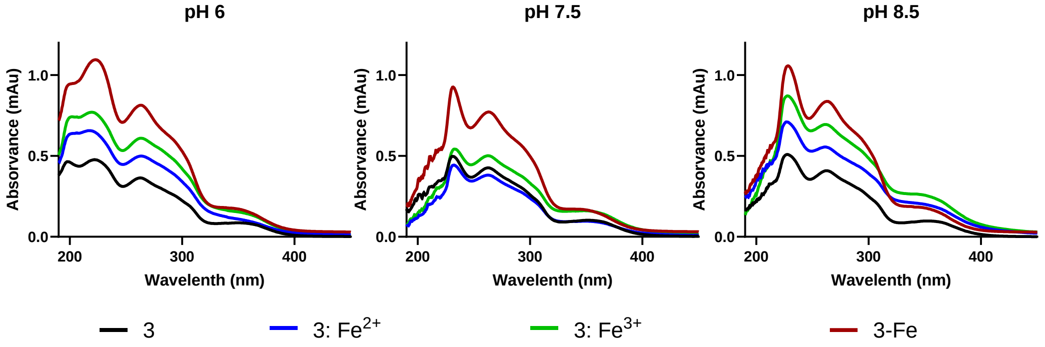

Figure S46. UV–vis absorbance spectra of **3** and **3-Fe**, along with binding experiments with $FeCl_2$ (Fe^{2+}) and $FeCl_3$ (Fe^{3+}) in equimolar amounts at pH 6.0, 7.5, and 8.5: compound **3** (black line), **3: Fe^{2+}** (blue line), **3: Fe^{3+}** (green line), and **3-Fe** (isolated **3-Fe** complex; red line).

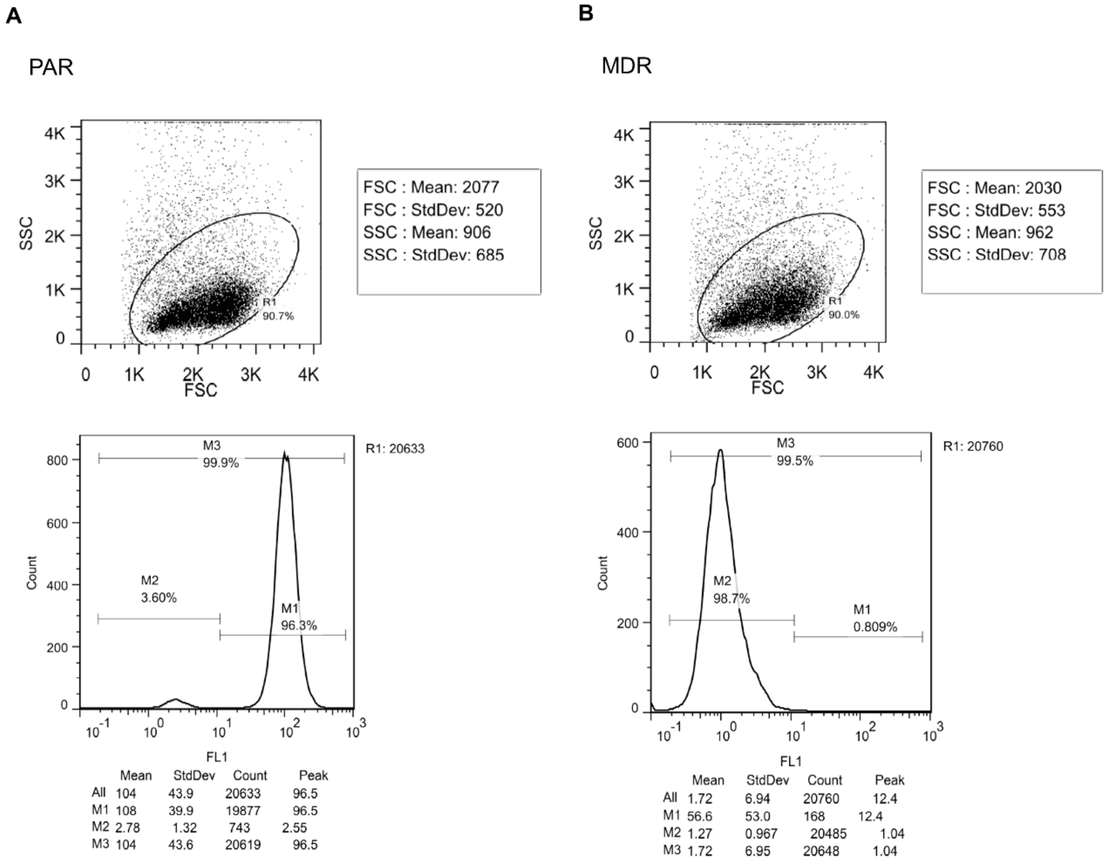

Figure S47. Modulation of ABCB1-mediated rhodamine-123 efflux. (**A**) Parental L5178Y cells exhibit cytoplasmic accumulation of the dye, while (**B**) L5178Y-MDR cells show reduced accumulation due to the activity of the efflux pump. The Forward Scatter (FSC) versus Side Scatter (SSC) plots display the gated cell population (R1). In the corresponding histograms, the term “Count” on the y-axis represents the number of individual cells within the gated populations M1 or M2, with M3 indicating the total cell count in the sample (R1). The x-axis label “FL1” refers to the mean fluorescence intensity of rhodamine-123.

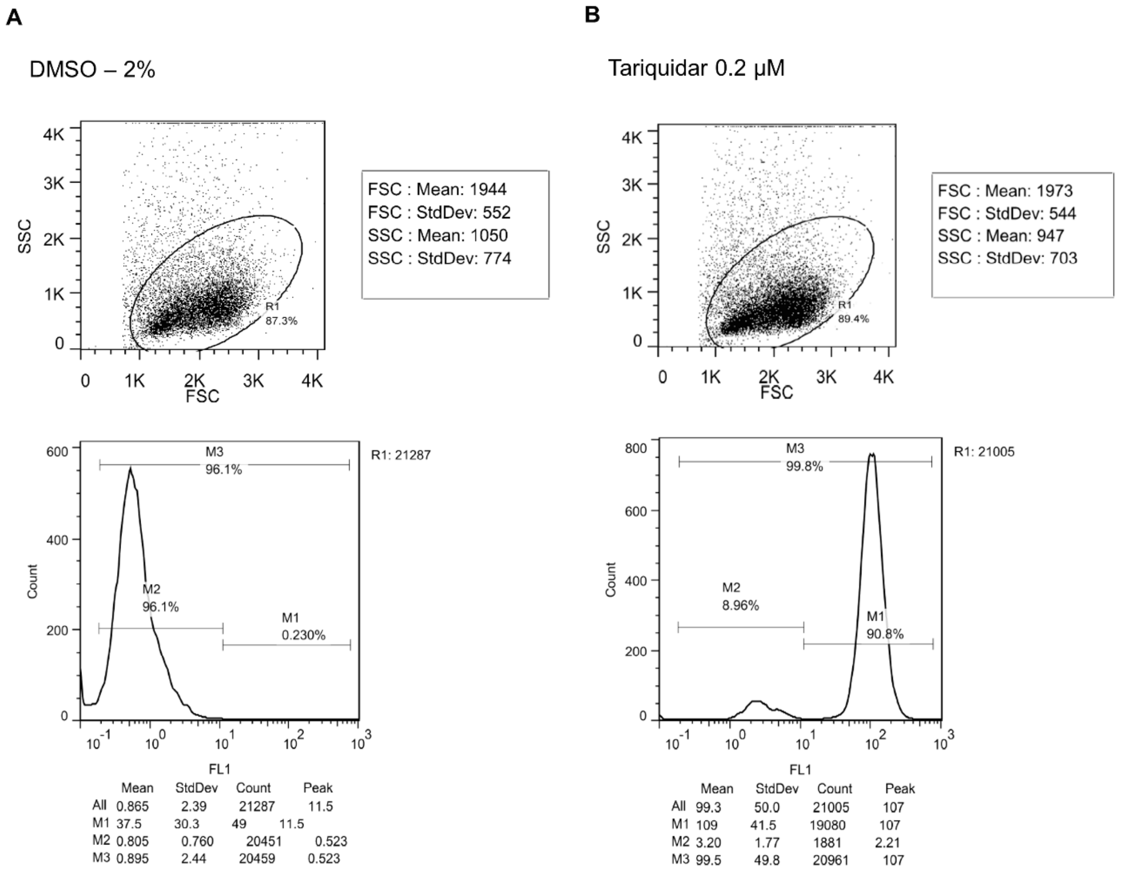

Figure S48. Modulation of ABCB1-mediated rhodamine-123 efflux. L5178Y-MDR cells exposed to (**A**) 2% DMSO (solvent control) show baseline efflux activity, as indicated by rhodamine-123 fluorescence levels. In contrast, cells treated with (**B**) 0.2 µM tariquidar, a known efflux pump inhibitor, display increased intracellular accumulation of rhodamine-123 due to inhibited efflux. The Forward Scatter (FSC) versus Side Scatter (SSC) plots depict the gated cell population (R1). In the corresponding histograms, the y-axis labeled "Count" represents the number of individual cells within the gated populations M1 or M2, while M3 indicates the total cell count of the sample (R1). The x-axis label "FL1" corresponds to the mean fluorescence intensity of rhodamine-123.

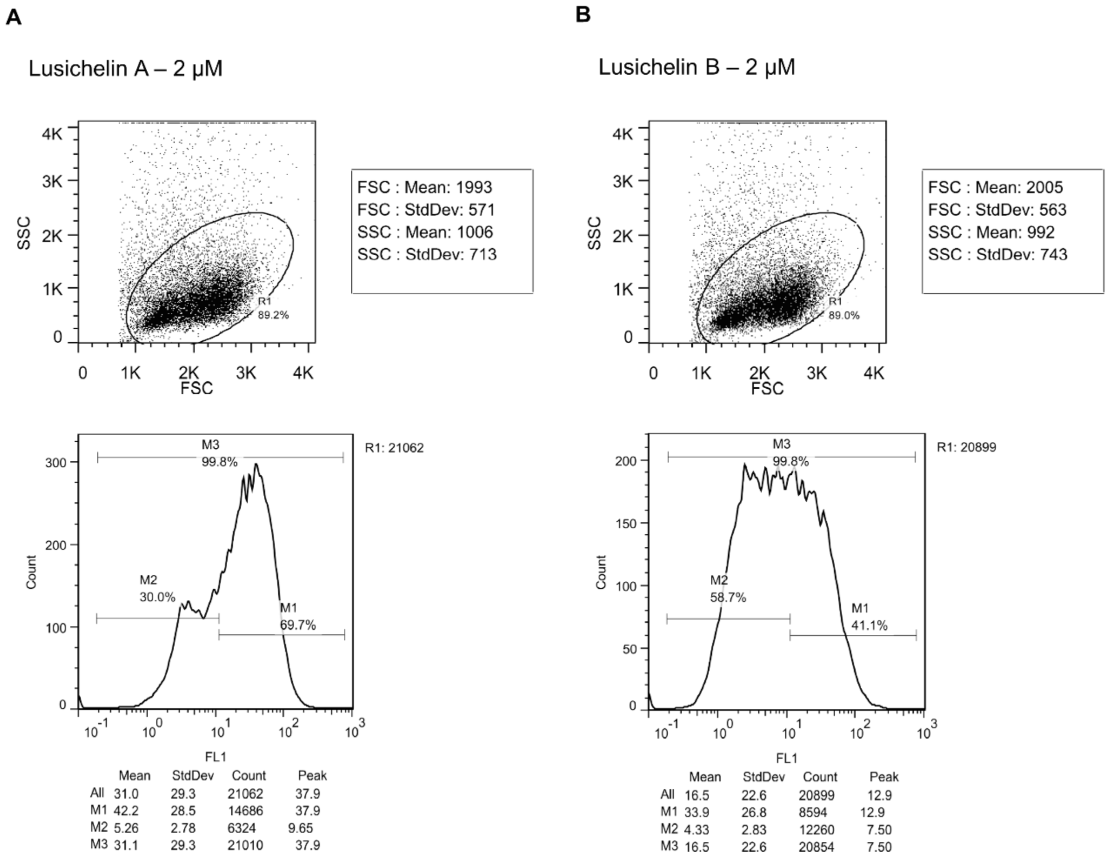

Figure S49. Modulation of ABCB1-mediated rhodamine-123 efflux. L5178Y-MDR cells exposed to 2 µM (**A**) of lusichelin A (**1**) and (**B**) lusichelin B (**2**). Both compounds effectively reversed the multidrug resistance (MDR) phenotype, with fluorescence activity ratios (FAR) of 28 and 14.9, respectively. The Forward Scatter (FSC) versus Side Scatter (SSC) plots depict the gated cell population (R1). In the corresponding histograms, the y-axis labeled "Count" represents the number of individual cells within the gated populations M1 or M2, while M3 indicates the total cell count of the sample (R1). The x-axis label "FL1" corresponds to the mean fluorescence intensity of rhodamine-123.
